# Supplementary material for: Genome-wide identification and characterization of the Hsp70 gene family in allopolyploid rapeseed (Brassica napus L.) compared with its diploid progenitors
Source: PeerJ. 2019 Aug 20;7:e7511. doi: 10.7717/peerj.7511 (PMC6707343; doi:10.7717/peerj.7511)
Supplement: Figure S1 — The amino acid sequences of Hsp70s in three Brassica species are numbered on the left. In the N-terminal ATPase domain, three typical Hsp70 signature motifs are boxed and shaded in red. In the C-terminal domain, the specific signature motifs of proteins with cytoplasm-localized, ER-localized, mitochondrion-localized and chloroplast-localized are boxed and shaded in green. [file peerj-07-7511-s001.pdf]

```
BrHsp70-2a      -----M-SG------------------------------------KGE---------------------------------
BrHsp70-2b      -----M-SG------------------------------------KGE---------------------------------
BrHsp70-2c      -----MSGG------------------------------------KGE---------------------------------
BrHsp70-2d      -----M-SG------------------------------------KGE---------------------------------
BrHsp70-2e      -----M-SG------------------------------------KGE---------------------------------
BrHsp70-2f      -----M-SG------------------------------------KGE---------------------------------
BoHsp70-2a      -----M-SG------------------------------------KGE---------------------------------
BoHsp70-2b      -----M-SG------------------------------------KGE---------------------------------
BoHsp70-2c      -----M-SG------------------------------------KGE---------------------------------
BoHsp70-2d      -----MSGG------------------------------------KGE---------------------------------
BnC.Hsp70-2     -----M---------------------------------------------------------------------------
BrHsp70-4a      -----M-AG------------------------------------KGE---------------------------------
BrHsp70-4b      -----M-AG------------------------------------KGE---------------------------------
BoHsp70-4       -----M-AG------------------------------------KGE---------------------------------
BnA.Hsp70-4a    -----M-AG------------------------------------KGE---------------------------------
BnC.Hsp70-4b    -----M-AS------------------------------------KGE---------------------------------
BnA.Hsp70-4c    -----M-AG------------------------------------KGE---------------------------------
BnC.Hsp70-4d    -----M-AG------------------------------------KGE---------------------------------
BrHsp70-5a      -----M-AT------------------------------------KSD---------------------------------
BrHsp70-5b      -----M-TT------------------------------------KSE---------------------------------
BoHsp70-5a      -----M-TT------------------------------------KSE---------------------------------
BoHsp70-5b      -----M-AT------------------------------------KSE---------------------------------
BnC.Hsp70-5a    -----M-TT------------------------------------KSE---------------------------------
BnA.Hsp70-5b    -----M-TT------------------------------------KSE---------------------------------
BnC.Hsp70-5c    ---------------------------------------------------------------------------------
BnA.Hsp70-5d    ---------------------------------------------------------------------------------
BrHsp70-6a      -----MASSA---------------------------AQIHVLGGIGFAT--TSSKRNLNSKTTLIPRSA---FFGTRTGP
BrHsp70-6b      -----MASSA---------------------------AQIHVLCGIGFTT--SSSKRNLNGKSSFMPRSA---FFGARNGP
BrHsp70-6c      -----MASST---------------------------AQIHVLGGIGFA---ASSKRNLNGKANLKPRSA---FFGTRAGP
BrHsp70-6d      -----MASSA---------------------------AQIHVLGGIGIVTSSSSSKRNLNVKESYMPRSA---FFGSRNGP
BnC.Hsp70-6a    -----MASSA---------------------------AQIHVLGGIGITT-SSSSKRNLNGKSSFMPRSA---FFGARNGP
BnC.Hsp70-6b    -----MASST---------------------------AQIHVLGGIGFA---ASSKKNLNAKANLKPRSA---FFGTRTGP
BnC.Hsp70-6c    -----MASSA---------------------------AQIHALGGIGIATYSSSSKSNLNVKGSYMPISGSCYFFGARNGP
BnC.Hsp70-6d    -----MKNHGGQEWDEAEGEHRRQRLMLNSSILRLLPAQINIHGGTGLA-ACSSSKRNLNGKVSFMPRSA---FFGTRNST
BoHsp70-7       ---------------------------------------------------------------------------------
BnA.Hsp70-7a    ---------------------------------------------------------------------------------
BnA.Hsp70-7b    ---------------------------------------------------------------------------------
BnC.Hsp70-7c    ---------------------------------------------------------------------------------
BnA.Hsp70-7d    ---------------------------------------------------------------------------------
BrHsp70-8       -----MAEP---------------------------------------AYTVASDSENTGEEKS-----------------
BoHsp70-8       -----MAEP---------------------------------------AYTVASDSENTGEEKS-----------------
BnA.Hsp70-8a    -----MAEP---------------------------------------AYTVASDSENTGEEKS-----------------
BnC.Hsp70-8b    -----MAEP---------------------------------------AYTVASDSENTGEEKS-----------------
BrHsp70-9a      -----MASV----------------------------AILRAFRRREVQTASASAFRSIATNGKTNLI---------GKLGH
BrHsp70-9b      -----MASV----------------------------ALLRALRRRELHTASVSAFKSVST--KTSLV-----------GH
BoHsp70-9a      -----MASV----------------------------AILRAFRRREVQTASVSAFRSIASNGKTNLI---------GKLGH
BoHsp70-9b      ---------------------------------------------------------------------------------
BnC.Hsp70-9a    -----MASV----------------------------ALLRALRRRELHTASVSAFKSVSTNGKTSLI---------GKLGH
BnC.Hsp70-9b    -----MASV----------------------------AILRAFRRREVQTASVSAFRSIASNGKTNLI---------GKLGH
BnA.Hsp70-9c    -----MASV----------------------------AILRAFRRREVQTASASAFRSIASNGKSNLI---------GKLGH
BnA.Hsp70-9d    -----MASV----------------------------ALLRALRRRELHTASVSAFKSVSTNGKTSLV-----------GH
BrHsp70-10a     -----MATA----------------------------ALLRSIRRRDVASTPFSAFKCLSRSAKTSSNSS---YHAQNWRS
BrHsp70-10b     -----MATA----------------------------ALLRSIRRRELASGPLSAYKCIQSSGKASLN-------GQNWRS
BoHsp70-10      -----MATA----------------------------ALLRSIRRRDVASAPFSAYKCIQSSGKASLN-------GQNWRS
BnA.Hsp70-10a   -----MATA----------------------------ALLRSIRRREVASTPFSAFKCLSRSAKTSSNSS---YHAQNWRS
BnA.Hsp70-10b   -----MATA----------------------------ALLRSIRRRELASAPLSAYKCIQSSGKASLN-------GQNWRS
BnC.Hsp70-10c   -----MATA----------------------------ALLRSIRRRDVASAPFSAYKCIQSSGKASLN-------GQNWRS
BnC.Hsp70-10d   -----MATA----------------------------ALLRSIRRREVASTPFSAFKCLWSTGKASSNGS---YHAQNWRS
```

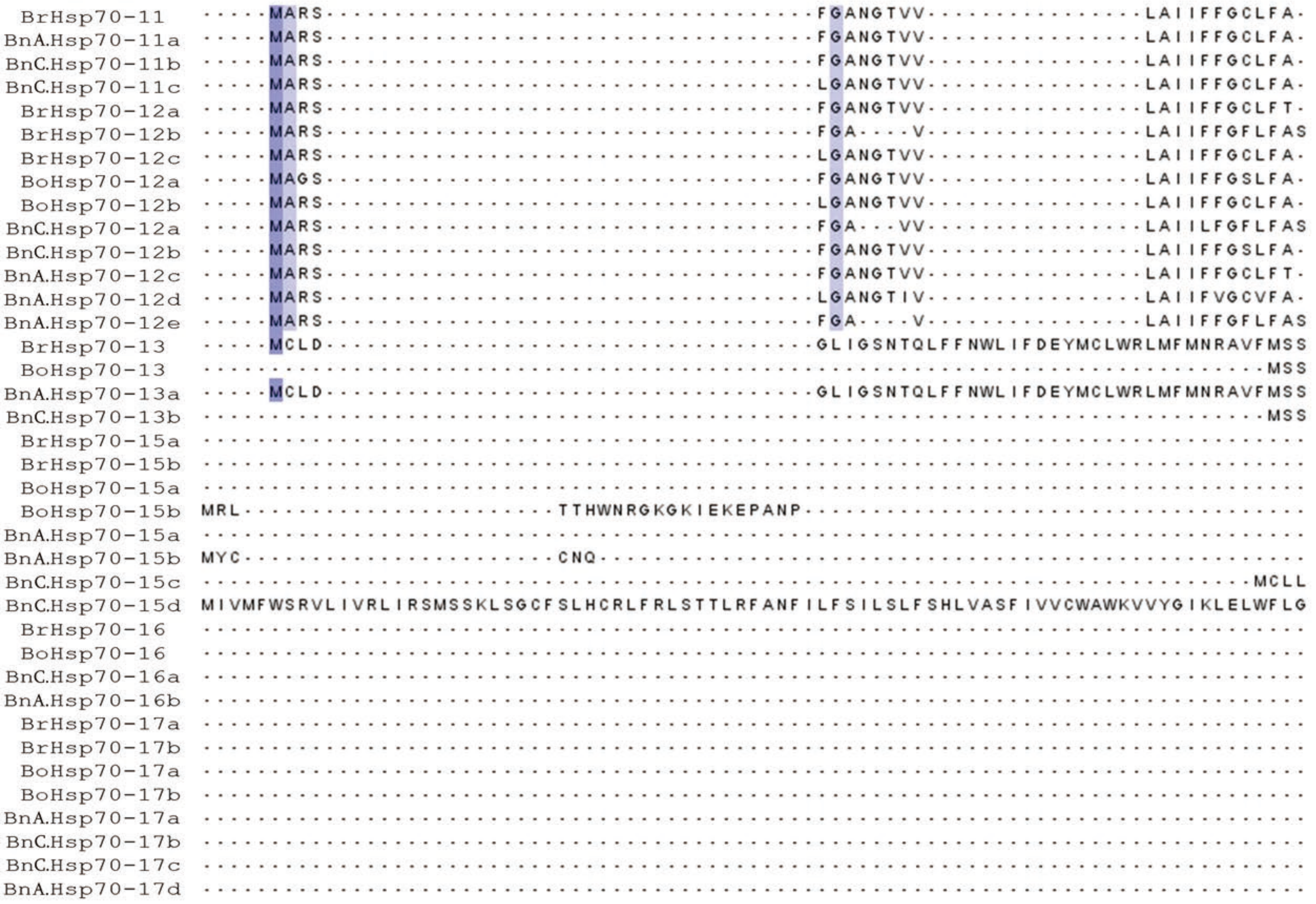
BrHsp70-11 MARS FGANGTVV LAIIFFGCLFA
BnA.Hsp70-11a MARS FGANGTVV LAIIFFGCLFA
BnC.Hsp70-11b MARS FGANGTVV LAIIFFGCLFA
BnC.Hsp70-11c MARS LGANGTVV LAIIFFGCLFA
BrHsp70-12a MARS FGANGTVV LAIIFFGCLFT
BrHsp70-12b MARS FGA V LAIIFFGFLFAS
BrHsp70-12c MARS LGANGTVV LAIIFFGCLFA
BoHsp70-12a MAGS FGANGTVV LAIIFFGSLFA
BoHsp70-12b MARS LGANGTVV LAIIFFGCLFA
BnC.Hsp70-12a MARS FGA VV LAIILFGFLFAS
BnC.Hsp70-12b MARS FGANGTVV LAIIFFGSLFA
BnA.Hsp70-12c MARS FGANGTVV LAIIFFGCLFT
BnA.Hsp70-12d MARS LGANGTIV LAIIFVGCVFA
BnA.Hsp70-12e MARS FGA V LAIIFFGFLFAS
BrHsp70-13 MCLD GLIGSNTQLFFNWLIFDEYMCLWRLMFMNRAVFMSS
BoHsp70-13 MSS
BnA.Hsp70-13a MCLD GLIGSNTQLFFNWLIFDEYMCLWRLMFMNRAVFMSS
BnC.Hsp70-13b MSS
BrHsp70-15a
BrHsp70-15b
BoHsp70-15a
BoHsp70-15b MRL TTHWNRGKGKIEKEPANP
BnA.Hsp70-15a
BnA.Hsp70-15b MYC CNQ
BnC.Hsp70-15c MCLL
BnC.Hsp70-15d MIVMFWSRVLIVRLIRSMSSKLSGCFSLHCRLFRLSTTLRFANFILFSILSLFSHLVASFIVVCWAWKVVYGIKLELWFLG
BrHsp70-16
BoHsp70-16
BnC.Hsp70-16a
BnA.Hsp70-16b
BrHsp70-17a
BrHsp70-17b
BoHsp70-17a
BoHsp70-17b
BnA.Hsp70-17a
BnC.Hsp70-17b
BnC.Hsp70-17c
BnA.Hsp70-17d

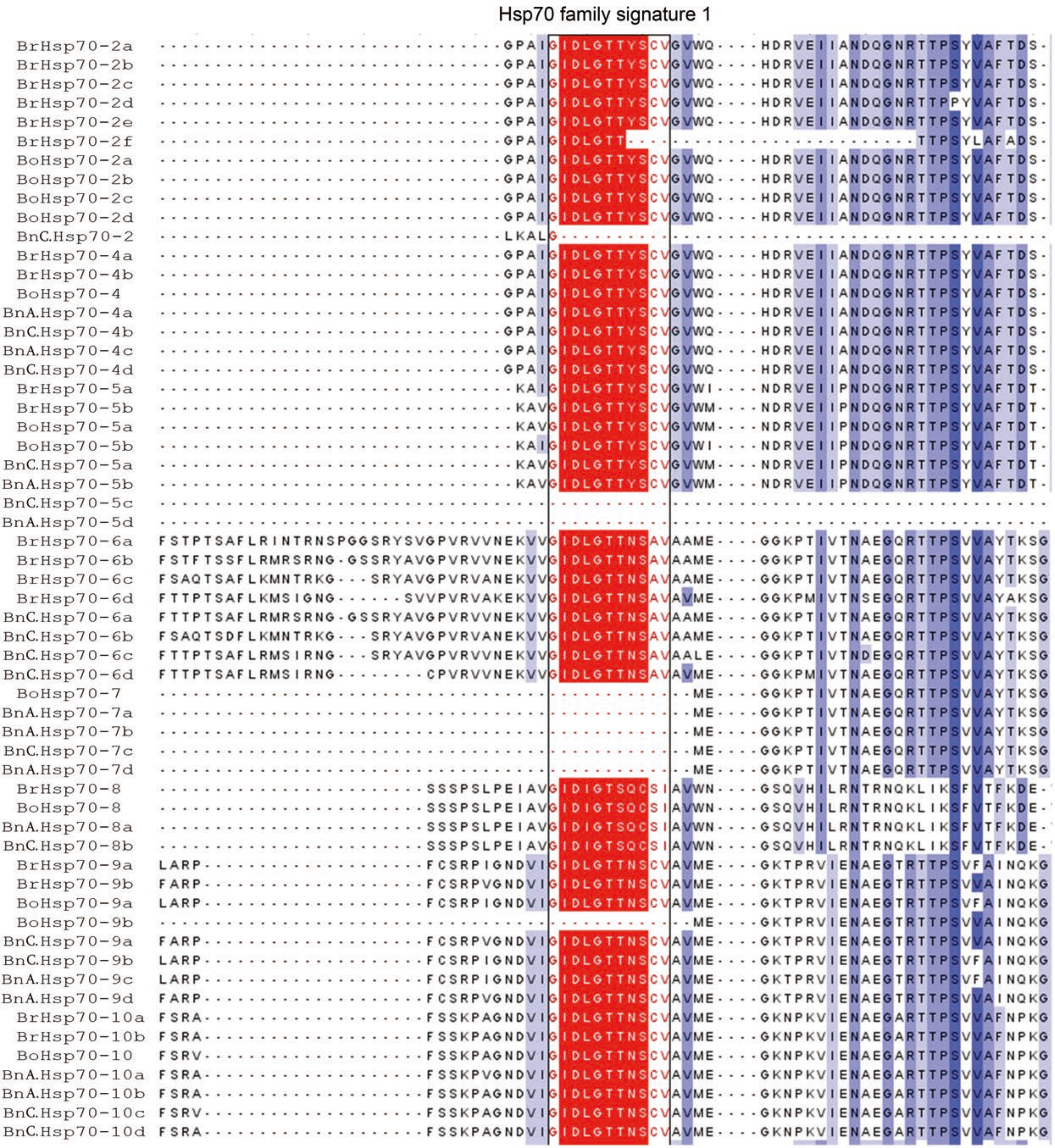
Hsp70 family signature 1
BrHsp70-2a
BrHsp70-2b
BrHsp70-2c
BrHsp70-2d
BrHsp70-2e
BrHsp70-2f
BoHsp70-2a
BoHsp70-2b
BoHsp70-2c
BoHsp70-2d
BnC.Hsp70-2
BrHsp70-4a
BrHsp70-4b
BoHsp70-4
BnA.Hsp70-4a
BnC.Hsp70-4b
BnA.Hsp70-4c
BnC.Hsp70-4d
BrHsp70-5a
BrHsp70-5b
BoHsp70-5a
BoHsp70-5b
BnC.Hsp70-5a
BnA.Hsp70-5b
BnC.Hsp70-5c
BnA.Hsp70-5d
BrHsp70-6a
BrHsp70-6b
BrHsp70-6c
BrHsp70-6d
BnC.Hsp70-6a
BnC.Hsp70-6b
BnC.Hsp70-6c
BnC.Hsp70-6d
BoHsp70-7
BnA.Hsp70-7a
BnA.Hsp70-7b
BnC.Hsp70-7c
BnA.Hsp70-7d
BrHsp70-8
BoHsp70-8
BnA.Hsp70-8a
BnC.Hsp70-8b
BrHsp70-9a
BrHsp70-9b
BoHsp70-9a
BoHsp70-9b
BnC.Hsp70-9a
BnC.Hsp70-9b
BnA.Hsp70-9c
BnA.Hsp70-9d
BrHsp70-10a
BrHsp70-10b
BoHsp70-10
BnA.Hsp70-10a
BnA.Hsp70-10b
BnC.Hsp70-10c
BnC.Hsp70-10d

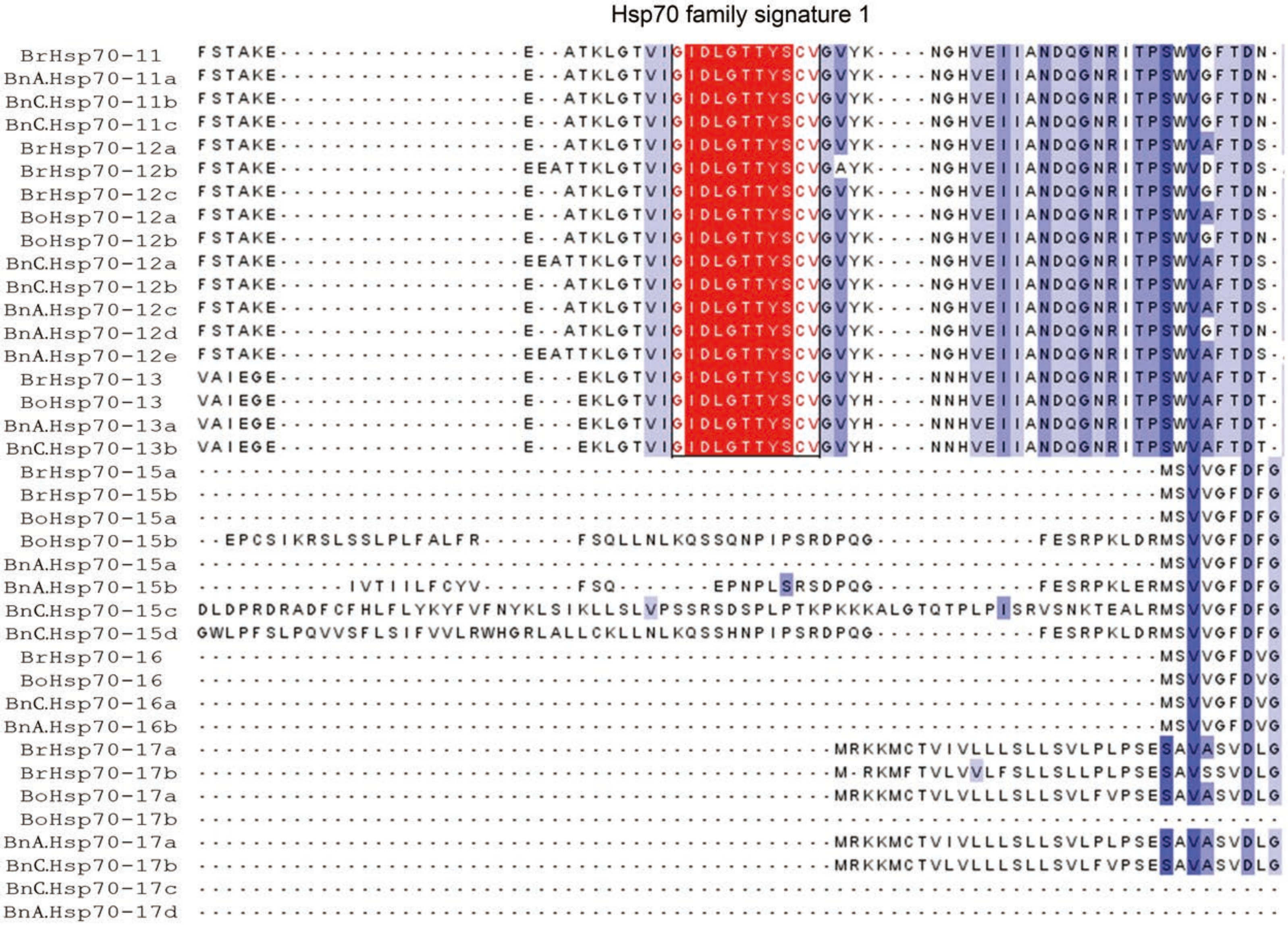
Hsp70 family signature 1
BrHsp70-11
BnA.Hsp70-11a
BnC.Hsp70-11b
BnC.Hsp70-11c
BrHsp70-12a
BrHsp70-12b
BrHsp70-12c
BoHsp70-12a
BoHsp70-12b
BnC.Hsp70-12a
BnC.Hsp70-12b
BnA.Hsp70-12c
BnA.Hsp70-12d
BnA.Hsp70-12e
BrHsp70-13
BoHsp70-13
BnA.Hsp70-13a
BnC.Hsp70-13b
BrHsp70-15a
BrHsp70-15b
BoHsp70-15a
BoHsp70-15b
BnA.Hsp70-15a
BnA.Hsp70-15b
BnC.Hsp70-15c
BnC.Hsp70-15d
BrHsp70-16
BoHsp70-16
BnC.Hsp70-16a
BnA.Hsp70-16b
BrHsp70-17a
BrHsp70-17b
BoHsp70-17a
BoHsp70-17b
BnA.Hsp70-17a
BnC.Hsp70-17b
BnC.Hsp70-17c
BnA.Hsp70-17d

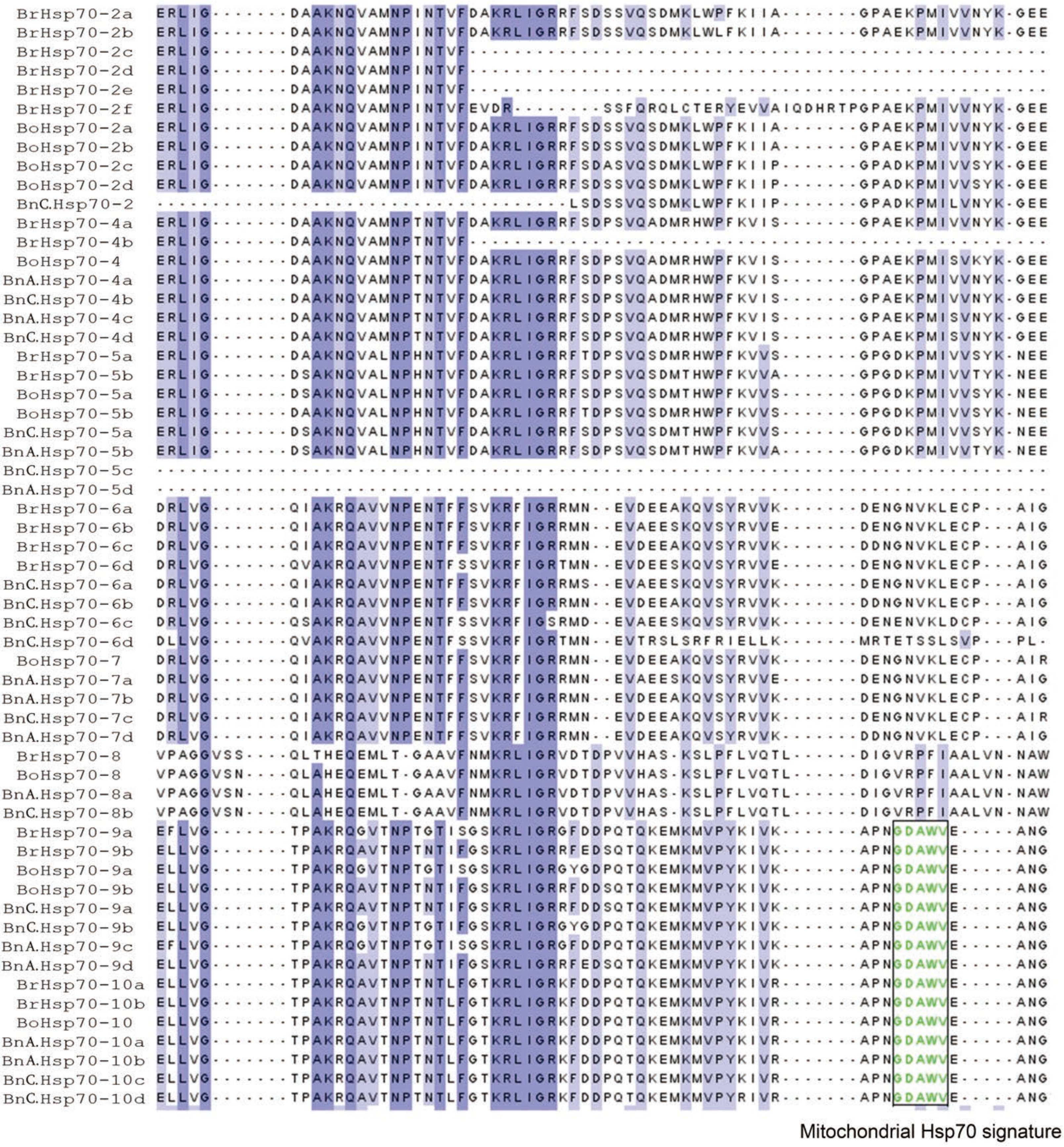
BrHsp70-2a
BrHsp70-2b
BrHsp70-2c
BrHsp70-2d
BrHsp70-2e
BrHsp70-2f
BoHsp70-2a
BoHsp70-2b
BoHsp70-2c
BoHsp70-2d
BnC.Hsp70-2
BrHsp70-4a
BrHsp70-4b
BoHsp70-4
BnA.Hsp70-4a
BnC.Hsp70-4b
BnA.Hsp70-4c
BnC.Hsp70-4d
BrHsp70-5a
BrHsp70-5b
BoHsp70-5a
BoHsp70-5b
BnC.Hsp70-5a
BnA.Hsp70-5b
BnC.Hsp70-5c
BnA.Hsp70-5d
BrHsp70-6a
BrHsp70-6b
BrHsp70-6c
BrHsp70-6d
BnC.Hsp70-6a
BnC.Hsp70-6b
BnC.Hsp70-6c
BnC.Hsp70-6d
BoHsp70-7
BnA.Hsp70-7a
BnA.Hsp70-7b
BnC.Hsp70-7c
BnA.Hsp70-7d
BrHsp70-8
BoHsp70-8
BnA.Hsp70-8a
BnC.Hsp70-8b
BrHsp70-9a
BrHsp70-9b
BoHsp70-9a
BoHsp70-9b
BnC.Hsp70-9a
BnC.Hsp70-9b
BnA.Hsp70-9c
BnA.Hsp70-9d
BrHsp70-10a
BrHsp70-10b
BoHsp70-10
BnA.Hsp70-10a
BnA.Hsp70-10b
BnC.Hsp70-10c
BnC.Hsp70-10d
Mitochondrial Hsp70 signature

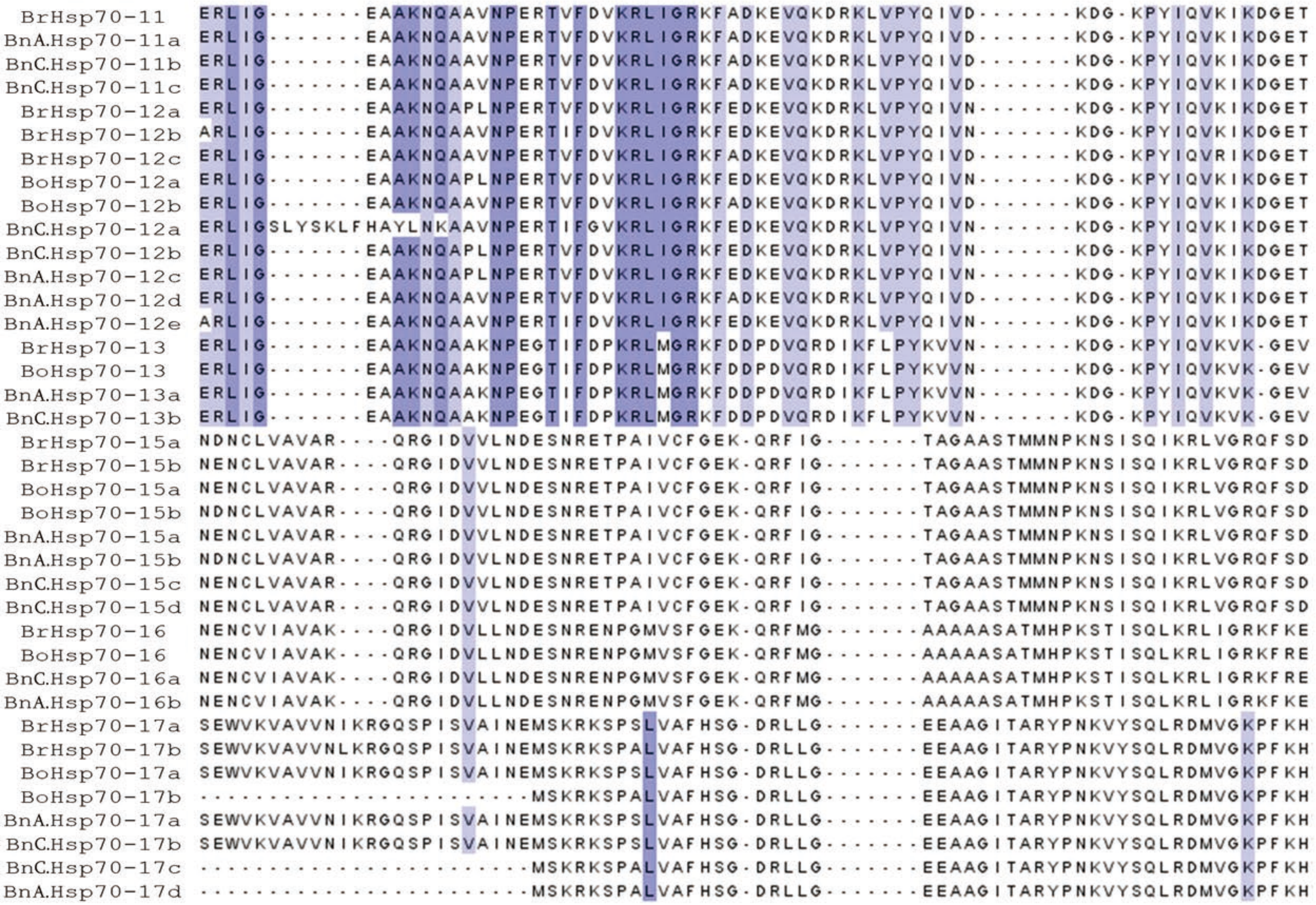
BrHsp70-11 ERLIG-------EAAKNQAAVNPERTVFDVKRLIGRKFADKEVQKDRKLVPYQIVD-------KDG-KPYIQVKIKDGET
BnA.Hsp70-11a ERLIG-------EAAKNQAAVNPERTVFDVKRLIGRKFADKEVQKDRKLVPYQIVD-------KDG-KPYIQVKIKDGET
BnC.Hsp70-11b ERLIG-------EAAKNQAAVNPERTVFDVKRLIGRKFADKEVQKDRKLVPYQIVD-------KDG-KPYIQVKIKDGET
BnC.Hsp70-11c ERLIG-------EAAKNQAAVNPERTVFDVKRLIGRKFADKEVQKDRKLVPYQIVD-------KDG-KPYIQVKIKDGET
BrHsp70-12a ERLIG-------EAAKNQAPLNPERTVFDVKRLIGRKFEDKEVQKDRKLVPYQIVN-------KDG-KPYIQVKIKDGET
BrHsp70-12b ARLIG-------EAAKNQAAVNPERTIFDVKRLIGRKFEDKEVQKDRKLVPYQIVN-------KDG-KPYIQVKIKDGET
BrHsp70-12c ERLIG-------EAAKNQAAVNPERTVFDVKRLIGRKFADKEVQKDRKLVPYQIVD-------KDG-KPYIQVRIKDGET
BoHsp70-12a ERLIG-------EAAKNQAPLNPERTVFDVKRLIGRKFEDKEVQKDRKLVPYQIVN-------KDG-KPYIQVKIKDGET
BoHsp70-12b ERLIG-------EAAKNQAAVNPERTVFDVKRLIGRKFADKEVQKDRKLVPYQIVD-------KDG-KPYIQVKIKDGET
BnC.Hsp70-12a ERLIGSLYSKLFHAYLNKAAVNPERTIFGVKRLIGRKFEDKEVQKDRKLVPYQIVN-------KDG-KPYIQVKIKDGET
BnC.Hsp70-12b ERLIG-------EAAKNQAPLNPERTVFDVKRLIGRKFEDKEVQKDRKLVPYQIVN-------KDG-KPYIQVKIKDGET
BnA.Hsp70-12c ERLIG-------EAAKNQAPLNPERTVFDVKRLIGRKFEDKEVQKDRKLVPYQIVN-------KDG-KPYIQVKIKDGET
BnA.Hsp70-12d ERLIG-------EAAKNQAAVNPERTVFDVKRLIGRKFADKEVQKDRKLVPYQIVD-------KDG-KPYIQVKIKDGET
BnA.Hsp70-12e ARLIG-------EAAKNQAAVNPERTIFDVKRLIGRKFEDKEVQKDRKLVPYQIVN-------KDG-KPYIQVKIKDGET
BrHsp70-13 ERLIG-------EAAKNQAAKNPEGTIFDPKRLMGRKFDDPDVQRDIKFLPYKVVN-------KDG-KPYIQVKVK-GEV
BoHsp70-13 ERLIG-------EAAKNQAAKNPEGTIFDPKRLMGRKFDDPDVQRDIKFLPYKVVN-------KDG-KPYIQVKVK-GEV
BnA.Hsp70-13a ERLIG-------EAAKNQAAKNPEGTIFDPKRLMGRKFDDPDVQRDIKFLPYKVVN-------KDG-KPYIQVKVK-GEV
BnC.Hsp70-13b ERLIG-------EAAKNQAAKNPEGTIFDPKRLMGRKFDDPDVQRDIKFLPYKVVN-------KDG-KPYIQVKVK-GEV
BrHsp70-15a NDNCLVAVAR----QRGIDVVLNDESNRETPAIVCFGEK-QRFIG-------TAGAASTMMNPKNSISQIKRLVGRQFSD
BrHsp70-15b NENCLVAVAR----QRGIDVVLNDESNRETPAIVCFGEK-QRFIG-------TAGAASTMMNPKNSISQIKRLVGRQFSD
BoHsp70-15a NENCLVAVAR----QRGIDVVLNDESNRETPAIVCFGEK-QRFIG-------TAGAASTMMNPKNSISQIKRLVGRQFSD
BoHsp70-15b NDNCLVAVAR----QRGIDVVLNDESNRETPAIVCFGEK-QRFIG-------TAGAASTMMNPKNSISQIKRLVGRQFSD
BnA.Hsp70-15a NENCLVAVAR----QRGIDVVLNDESNRETPAIVCFGEK-QRFIG-------TAGAASTMMNPKNSISQIKRLVGRQFSD
BnA.Hsp70-15b NDNCLVAVAR----QRGIDVVLNDESNRETPAIVCFGEK-QRFIG-------TAGAASTMMNPKNSISQIKRLVGRQFSD
BnC.Hsp70-15c NENCLVAVAR----QRGIDVVLNDESNRETPAIVCFGEK-QRFIG-------TAGAASTMMNPKNSISQIKRLVGRQFSD
BnC.Hsp70-15d NENCLVAVAR----QRGIDVVLNDESNRETPAIVCFGEK-QRFIG-------TAGAASTMMNPKNSISQIKRLVGRQFSD
BrHsp70-16 NENCVIAVAK----QRGIDVLLNDESNRENPGMVSFGEK-QRFMG-------AAAAASATMHPKSTISQLKRLIGRKFKE
BoHsp70-16 NENCVIAVAK----QRGIDVLLNDESNRENPGMVSFGEK-QRFMG-------AAAAASATMHPKSTISQLKRLIGRKFRE
BnC.Hsp70-16a NENCVIAVAK----QRGIDVLLNDESNRENPGMVSFGEK-QRFMG-------AAAAASATMHPKSTISQLKRLIGRKFRE
BnA.Hsp70-16b NENCVIAVAK----QRGIDVLLNDESNRENPGMVSFGEK-QRFMG-------AAAAASATMHPKSTISQLKRLIGRKFKE
BrHsp70-17a SEWVKVAVVNIKRGQSPISVAINEMSKRKSPSLVAFHSG-DRLLG-------EEAAGITARYPNKVYSQLRDMVGKPFKH
BrHsp70-17b SEWVKVAVVNLKRGQSPISVAINEMSKRKSPALVAFHSG-DRLLG-------EEAAGITARYPNKVYSQLRDMVGKPFKH
BoHsp70-17a SEWVKVAVVNIKRGQSPISVAINEMSKRKSPSLVAFHSG-DRLLG-------EEAAGITARYPNKVYSQLRDMVGKPFKH
BoHsp70-17b ------------------------MSKRKSPALVAFHSG-DRLLG-------EEAAGITARYPNKVYSQLRDMVGKPFKH
BnA.Hsp70-17a SEWVKVAVVNIKRGQSPISVAINEMSKRKSPSLVAFHSG-DRLLG-------EEAAGITARYPNKVYSQLRDMVGKPFKH
BnC.Hsp70-17b SEWVKVAVVNIKRGQSPISVAINEMSKRKSPSLVAFHSG-DRLLG-------EEAAGITARYPNKVYSQLRDMVGKPFKH
BnC.Hsp70-17c ------------------------MSKRKSPALVAFHSG-DRLLG-------EEAAGITARYPNKVYSQLRDMVGKPFKH
BnA.Hsp70-17d ------------------------MSKRKSPALVAFHSG-DRLLG-------EEAAGITARYPNKVYSQLRDMVGKPFKH

```
BrHsp70-2a    ................................................................................
BrHsp70-2b    ................................................................................
BrHsp70-2c    ................................................................................
BrHsp70-2d    ................................................................................
BrHsp70-2e    ................................................................................
BrHsp70-2f    ................................................................................
BoHsp70-2a    ................................................................................
BoHsp70-2b    ................................................................................
BoHsp70-2c    ................................................................................
BoHsp70-2d    ................................................................................
BnC.Hsp70-2   ................................................................................
BrHsp70-4a    ................................................................................
BrHsp70-4b    ................................................................................
BoHsp70-4     ................................................................................
BnA.Hsp70-4a  ................................................................................
BnC.Hsp70-4b  ................................................................................
BnA.Hsp70-4c  ................................................................................
BnC.Hsp70-4d  ................................................................................
BrHsp70-5a    ................................................................................
BrHsp70-5b    ................................................................................
BoHsp70-5a    ................................................................................
BoHsp70-5b    ................................................................................
BnC.Hsp70-5a  ................................................................................
BnA.Hsp70-5b  ................................................................................
BnC.Hsp70-5c  ................................................................................
BnA.Hsp70-5d  ................................................................................
BrHsp70-6a    ................................................................................
BrHsp70-6b    ................................................................................
BrHsp70-6c    ................................................................................
BrHsp70-6d    ................................................................................
BnC.Hsp70-6a  ................................................................................
BnC.Hsp70-6b  ................................................................................
BnC.Hsp70-6c  ................................................................................
BnC.Hsp70-6d  ................................................................................
BoHsp70-7     ................................................................................
BnA.Hsp70-7a  ................................................................................
BnA.Hsp70-7b  ................................................................................
BnC.Hsp70-7c  ................................................................................
BnA.Hsp70-7d  ................................................................................
BrHsp70-8     ................................................................................
BoHsp70-8     ................................................................................
BnA.Hsp70-8a  ................................................................................
BnC.Hsp70-8b  ................................................................................
BrHsp70-9a    ................................................................................
BrHsp70-9b    ................................................................................
BoHsp70-9a    ................................................................................
BoHsp70-9b    ................................................................................
BnC.Hsp70-9a  ................................................................................
BnC.Hsp70-9b  ................................................................................
BnA.Hsp70-9c  ................................................................................
BnA.Hsp70-9d  ................................................................................
BrHsp70-10a   ................................................................................
BrHsp70-10b   ................................................................................
BoHsp70-10    ................................................................................
BnA.Hsp70-10a ................................................................................
BnA.Hsp70-10b ................................................................................
BnC.Hsp70-10c ................................................................................
BnC.Hsp70-10d VFPWRFIRVVSRDKVWFLGICGFVGGCGWGVFVERALIWIEGSSGFLWYVGQGRFMEGSYLSSACYRVALPVRCIAANRT
```

```
BrHsp70-11      ---------------------------------------------------------------------------------
BnA.Hsp70-11a   ---------------------------------------------------------------------------------
BnC.Hsp70-11b   ---------------------------------------------------------------------------------
BnC.Hsp70-11c   ---------------------------------------------------------------------------------
BrHsp70-12a     ---------------------------------------------------------------------------------
BrHsp70-12b     ---------------------------------------------------------------------------------
BrHsp70-12c     ---------------------------------------------------------------------------------
BoHsp70-12a     ---------------------------------------------------------------------------------
BoHsp70-12b     ---------------------------------------------------------------------------------
BnC.Hsp70-12a   ---------------------------------------------------------------------------------
BnC.Hsp70-12b   ---------------------------------------------------------------------------------
BnA.Hsp70-12c   ---------------------------------------------------------------------------------
BnA.Hsp70-12d   ---------------------------------------------------------------------------------
BnA.Hsp70-12e   ---------------------------------------------------------------------------------
BrHsp70-13      ---------------------------------------------------------------------------------
BoHsp70-13      ---------------------------------------------------------------------------------
BnA.Hsp70-13a   ---------------------------------------------------------------------------------
BnC.Hsp70-13b   ---------------------------------------------------------------------------------
BrHsp70-15a     PELQRDIKSLPFSVTE-------GPDGYPLIHASYL-GEK-----------------------------------------
BrHsp70-15b     PDLQRDIKSLPFSVTE-------GPDGYPLIHASYL-GEK-----------------------------------------
BoHsp70-15a     PELQRDIKSLPFSVTE-------GPDGYPLIHASYL-GEK-----------------------------------------
BoHsp70-15b     PELQRDIKSLPFSVTE-------GPDGYPLIHASYL-GEK-----------------------------------------
BnA.Hsp70-15a   PELQRDIKSLPFSVTE-------GPDGYPLIHASYL-GEK-----------------------------------------
BnA.Hsp70-15b   PELQRDIKSLPFSVTE-------GPDGYPLIHASYL-GEK-----------------------------------------
BnC.Hsp70-15c   PELQRDIKSLPFSVTE-------GPDGYPLIHASYL-GEK-----------------------------------------
BnC.Hsp70-15d   PELQRDIKSLPFSVTE-------GPDGYPLIHASYL-GEK-----------------------------------------
BrHsp70-16      PDVQKDLKLFPFETSED------SADGGIQIQLRYM-GEV-----------------------------------------
BoHsp70-16      PDVQKDLKLFPFETSED------SADGGIQIRLRYM-GEV-----------------------------------------
BnC.Hsp70-16a   PDVQKDLKLFPFETSED------SADGGIQIRLRYM-GEV-----------------------------------------
BnA.Hsp70-16b   PDVQKDLKLFPFETSED------SADGGIQIQLRYM-GEV-----------------------------------------
BrHsp70-17a     VKDFIDSVYLPFDIVE-------DSRGAVGVKID---DGT-----------------------------------------
BrHsp70-17b     VKDFIDSVYLPFDIVE-------DSRGAVGIKID---DGA-----------------------------------------
BoHsp70-17a     VKDFIDSVYLPFDIVE-------DSRGAVGVKID---DGT-----------------------------------------
BoHsp70-17b     VKDFIDSVYLPFGIVE-------DSRGAVGIKID---DGA-----------------------------------------
BnA.Hsp70-17a   VKDFIDSVYLPFDIVE-------DSRGAVGVKID---DGT-----------------------------------------
BnC.Hsp70-17b   VKDFIDSVYLPFDIVE-------DSRGAVGVKID---DGT-----------------------------------------
BnC.Hsp70-17c   VKDFIDSVYLPFDIVE-------DSRGAVGIKID---DGA-----------------------------------------
BnA.Hsp70-17d   VKDFIDSVYLPFDIVE-------DSRGAVGIKID---DGA-----------------------------------------
```

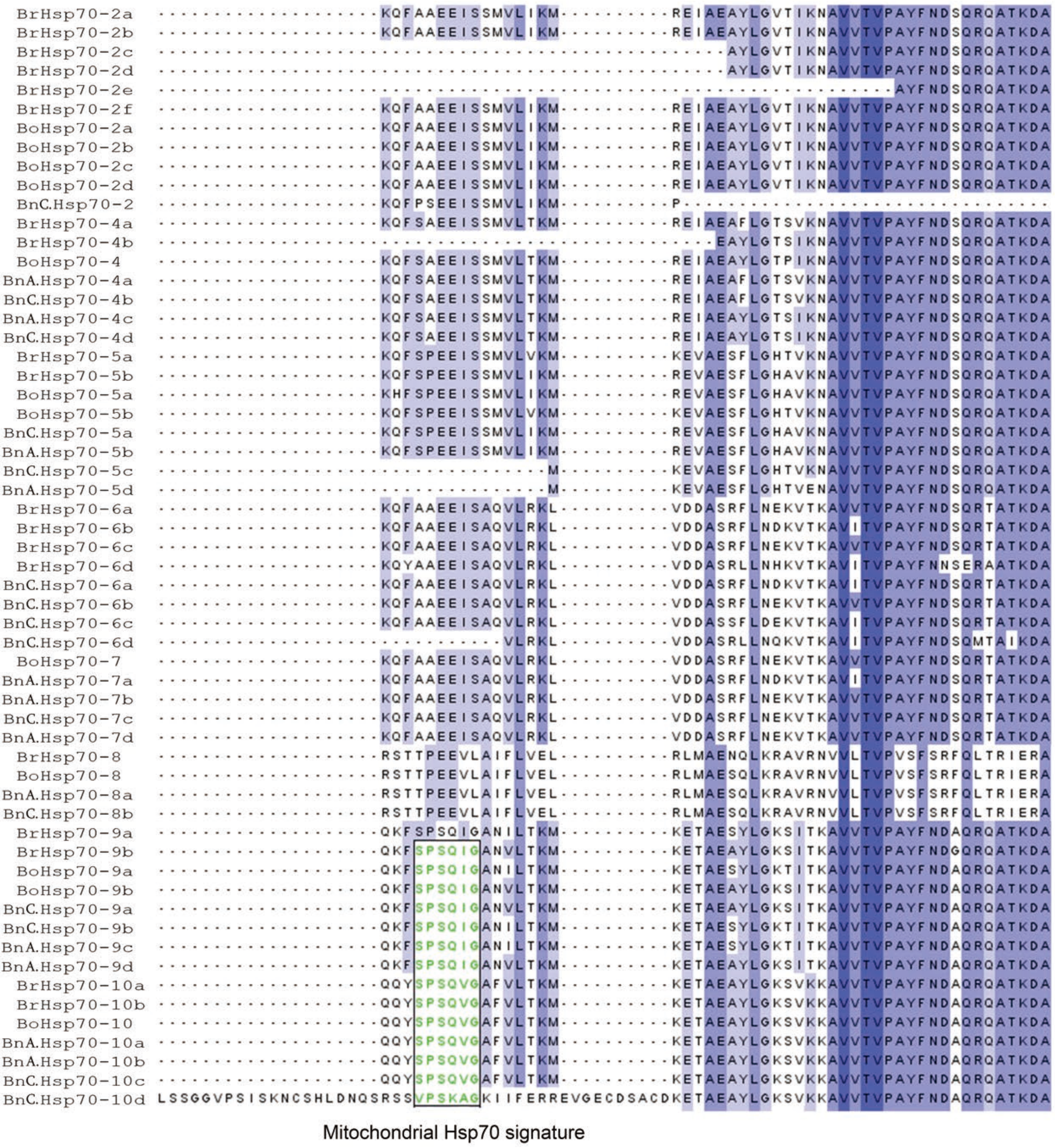
BrHsp70-2a
BrHsp70-2b
BrHsp70-2c
BrHsp70-2d
BrHsp70-2e
BrHsp70-2f
BoHsp70-2a
BoHsp70-2b
BoHsp70-2c
BoHsp70-2d
BnC.Hsp70-2
BrHsp70-4a
BrHsp70-4b
BoHsp70-4
BnA.Hsp70-4a
BnC.Hsp70-4b
BnA.Hsp70-4c
BnC.Hsp70-4d
BrHsp70-5a
BrHsp70-5b
BoHsp70-5a
BoHsp70-5b
BnC.Hsp70-5a
BnA.Hsp70-5b
BnC.Hsp70-5c
BnA.Hsp70-5d
BrHsp70-6a
BrHsp70-6b
BrHsp70-6c
BrHsp70-6d
BnC.Hsp70-6a
BnC.Hsp70-6b
BnC.Hsp70-6c
BnC.Hsp70-6d
BoHsp70-7
BnA.Hsp70-7a
BnA.Hsp70-7b
BnC.Hsp70-7c
BnA.Hsp70-7d
BrHsp70-8
BoHsp70-8
BnA.Hsp70-8a
BnC.Hsp70-8b
BrHsp70-9a
BrHsp70-9b
BoHsp70-9a
BoHsp70-9b
BnC.Hsp70-9a
BnC.Hsp70-9b
BnA.Hsp70-9c
BnA.Hsp70-9d
BrHsp70-10a
BrHsp70-10b
BoHsp70-10
BnA.Hsp70-10a
BnA.Hsp70-10b
BnC.Hsp70-10c
BnC.Hsp70-10d
Mitochondrial Hsp70 signature

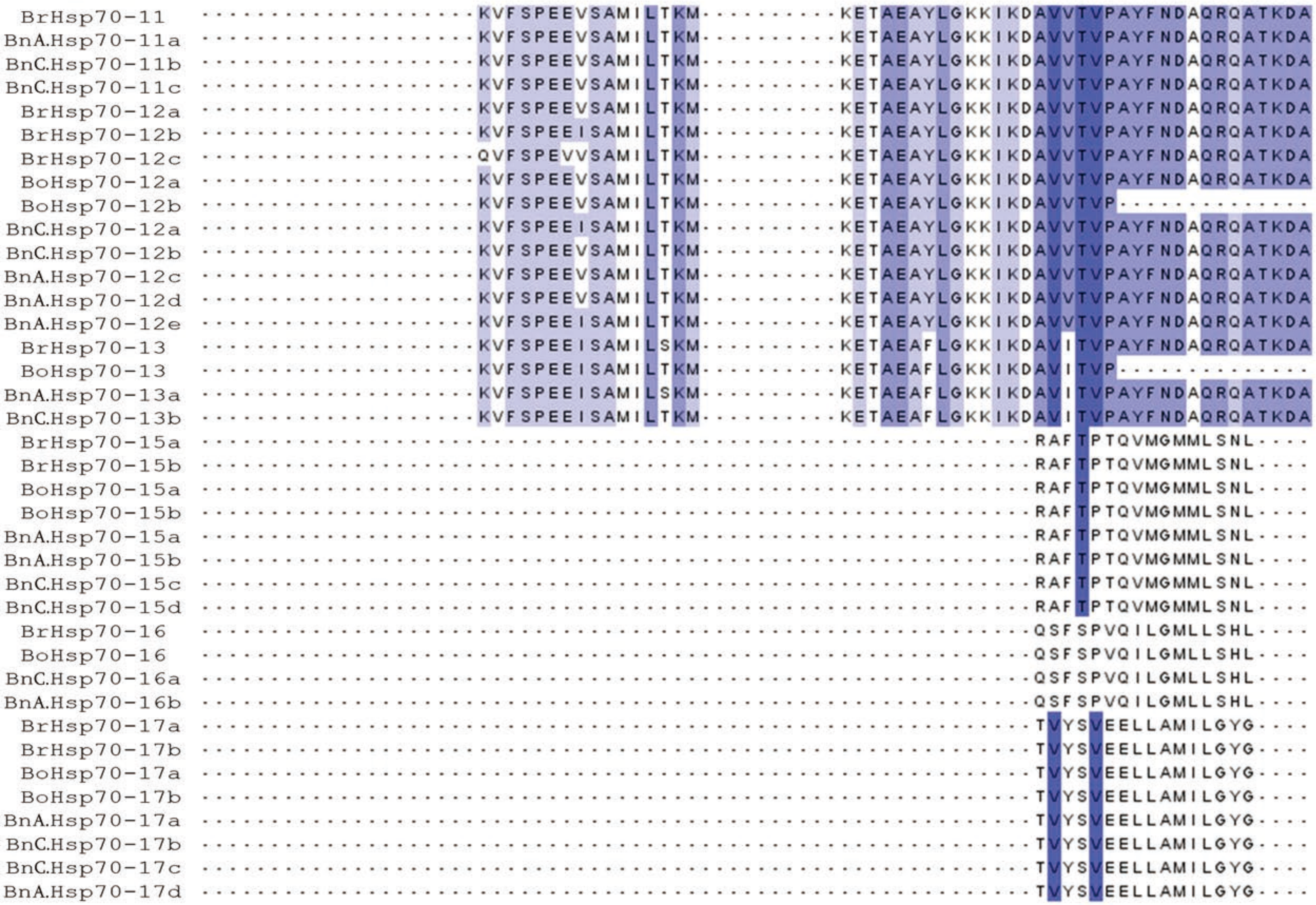

BrHsp70-11 KVFSPEEVSAMILTKM KETAEAYLGKKIKDAVVTVPAYFNDAQRQATKDA
BnA.Hsp70-11a KVFSPEEVSAMILTKM KETAEAYLGKKIKDAVVTVPAYFNDAQRQATKDA
BnC.Hsp70-11b KVFSPEEVSAMILTKM KETAEAYLGKKIKDAVVTVPAYFNDAQRQATKDA
BnC.Hsp70-11c KVFSPEEVSAMILTKM KETAEAYLGKKIKDAVVTVPAYFNDAQRQATKDA
BrHsp70-12a KVFSPEEVSAMILTKM KETAEAYLGKKIKDAVVTVPAYFNDAQRQATKDA
BrHsp70-12b KVFSPEEISAMILTKM KETAEAYLGKKIKDAVVTVPAYFNDAQRQATKDA
BrHsp70-12c QVFSPEVVSAMILTKM KETAEAYLGKKIKDAVVTVPAYFNDAQRQATKDA
BoHsp70-12a KVFSPEEVSAMILTKM KETAEAYLGKKIKDAVVTVPAYFNDAQRQATKDA
BoHsp70-12b KVFSPEEVSAMILTKM KETAEAYLGKKIKDAVVTVP
BnC.Hsp70-12a KVFSPEEISAMILTKM KETAEAYLGKKIKDAVVTVPAYFNDAQRQATKDA
BnC.Hsp70-12b KVFSPEEVSAMILTKM KETAEAYLGKKIKDAVVTVPAYFNDAQRQATKDA
BnA.Hsp70-12c KVFSPEEVSAMILTKM KETAEAYLGKKIKDAVVTVPAYFNDAQRQATKDA
BnA.Hsp70-12d KVFSPEEVSAMILTKM KETAEAYLGKKIKDAVVTVPAYFNDAQRQATKDA
BnA.Hsp70-12e KVFSPEEISAMILTKM KETAEAYLGKKIKDAVVTVPAYFNDAQRQATKDA
BrHsp70-13 KVFSPEEISAMILSKM KETAEAFLGKKIKDAVITVPAYFNDAQRQATKDA
BoHsp70-13 KVFSPEEISAMILTKM KETAEAFLGKKIKDAVITVP
BnA.Hsp70-13a KVFSPEEISAMILSKM KETAEAFLGKKIKDAVITVPAYFNDAQRQATKDA
BnC.Hsp70-13b KVFSPEEISAMILTKM KETAEAFLGKKIKDAVITVPAYFNDAQRQATKDA
BrHsp70-15a RAFTPTQVMGMMLSNL
BrHsp70-15b RAFTPTQVMGMMLSNL
BoHsp70-15a RAFTPTQVMGMMLSNL
BoHsp70-15b RAFTPTQVMGMMLSNL
BnA.Hsp70-15a RAFTPTQVMGMMLSNL
BnA.Hsp70-15b RAFTPTQVMGMMLSNL
BnC.Hsp70-15c RAFTPTQVMGMMLSNL
BnC.Hsp70-15d RAFTPTQVMGMMLSNL
BrHsp70-16 QSFSPVQILGMLLSHL
BoHsp70-16 QSFSPVQILGMLLSHL
BnC.Hsp70-16a QSFSPVQILGMLLSHL
BnA.Hsp70-16b QSFSPVQILGMLLSHL
BrHsp70-17a TVYSVEELLAMILGYG
BrHsp70-17b TVYSVEELLAMILGYG
BoHsp70-17a TVYSVEELLAMILGYG
BoHsp70-17b TVYSVEELLAMILGYG
BnA.Hsp70-17a TVYSVEELLAMILGYG
BnC.Hsp70-17b TVYSVEELLAMILGYG
BnC.Hsp70-17c TVYSVEELLAMILGYG
BnA.Hsp70-17d TVYSVEELLAMILGYG

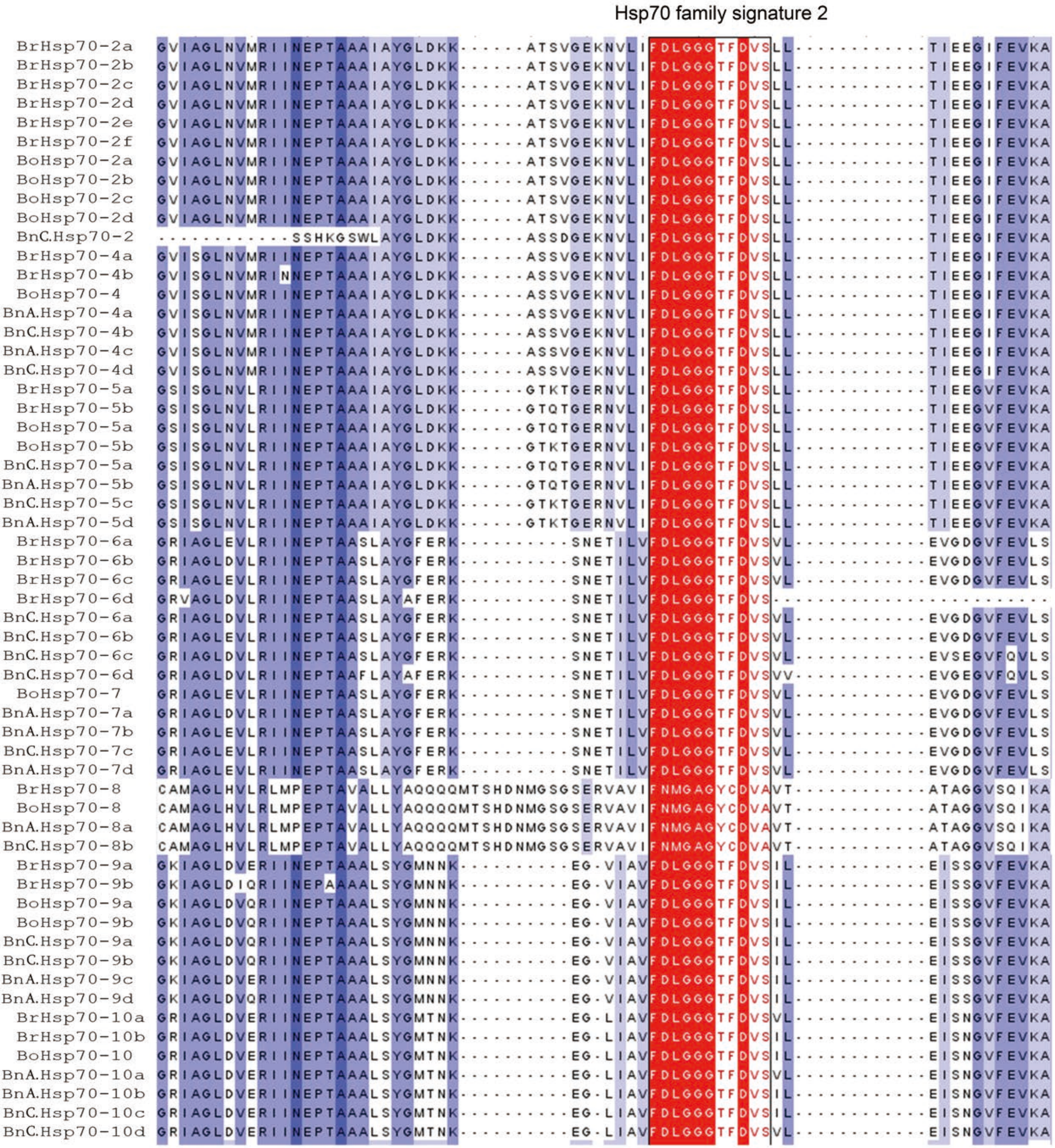
Hsp70 family signature 2
BrHsp70-2a
BrHsp70-2b
BrHsp70-2c
BrHsp70-2d
BrHsp70-2e
BrHsp70-2f
BoHsp70-2a
BoHsp70-2b
BoHsp70-2c
BoHsp70-2d
BnC.Hsp70-2
BrHsp70-4a
BrHsp70-4b
BoHsp70-4
BnA.Hsp70-4a
BnC.Hsp70-4b
BnA.Hsp70-4c
BnC.Hsp70-4d
BrHsp70-5a
BrHsp70-5b
BoHsp70-5a
BoHsp70-5b
BnC.Hsp70-5a
BnA.Hsp70-5b
BnC.Hsp70-5c
BnA.Hsp70-5d
BrHsp70-6a
BrHsp70-6b
BrHsp70-6c
BrHsp70-6d
BnC.Hsp70-6a
BnC.Hsp70-6b
BnC.Hsp70-6c
BnC.Hsp70-6d
BoHsp70-7
BnA.Hsp70-7a
BnA.Hsp70-7b
BnC.Hsp70-7c
BnA.Hsp70-7d
BrHsp70-8
BoHsp70-8
BnA.Hsp70-8a
BnC.Hsp70-8b
BrHsp70-9a
BrHsp70-9b
BoHsp70-9a
BoHsp70-9b
BnC.Hsp70-9a
BnC.Hsp70-9b
BnA.Hsp70-9c
BnA.Hsp70-9d
BrHsp70-10a
BrHsp70-10b
BoHsp70-10
BnA.Hsp70-10a
BnA.Hsp70-10b
BnC.Hsp70-10c
BnC.Hsp70-10d

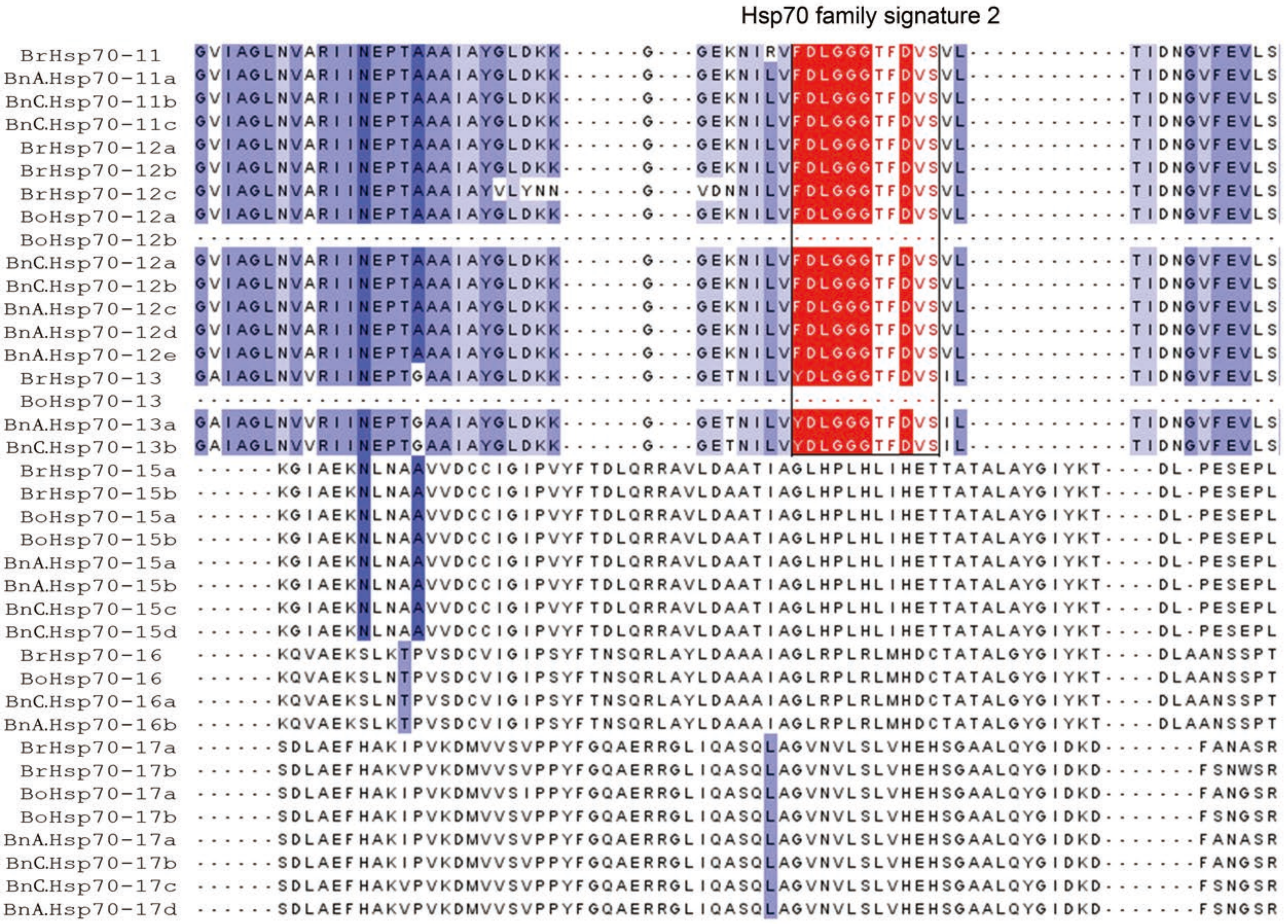
Hsp70 family signature 2
BrHsp70-11
BnA.Hsp70-11a
BnC.Hsp70-11b
BnC.Hsp70-11c
BrHsp70-12a
BrHsp70-12b
BrHsp70-12c
BoHsp70-12a
BoHsp70-12b
BnC.Hsp70-12a
BnC.Hsp70-12b
BnA.Hsp70-12c
BnA.Hsp70-12d
BnA.Hsp70-12e
BrHsp70-13
BoHsp70-13
BnA.Hsp70-13a
BnC.Hsp70-13b
BrHsp70-15a
BrHsp70-15b
BoHsp70-15a
BoHsp70-15b
BnA.Hsp70-15a
BnA.Hsp70-15b
BnC.Hsp70-15c
BnC.Hsp70-15d
BrHsp70-16
BoHsp70-16
BnC.Hsp70-16a
BnA.Hsp70-16b
BrHsp70-17a
BrHsp70-17b
BoHsp70-17a
BoHsp70-17b
BnA.Hsp70-17a
BnC.Hsp70-17b
BnC.Hsp70-17c
BnA.Hsp70-17d

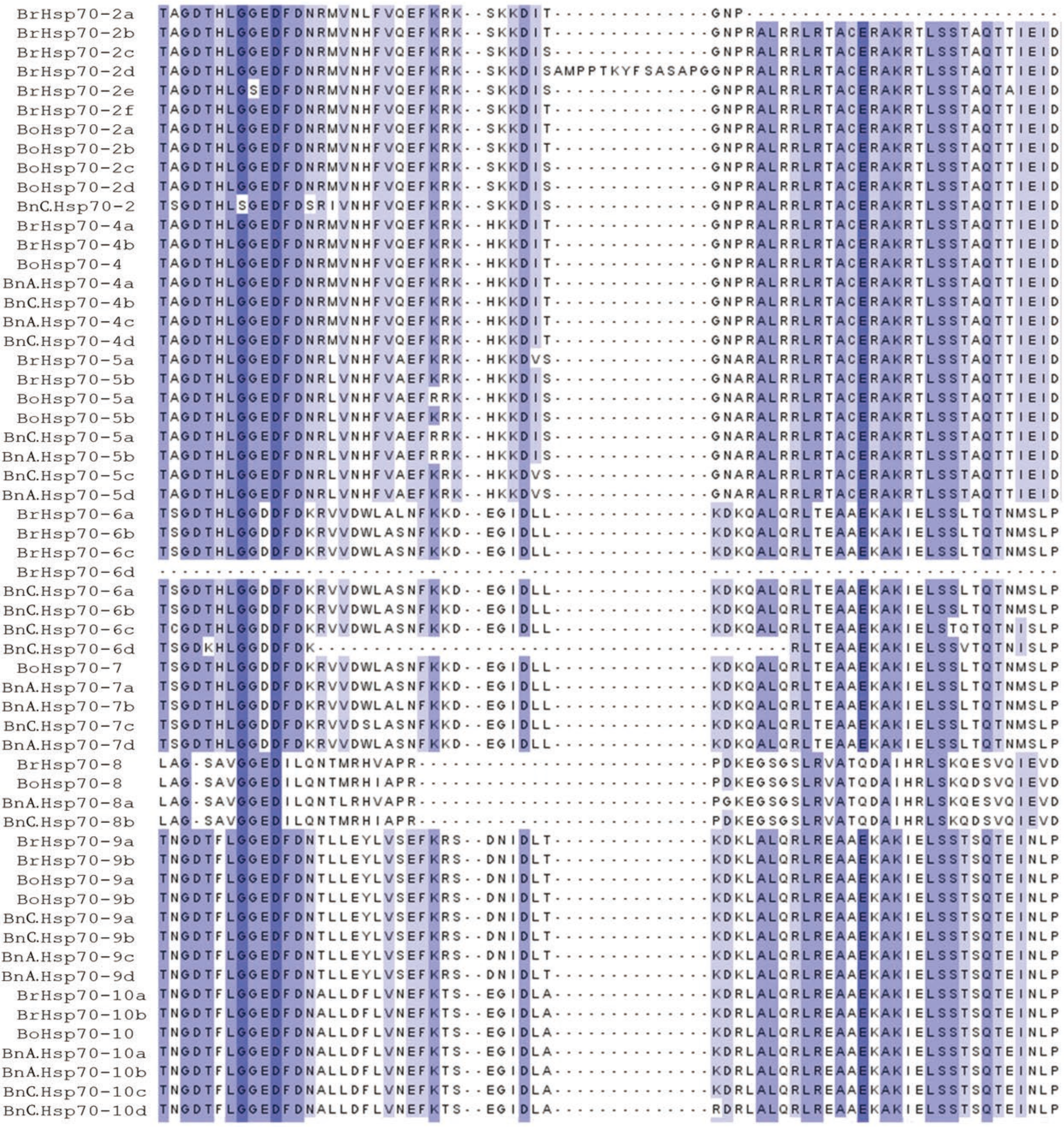

BrHsp70-2a TAGDTHLGGEDFDNRMVNLFVQEFKRK--SKKDIT--------------GNP----------------------------
BrHsp70-2b TAGDTHLGGEDFDNRMVNHFVQEFKRK--SKKDIT--------------GNPRALRRLRTACERAKRTLSSTAQTTIEID
BrHsp70-2c TAGDTHLGGEDFDNRMVNHFVQEFKRK--SKKDIS--------------GNPRALRRLRTACERAKRTLSSTAQTTIEID
BrHsp70-2d TAGDTHLGGEDFDNRMVNHFVQEFKRK--SKKDISAMPPTKYFSASAPGGNPRALRRLRTACERAKRTLSSTAQTTIEID
BrHsp70-2e TAGDTHLGSEDFDNRMVNHFVQEFKRK--SKKDIS--------------GNPRALRRLRTACERAKRTLSSTAQTAIEID
BrHsp70-2f TAGDTHLGGEDFDNRMVNHFVQEFKRK--SKKDIT--------------GNPRALRRLRTACERAKRTLSSTAQTTIEID
BoHsp70-2a TAGDTHLGGEDFDNRMVNHFVQEFKRK--SKKDIT--------------GNPRALRRLRTACERAKRTLSSTAQTTIEID
BoHsp70-2b TAGDTHLGGEDFDNRMVNHFVQEFKRK--SKKDIT--------------GNPRALRRLRTACERAKRTLSSTAQTTIEID
BoHsp70-2c TAGDTHLGGEDFDNRMVNHFVQEFKRK--SKKDIS--------------GNPRALRRLRTACERAKRTLSSTAQTTIEID
BoHsp70-2d TAGDTHLGGEDFDNRMVNHFVQEFKRK--SKKDIS--------------GNPRALRRLRTACERAKRTLSSTAQTTIEID
BnC.Hsp70-2 TSGDTHLSGEDFDSRIVNHFVQEFKRK--SKKDIS--------------GNPRALRRLRTACERAKRTLSSTAQTTIEID
BrHsp70-4a TAGDTHLGGEDFDNRMVNHFVQEFKRK--HKKDIT--------------GNPRALRRLRTACERAKRTLSSTAQTTIEID
BrHsp70-4b TAGDTHLGGEDFDNRMVNHFVQEFKRK--HKKDIT--------------GNPRALRRLRTACERAKRTLSSTAQTTIEID
BoHsp70-4 TAGDTHLGGEDFDNRMVNHFVQEFKRK--HKKDIT--------------GNPRALRRLRTACERAKRTLSSTAQTTIEID
BnA.Hsp70-4a TAGDTHLGGEDFDNRMVNHFVQEFKRK--HKKDIT--------------GNPRALRRLRTACERAKRTLSSTAQTTIEID
BnC.Hsp70-4b TAGDTHLGGEDFDNRMVNHFVQEFKRK--HKKDIT--------------GNPRALRRLRTACERAKRTLSSTAQTTIEID
BnA.Hsp70-4c TAGDTHLGGEDFDNRMVNHFVQEFKRK--HKKDIT--------------GNPRALRRLRTACERAKRTLSSTAQTTIEID
BnC.Hsp70-4d TAGDTHLGGEDFDNRMVNHFVQEFKRK--HKKDIT--------------GNPRALRRLRTACERAKRTLSSTAQTTIEID
BrHsp70-5a TAGDTHLGGEDFDNRLVNHFVAEFKRK--HKKDVS--------------GNARALRRLRTACERAKRTLSSTAQTTIEID
BrHsp70-5b TAGDTHLGGEDFDNRLVNHFVAEFKRK--HKKDIS--------------GNARALRRLRTACERAKRTLSSTAQTTIEID
BoHsp70-5a TAGDTHLGGEDFDNRLVNHFVAEFRRK--HKKDIS--------------GNARALRRLRTACERAKRTLSSTAQTTIEID
BoHsp70-5b TAGDTHLGGEDFDNRLVNHFVAEFKRK--HKKDIS--------------GNARALRRLRTACERAKRTLSSTAQTTIEID
BnC.Hsp70-5a TAGDTHLGGEDFDNRLVNHFVAEFRRK--HKKDIS--------------GNARALRRLRTACERAKRTLSSTAQTTIEID
BnA.Hsp70-5b TAGDTHLGGEDFDNRLVNHFVAEFRRK--HKKDIS--------------GNARALRRLRTACERAKRTLSSTAQTTIEID
BnC.Hsp70-5c TAGDTHLGGEDFDNRLVNHFVAEFKRK--HKKDVS--------------GNARALRRLRTACERAKRTLSSTAQTTIEID
BnA.Hsp70-5d TAGDTHLGGEDFDNRLVNHFVAEFKRK--HKKDVS--------------GNARALRRLRTACERAKRTLSSTAQTTIEID
BrHsp70-6a TSGDTHLGGDDFDKRVVDWLALNFKKD--EGIDLL--------------KDKQALQRLTEAAEKAKIELSSLTQTNMSLP
BrHsp70-6b TSGDTHLGGDDFDKRVVDWLASNFKKD--EGIDLL--------------KDKQALQRLTEAAEKAKIELSSLTQTNMSLP
BrHsp70-6c TSGDTHLGGDDFDKRVVDWLASNFKKD--EGIDLL--------------KDKQALQRLTEAAEKAKIELSSLTQTNMSLP
BrHsp70-6d --
BnC.Hsp70-6a TSGDTHLGGDDFDKRVVDWLASNFKKD--EGIDLL--------------KDKQALQRLTEAAEKAKIELSSLTQTNMSLP
BnC.Hsp70-6b TSGDTHLGGDDFDKRVVDWLASNFKKD--EGIDLL--------------KDKQALQRLTEAAEKAKIELSSLTQTNMSLP
BnC.Hsp70-6c TCGDTHLGGDDFDKRVVDWLASNFKKD--EGIDLL--------------KDKQALQRLTEAAEKAKIELSTQTQTNISLP
BnC.Hsp70-6d TSGDKHLGGDDFDK--RLTEAAEKAKIELSSVTQTNISLP
BoHsp70-7 TSGDTHLGGDDFDKRVVDWLASNFKKD--EGIDLL--------------KDKQALQRLTEAAEKAKIELSSLTQTNMSLP
BnA.Hsp70-7a TSGDTHLGGDDFDKRVVDWLASNFKKD--EGIDLL--------------KDKQALQRLTEAAEKAKIELSSLTQTNMSLP
BnA.Hsp70-7b TSGDTHLGGDDFDKRVVDWLALNFKKD--EGIDLL--------------KDKQALQRLTEAAEKAKIELSSLTQTNMSLP
BnC.Hsp70-7c TSGDTHLGGDDFDKRVVDSLASNFKKD--EGIDLL--------------KDKQALQRLTEAAEKAKIELSSLTQTNMSLP
BnA.Hsp70-7d TSGDTHLGGDDFDKRVVDWLASNFKKD--EGIDLL--------------KDKQALQRLTEAAEKAKIELSSLTQTNMSLP
BrHsp70-8 LAG-SAVGGEDILQNTMRHVAPR--------------------------PDKEGSGSLRVATQDAIHRLSKQESVQIEVD
BoHsp70-8 LAG-SAVGGEDILQNTMRHIAPR--------------------------PDKEGSGSLRVATQDAIHRLSKQDSVQIEVD
BnA.Hsp70-8a LAG-SAVGGEDILQNTLRHVAPR--------------------------PGKEGSGSLRVATQDAIHRLSKQESVQIEVD
BnC.Hsp70-8b LAG-SAVGGEDILQNTMRHIAPR--------------------------PDKEGSGSLRVATQDAIHRLSKQDSVQIEVD
BrHsp70-9a TNGDTFLGGEDFDNTLLEYLVSEFKRS--DNIDLT--------------KDKLALQRLREAAEKAKIELSSTSQTEINLP
BrHsp70-9b TNGDTFLGGEDFDNTLLEYLVSEFKRS--DNIDLT--------------KDKLALQRLREAAEKAKIELSSTSQTEINLP
BoHsp70-9a TNGDTFLGGEDFDNTLLEYLVSEFKRS--DNIDLT--------------KDKLALQRLREAAEKAKIELSSTSQTEINLP
BoHsp70-9b TNGDTFLGGEDFDNTLLEYLVSEFKRS--DNIDLT--------------KDKLALQRLREAAEKAKIELSSTSQTEINLP
BnC.Hsp70-9a TNGDTFLGGEDFDNTLLEYLVSEFKRS--DNIDLT--------------KDKLALQRLREAAEKAKIELSSTSQTEINLP
BnC.Hsp70-9b TNGDTFLGGEDFDNTLLEYLVSEFKRS--DNIDLT--------------KDKLALQRLREAAEKAKIELSSTSQTEINLP
BnA.Hsp70-9c TNGDTFLGGEDFDNTLLEYLVSEFKRS--DNIDLT--------------KDKLALQRLREAAEKAKIELSSTSQTEINLP
BnA.Hsp70-9d TNGDTFLGGEDFDNTLLEYLVSEFKRS--DNIDLT--------------KDKLALQRLREAAEKAKIELSSTSQTEINLP
BrHsp70-10a TNGDTFLGGEDFDNALLDFLVNEFKTS--EGIDLA--------------KDRLALQRLREAAEKAKIELSSTSQTEINLP
BrHsp70-10b TNGDTFLGGEDFDNALLDFLVNEFKTS--EGIDLA--------------KDRLALQRLREAAEKAKIELSSTSQTEINLP
BoHsp70-10 TNGDTFLGGEDFDNALLDFLVNEFKTS--EGIDLA--------------KDRLALQRLREAAEKAKIELSSTSQTEINLP
BnA.Hsp70-10a TNGDTFLGGEDFDNALLDFLVNEFKTS--EGIDLA--------------KDRLALQRLREAAEKAKIELSSTSQTEINLP
BnA.Hsp70-10b TNGDTFLGGEDFDNALLDFLVNEFKTS--EGIDLA--------------KDRLALQRLREAAEKAKIELSSTSQTEINLP
BnC.Hsp70-10c TNGDTFLGGEDFDNALLDFLVNEFKTS--EGIDLA--------------KDRLALQRLREAAEKAKIELSSTSQTEINLP
BnC.Hsp70-10d TNGDTFLGGEDFDNALLDFLVNEFKTS--EGIDLA--------------RDRLALQRLREAAEKAKIELSSTSQTEINLP

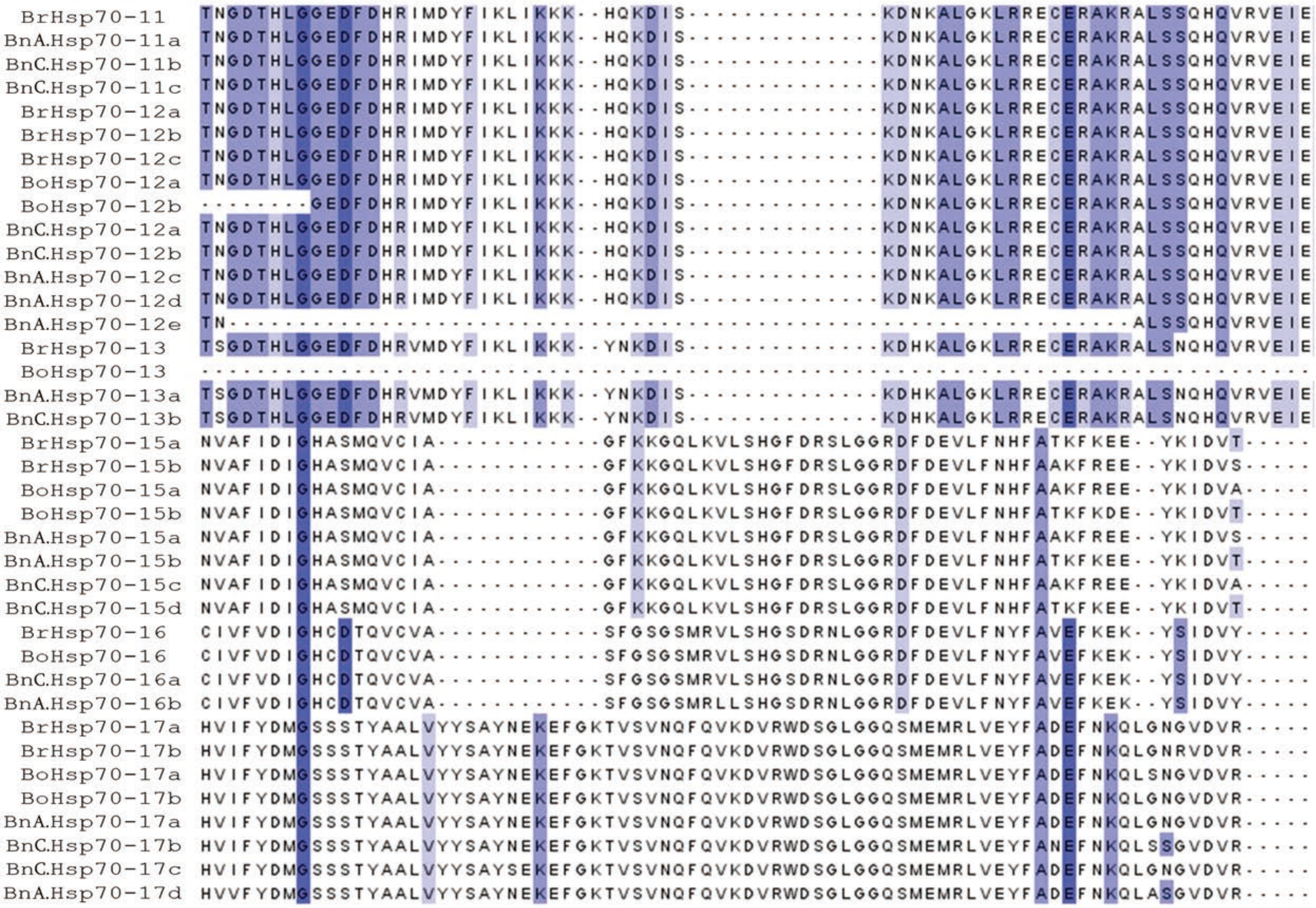

BrHsp70-11 TNGDTHLGGEDFDHRIMDYFIKLIKKK--HQKDIS--------------KDNKALGKLRRECERAKRALSSQHQVRVEIE
BnA.Hsp70-11a TNGDTHLGGEDFDHRIMDYFIKLIKKK--HQKDIS--------------KDNKALGKLRRECERAKRALSSQHQVRVEIE
BnC.Hsp70-11b TNGDTHLGGEDFDHRIMDYFIKLIKKK--HQKDIS--------------KDNKALGKLRRECERAKRALSSQHQVRVEIE
BnC.Hsp70-11c TNGDTHLGGEDFDHRIMDYFIKLIKKK--HQKDIS--------------KDNKALGKLRRECERAKRALSSQHQVRVEIE
BrHsp70-12a TNGDTHLGGEDFDHRIMDYFIKLIKKK--HQKDIS--------------KDNKALGKLRRECERAKRALSSQHQVRVEIE
BrHsp70-12b TNGDTHLGGEDFDHRIMDYFIKLIKKK--HQKDIS--------------KDNKALGKLRRECERAKRALSSQHQVRVEIE
BrHsp70-12c TNGDTHLGGEDFDHRIMDYFIKLIKKK--HQKDIS--------------KDNKALGKLRRECERAKRALSSQHQVRVEIE
BoHsp70-12a TNGDTHLGGEDFDHRIMDYFIKLIKKK--HQKDIS--------------KDNKALGKLRRECERAKRALSSQHQVRVEIE
BoHsp70-12b --------GEDFDHRIMDYFIKLIKKK--HQKDIS--------------KDNKALGKLRRECERAKRALSSQHQVRVEIE
BnC.Hsp70-12a TNGDTHLGGEDFDHRIMDYFIKLIKKK--HQKDIS--------------KDNKALGKLRRECERAKRALSSQHQVRVEIE
BnC.Hsp70-12b TNGDTHLGGEDFDHRIMDYFIKLIKKK--HQKDIS--------------KDNKALGKLRRECERAKRALSSQHQVRVEIE
BnA.Hsp70-12c TNGDTHLGGEDFDHRIMDYFIKLIKKK--HQKDIS--------------KDNKALGKLRRECERAKRALSSQHQVRVEIE
BnA.Hsp70-12d TNGDTHLGGEDFDHRIMDYFIKLIKKK--HQKDIS--------------KDNKALGKLRRECERAKRALSSQHQVRVEIE
BnA.Hsp70-12e TN---ALSSQHQVRVEIE
BrHsp70-13 TSGDTHLGGEDFDHRVMDYFIKLIKKK--YNKDIS--------------KDHKALGKLRRECERAKRALSNQHQVRVEIE
BoHsp70-13 --
BnA.Hsp70-13a TSGDTHLGGEDFDHRVMDYFIKLIKKK--YNKDIS--------------KDHKALGKLRRECERAKRALSNQHQVRVEIE
BnC.Hsp70-13b TSGDTHLGGEDFDHRVMDYFIKLIKKK--YNKDIS--------------KDHKALGKLRRECERAKRALSNQHQVRVEIE
BrHsp70-15a NVAFIDIGHASMQVCIA------------GFKKGQLKVLSHGFDRSLGGRDFDEVLFNHFATKFKEE--YKIDVT-----
BrHsp70-15b NVAFIDIGHASMQVCIA------------GFKKGQLKVLSHGFDRSLGGRDFDEVLFNHFAAKFREE--YKIDVS-----
BoHsp70-15a NVAFIDIGHASMQVCIA------------GFKKGQLKVLSHGFDRSLGGRDFDEVLFNHFAAKFREE--YKIDVA-----
BoHsp70-15b NVAFIDIGHASMQVCIA------------GFKKGQLKVLSHGFDRSLGGRDFDEVLFNHFATKFKDE--YKIDVT-----
BnA.Hsp70-15a NVAFIDIGHASMQVCIA------------GFKKGQLKVLSHGFDRSLGGRDFDEVLFNHFAAKFREE--YKIDVS-----
BnA.Hsp70-15b NVAFIDIGHASMQVCIA------------GFKKGQLKVLSHGFDRSLGGRDFDEVLFNHFATKFKEE--YKIDVT-----
BnC.Hsp70-15c NVAFIDIGHASMQVCIA------------GFKKGQLKVLSHGFDRSLGGRDFDEVLFNHFAAKFREE--YKIDVA-----
BnC.Hsp70-15d NVAFIDIGHASMQVCIA------------GFKKGQLKVLSHGFDRSLGGRDFDEVLFNHFATKFKEE--YKIDVT-----
BrHsp70-16 CIVFVDIGHCDTQVCVA------------SFGSGSMRVLSHGSDRNLGGRDFDEVLFNYFAVEFKEK--YSIDVY-----
BoHsp70-16 CIVFVDIGHCDTQVCVA------------SFGSGSMRVLSHGSDRNLGGRDFDEVLFNYFAVEFKEK--YSIDVY-----
BnC.Hsp70-16a CIVFVDIGHCDTQVCVA------------SFGSGSMRVLSHGSDRNLGGRDFDEVLFNYFAVEFKEK--YSIDVY-----
BnA.Hsp70-16b CIVFVDIGHCDTQVCVA------------SFGSGSMRLLSHGSDRNLGGRDFDEVLFNYFAVEFKEK--YSIDVY-----
BrHsp70-17a HVIFYDMGSSSTYAALVYYSAYNEKEFGKTVSVNQFQVKDVRWDSGLGGQSMEMRLVEYFADEFNKQLGNGVDVR-----
BrHsp70-17b HVIFYDMGSSSTYAALVYYSAYNEKEFGKTVSVNQFQVKDVRWDSGLGGQSMEMRLVEYFADEFNKQLGNRVDVR-----
BoHsp70-17a HVIFYDMGSSSTYAALVYYSAYNEKEFGKTVSVNQFQVKDVRWDSGLGGQSMEMRLVEYFADEFNKQLSNGVDVR-----
BoHsp70-17b HVIFYDMGSSSTYAALVYYSAYNEKEFGKTVSVNQFQVKDVRWDSGLGGQSMEMRLVEYFADEFNKQLGNGVDVR-----
BnA.Hsp70-17a HVIFYDMGSSSTYAALVYYSAYNEKEFGKTVSVNQFQVKDVRWDSGLGGQSMEMRLVEYFADEFNKQLGNGVDVR-----
BnC.Hsp70-17b HVIFYDMGSSSTYAALVYYSAYNEKEFGKTVSVNQFQVKDVRWDSGLGGQSMEMRLVEYFANEFNKQLSSGVDVR-----
BnC.Hsp70-17c HVIFYDMGSSSTYAALVYYSAYSEKEFGKTVSVNQFQVKDVRWDSGLGGQSMEMRLVEYFADEFNKQLGNGVDVR-----
BnA.Hsp70-17d HVVFYDMGSSSTYAALVYYSAYNEKEFGKTVSVNQFQVKDVRWDSGLGGQSMEMRLVEYFADEFNKQLASGVDVR-----

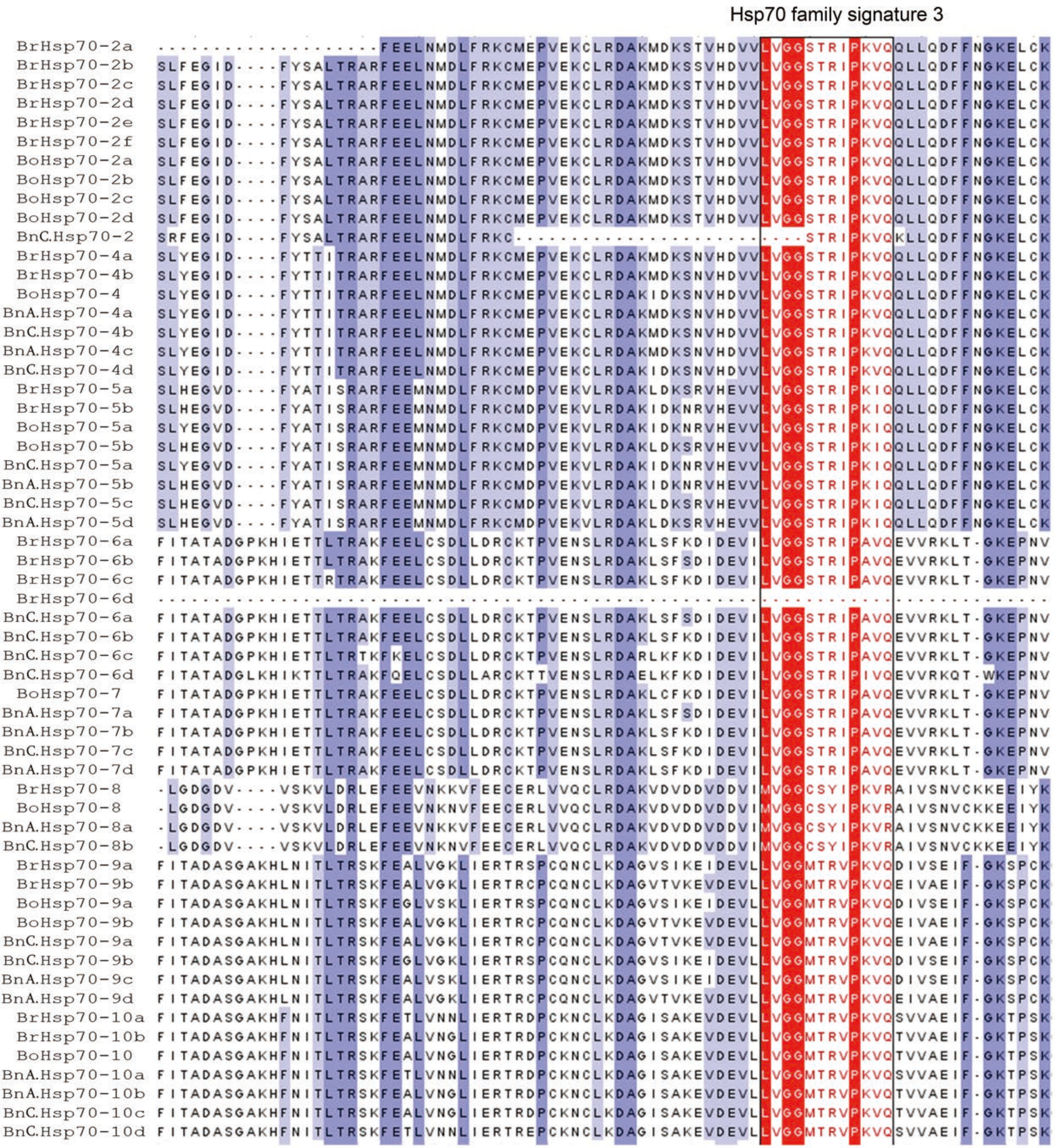
Hsp70 family signature 3
BrHsp70-2a
BrHsp70-2b
BrHsp70-2c
BrHsp70-2d
BrHsp70-2e
BrHsp70-2f
BoHsp70-2a
BoHsp70-2b
BoHsp70-2c
BoHsp70-2d
BnC.Hsp70-2
BrHsp70-4a
BrHsp70-4b
BoHsp70-4
BnA.Hsp70-4a
BnC.Hsp70-4b
BnA.Hsp70-4c
BnC.Hsp70-4d
BrHsp70-5a
BrHsp70-5b
BoHsp70-5a
BoHsp70-5b
BnC.Hsp70-5a
BnA.Hsp70-5b
BnC.Hsp70-5c
BnA.Hsp70-5d
BrHsp70-6a
BrHsp70-6b
BrHsp70-6c
BrHsp70-6d
BnC.Hsp70-6a
BnC.Hsp70-6b
BnC.Hsp70-6c
BnC.Hsp70-6d
BoHsp70-7
BnA.Hsp70-7a
BnA.Hsp70-7b
BnC.Hsp70-7c
BnA.Hsp70-7d
BrHsp70-8
BoHsp70-8
BnA.Hsp70-8a
BnC.Hsp70-8b
BrHsp70-9a
BrHsp70-9b
BoHsp70-9a
BoHsp70-9b
BnC.Hsp70-9a
BnC.Hsp70-9b
BnA.Hsp70-9c
BnA.Hsp70-9d
BrHsp70-10a
BrHsp70-10b
BoHsp70-10
BnA.Hsp70-10a
BnA.Hsp70-10b
BnC.Hsp70-10c
BnC.Hsp70-10d

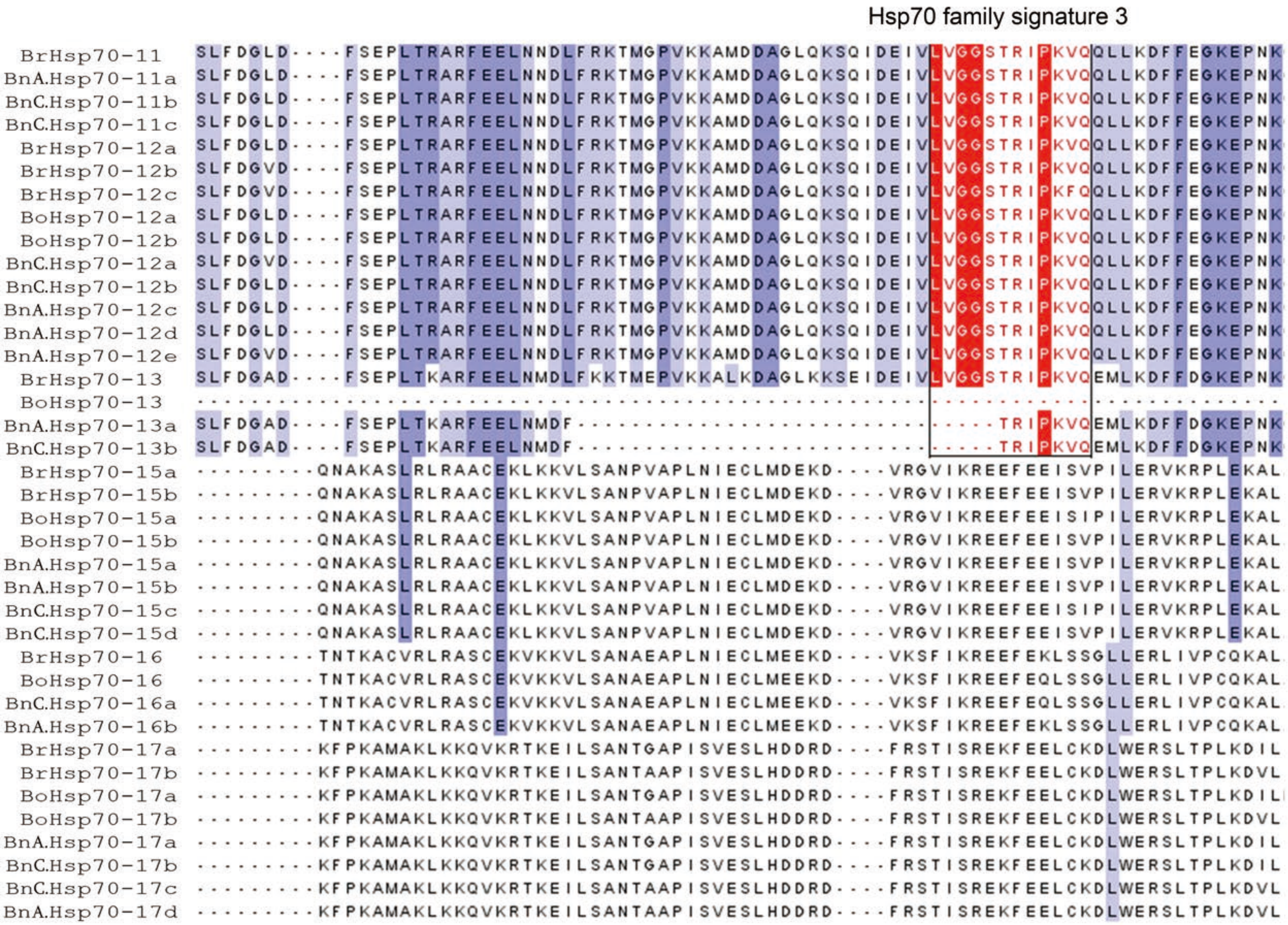
Hsp70 family signature 3
BrHsp70-11 SLFDGLD----FSEPLTRARFEELNNDLFRKTMGPVKKAMDDAGLQKSQIDEIVLVGGSTRIPKVQQLLKDFFEGKEPNK
BnA.Hsp70-11a SLFDGLD----FSEPLTRARFEELNNDLFRKTMGPVKKAMDDAGLQKSQIDEIVLVGGSTRIPKVQQLLKDFFEGKEPNK
BnC.Hsp70-11b SLFDGLD----FSEPLTRARFEELNNDLFRKTMGPVKKAMDDAGLQKSQIDEIVLVGGSTRIPKVQQLLKDFFEGKEPNK
BnC.Hsp70-11c SLFDGLD----FSEPLTRARFEELNNDLFRKTMGPVKKAMDDAGLQKSQIDEIVLVGGSTRIPKVQQLLKDFFEGKEPNK
BrHsp70-12a SLFDGLD----FSEPLTRARFEELNNDLFRKTMGPVKKAMDDAGLQKSQIDEIVLVGGSTRIPKVQQLLKDFFEGKEPNK
BrHsp70-12b SLFDGVD----FSEPLTRARFEELNNDLFRKTMGPVKKAMDDAGLQKSQIDEIVLVGGSTRIPKVQQLLKDFFEGKEPNK
BrHsp70-12c SLFDGVD----FSEPLTRARFEELNNDLFRKTMGPVKKAMDDAGLQKSQIDEIVLVGGSTRIPKFQQLLKDFFEGKEPNK
BoHsp70-12a SLFDGLD----FSEPLTRARFEELNNDLFRKTMGPVKKAMDDAGLQKSQIDEIVLVGGSTRIPKVQQLLKDFFEGKEPNK
BoHsp70-12b SLFDGLD----FSEPLTRARFEELNNDLFRKTMGPVKKAMDDAGLQKSQIDEIVLVGGSTRIPKVQQLLKDFFEGKEPNK
BnC.Hsp70-12a SLFDGVD----FSEPLTRARFEELNNDLFRKTMGPVKKAMDDAGLQKSQIDEIVLVGGSTRIPKVQQLLKDFFEGKEPNK
BnC.Hsp70-12b SLFDGLD----FSEPLTRARFEELNNDLFRKTMGPVKKAMDDAGLQKSQIDEIVLVGGSTRIPKVQQLLKDFFEGKEPNK
BnA.Hsp70-12c SLFDGLD----FSEPLTRARFEELNNDLFRKTMGPVKKAMDDAGLQKSQIDEIVLVGGSTRIPKVQQLLKDFFEGKEPNK
BnA.Hsp70-12d SLFDGLD----FSEPLTRARFEELNNDLFRKTMGPVKKAMDDAGLQKSQIDEIVLVGGSTRIPKVQQLLKDFFEGKEPNK
BnA.Hsp70-12e SLFDGVD----FSEPLTRARFEELNNDLFRKTMGPVKKAMDDAGLQKSQIDEIVLVGGSTRIPKVQQLLKDFFEGKEPNK
BrHsp70-13 SLFDGAD----FSEPLTKARFEELNMDLFKKTMEPVKKALKDAGLKKSEIDEIVLVGGSTRIPKVQEMLKDFFDGKEPNK
BoHsp70-13 --
BnA.Hsp70-13a SLFDGAD----FSEPLTKARFEELNMDF-------------------------------TRIPKVQEMLKDFFDGKEPNK
BnC.Hsp70-13b SLFDGAD----FSEPLTKARFEELNMDF-------------------------------TRIPKVQEMLKDFFDGKEPNK
BrHsp70-15a ---------QNAKASLRLRAACEKLKKVLSANPVAPLNIECLMDEKD----VRGVIKREEFEEISVPILERVKRPLEKAL
BrHsp70-15b ---------QNAKASLRLRAACEKLKKVLSANPVAPLNIECLMDEKD----VRGVIKREEFEEISVPILERVKRPLEKAL
BoHsp70-15a ---------QNAKASLRLRAACEKLKKVLSANPVAPLNIECLMDEKD----VRGVIKREEFEEISIPILERVKRPLEKAL
BoHsp70-15b ---------QNAKASLRLRAACEKLKKVLSANPVAPLNIECLMDEKD----VRGVIKREEFEEISVPILERVKRPLEKAL
BnA.Hsp70-15a ---------QNAKASLRLRAACEKLKKVLSANPVAPLNIECLMDEKD----VRGVIKREEFEEISVPILERVKRPLEKAL
BnA.Hsp70-15b ---------QNAKASLRLRAACEKLKKVLSANPVAPLNIECLMDEKD----VRGVIKREEFEEISVPILERVKRPLEKAL
BnC.Hsp70-15c ---------QNAKASLRLRAACEKLKKVLSANPVAPLNIECLMDEKD----VRGVIKREEFEEISIPILERVKRPLEKAL
BnC.Hsp70-15d ---------QNAKASLRLRAACEKLKKVLSANPVAPLNIECLMDEKD----VRGVIKREEFEEISVPILERVKRPLEKAL
BrHsp70-16 ---------TNTKACVRLRASCEKVKKVLSANAEAPLNIECLMEEKD----VKSFIKREEFEKLSSGLLERLIVPCQKAL
BoHsp70-16 ---------TNTKACVRLRASCEKVKKVLSANAEAPLNIECLMEEKD----VKSFIKREEFEQLSSGLLERLIVPCQKAL
BnC.Hsp70-16a ---------TNTKACVRLRASCEKVKKVLSANAEAPLNIECLMEEKD----VKSFIKREEFEQLSSGLLERLIVPCQKAL
BnA.Hsp70-16b ---------TNTKACVRLRASCEKVKKVLSANAEAPLNIECLMEEKD----VKSFIKREEFEKLSSGLLERLIVPCQKAL
BrHsp70-17a ---------KFPKAMAKLKKQVKRTKEILSANTGAPISVESLHDDRD----FRSTISREKFEELCKDLWERSLTPLKDIL
BrHsp70-17b ---------KFPKAMAKLKKQVKRTKEILSANTAAPISVESLHDDRD----FRSTISREKFEELCKDLWERSLTPLKDVL
BoHsp70-17a ---------KFPKAMAKLKKQVKRTKEILSANTGAPISVESLHDDRD----FRSTISREKFEELCKDLWERSLTPLKDIL
BoHsp70-17b ---------KFPKAMAKLKKQVKRTKEILSANTAAPISVESLHDDRD----FRSTISREKFEELCKDLWERSLTPLKDVL
BnA.Hsp70-17a ---------KFPKAMAKLKKQVKRTKEILSANTGAPISVESLHDDRD----FRSTISREKFEELCKDLWERSLTPLKDIL
BnC.Hsp70-17b ---------KFPKAMAKLKKQVKRTKEILSANTGAPISVESLHDDRD----FRSTISREKFEELCKDLWERSLTPLKDIL
BnC.Hsp70-17c ---------KFPKAMAKLKKQVKRTKEILSANTAAPISVESLHDDRD----FRSTISREKFEELCKDLWERSLTPLKDVL
BnA.Hsp70-17d ---------KFPKAMAKLKKQVKRTKEILSANTAAPISVESLHDDRD----FRSTISREKFEELCKDLWERSLTPLKDVL

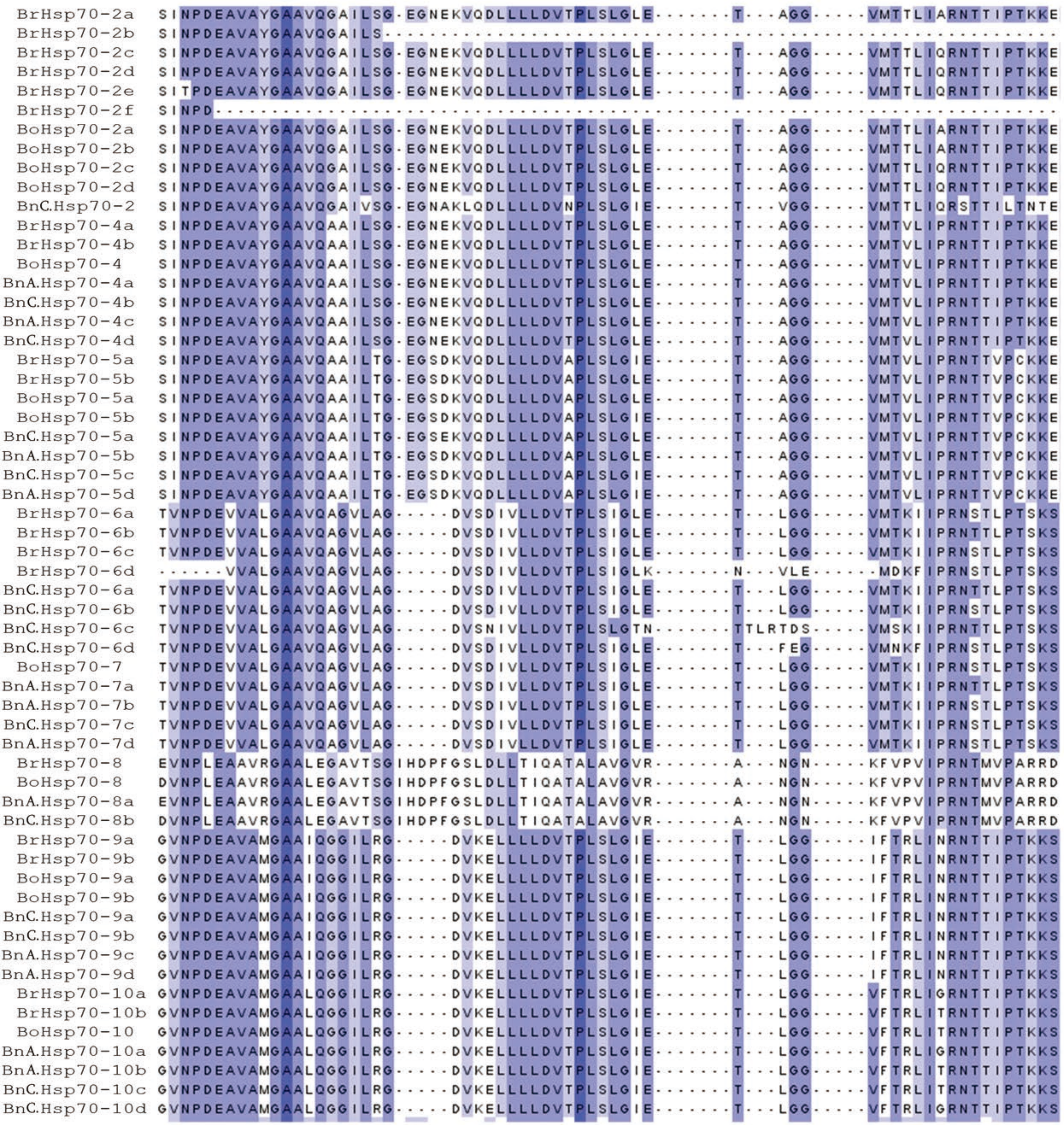
BrHsp70-2a SINPDEAVAYGAAVQGAILSG-EGNEKVQDLLLLDVTPLSLGLE-------T---AGG-----VMTTLIARNTTIPTKKE
BrHsp70-2b SINPDEAVAYGAAVQGAILS--
BrHsp70-2c SINPDEAVAYGAAVQGAILSG-EGNEKVQDLLLLDVTPLSLGLE-------T---AGG-----VMTTLIQRNTTIPTKKE
BrHsp70-2d SINPDEAVAYGAAVQGAILSG-EGNEKVQDLLLLDVTPLSLGLE-------T---AGG-----VMTTLIQRNTTIPTKKE
BrHsp70-2e SITPDEAVAYGAAVQGAILSG-EGNEKVQDLLLLDVTPLSLGLE-------T---AGG-----VMTTLIQRNTTIPTKKE
BrHsp70-2f SINPD---
BoHsp70-2a SINPDEAVAYGAAVQGAILSG-EGNEKVQDLLLLDVTPLSLGLE-------T---AGG-----VMTTLIARNTTIPTKKE
BoHsp70-2b SINPDEAVAYGAAVQGAILSG-EGNEKVQDLLLLDVTPLSLGLE-------T---AGG-----VMTTLIARNTTIPTKKE
BoHsp70-2c SINPDEAVAYGAAVQGAILSG-EGNEKVQDLLLLDVTPLSLGLE-------T---AGG-----VMTTLIQRNTTIPTKKE
BoHsp70-2d SINPDEAVAYGAAVQGAILSG-EGNEKVQDLLLLDVTPLSLGLE-------T---AGG-----VMTTLIQRNTTIPTKKE
BnC.Hsp70-2 SINPDEAVAYGAAVQGAIVSG-EGNAKLQDLLLLDVNPLSLGIE-------T---VGG-----VMTTLIQRSTTILTNTE
BrHsp70-4a SINPDEAVAYGAAVQAAILSG-EGNEKVQDLLLLDVTPLSLGLE-------T---AGG-----VMTVLIPRNTTIPTKKE
BrHsp70-4b SINPDEAVAYGAAVQAAILSG-EGNEKVQDLLLLDVTPLSLGLE-------T---AGG-----VMTVLIPRNTTIPTKKE
BoHsp70-4 SINPDEAVAYGAAVQAAILSG-EGNEKVQDLLLLDVTPLSLGLE-------T---AGG-----VMTVLIPRNTTIPTKKE
BnA.Hsp70-4a SINPDEAVAYGAAVQAAILSG-EGNEKVQDLLLLDVTPLSLGLE-------T---AGG-----VMTVLIPRNTTIPTKKE
BnC.Hsp70-4b SINPDEAVAYGAAVQAAILSG-EGNEKVQDLLLLDVTPLSLGLE-------T---AGG-----VMTVLIPRNTTIPTKKE
BnA.Hsp70-4c SINPDEAVAYGAAVQAAILSG-EGNEKVQDLLLLDVTPLSLGLE-------T---AGG-----VMTVLIPRNTTIPTKKE
BnC.Hsp70-4d SINPDEAVAYGAAVQAAILSG-EGNEKVQDLLLLDVTPLSLGLE-------T---AGG-----VMTVLIPRNTTIPTKKE
BrHsp70-5a SINPDEAVAYGAAVQAAILTG-EGSDKVQDLLLLDVAPLSLGIE-------T---AGG-----VMTVLIPRNTTVPCKKE
BrHsp70-5b SINPDEAVAYGAAVQAAILTG-EGSDKVQDLLLLDVAPLSLGLE-------T---AGG-----VMTVLIPRNTTVPCKKE
BoHsp70-5a SINPDEAVAYGAAVQAAILTG-EGSDKVQDLLLLDVAPLSLGLE-------T---AGG-----VMTVLIPRNTTVPCKKE
BoHsp70-5b SINPDEAVAYGAAVQAAILTG-EGSDKVQDLLLLDVAPLSLGIE-------T---AGG-----VMTVLIPRNTTVPCKKE
BnC.Hsp70-5a SINPDEAVAYGAAVQAAILTG-EGSEKVQDLLLLDVAPLSLGLE-------T---AGG-----VMTVLIPRNTTVPCKKE
BnA.Hsp70-5b SINPDEAVAYGAAVQAAILTG-EGSDKVQDLLLLDVAPLSLGLE-------T---AGG-----VMTVLIPRNTTVPCKKE
BnC.Hsp70-5c SINPDEAVAYGAAVQAAILTG-EGSDKVQDLLLLDVAPLSLGIE-------T---AGG-----VMTVLIPRNTTVPCKKE
BnA.Hsp70-5d SINPDEAVAYGAAVQAAILTG-EGSDKVQDLLLLDVAPLSLGIE-------T---AGG-----VMTVLIPRNTTVPCKKE
BrHsp70-6a TVNPDEVVALGAAVQAGVLAG-----DVSDIVLLDVTPLSIGLE-------T---LGG-----VMTKIIPRNSTLPTSKS
BrHsp70-6b TVNPDEVVALGAAVQAGVLAG-----DVSDIVLLDVTPLSIGLE-------T---LGG-----VMTKIIPRNTTLPTSKS
BrHsp70-6c TVNPDEVVALGAAVQAGVLAG-----DVSDIVLLDVTPLSIGLE-------T---LGG-----VMTKIIPRNSTLPTSKS
BrHsp70-6d ------VVALGAAVQAGVLAG-----DVSDIVLLDVTPLSIGLK-------N---VLE------MDKFIPRNSTLPTSKS
BnC.Hsp70-6a TVNPDEVVALGAAVQAGVLAG-----DVSDIVLLDVTPLSIGLE-------T---LGG-----VMTKIIPRNTTLPTSKS
BnC.Hsp70-6b TVNPDEVVALGAAVQAGVLAG-----DVSDIVLLDVTPLSIGLE-------T---LGG-----VMTKIIPRNSTLPTSKS
BnC.Hsp70-6c TVNPDEVVALGAAVQAGVLAG-----DVSNIVLLDVTPLSLGTN-------TTLRTDS-----VMSKIIPRNTTLPTSKS
BnC.Hsp70-6d TVNPDEVVALGAAVQAGVLAG-----DVSDIVLLDVTPLSIGLE-------T---FEG-----VMNKFIPRNSTLPTSKS
BoHsp70-7 TVNPDEVVALGAAVQAGVLAG-----DVSDIVLLDVTPLSIGLE-------T---LGG-----VMTKIIPRNSTLPTSKS
BnA.Hsp70-7a TVNPDEVVALGAAVQAGVLAG-----DVSDIVLLDVTPLSIGLE-------T---LGG-----VMTKIIPRNTTLPTSKS
BnA.Hsp70-7b TVNPDEVVALGAAVQAGVLAG-----DVSDIVLLDVTPLSIGLE-------T---LGG-----VMTKIIPRNSTLPTSKS
BnC.Hsp70-7c TVNPDEVVALGAAVQAGVLAG-----DVSDIVLLDVTPLSIGLE-------T---LGG-----VMTKIIPRNSTLPTSKS
BnA.Hsp70-7d TVNPDEVVALGAAVQAGVLAG-----DVSDIVLLDVTPLSIGLE-------T---LGG-----VMTKIIPRNSTLPTSKS
BrHsp70-8 EVNPLEAAVRGAALEGAVTSGIHDPFGSLDLLTIQATALAVGVR-------A---NGN-----KFVPVIPRNTMVPARRD
BoHsp70-8 DVNPLEAAVRGAALEGAVTSGIHDPFGSLDLLTIQATALAVGVR-------A---NGN-----KFVPVIPRNTMVPARRD
BnA.Hsp70-8a EVNPLEAAVRGAALEGAVTSGIHDPFGSLDLLTIQATALAVGVR-------A---NGN-----KFVPVIPRNTMVPARRD
BnC.Hsp70-8b DVNPLEAAVRGAALEGAVTSGIHDPFGSLDLLTIQATALAVGVR-------A---NGN-----KFVPVIPRNTMVPARRD
BrHsp70-9a GVNPDEAVAMGAAIQGGILRG-----DVKELLLLDVTPLSLGIE-------T---LGG-----IFTRLINRNTTIPTKKS
BrHsp70-9b GVNPDEAVAMGAAIQGGILRG-----DVKELLLLDVTPLSLGIE-------T---LGG-----IFTRLINRNTTIPTKKS
BoHsp70-9a GVNPDEAVAMGAAIQGGILRG-----DVKELLLLDVTPLSLGIE-------T---LGG-----IFTRLINRNTTIPTKKS
BoHsp70-9b GVNPDEAVAMGAAIQGGILRG-----DVKELLLLDVTPLSLGIE-------T---LGG-----IFTRLINRNTTIPTKKS
BnC.Hsp70-9a GVNPDEAVAMGAAIQGGILRG-----DVKELLLLDVTPLSLGIE-------T---LGG-----IFTRLINRNTTIPTKKS
BnC.Hsp70-9b GVNPDEAVAMGAAIQGGILRG-----DVKELLLLDVTPLSLGIE-------T---LGG-----IFTRLINRNTTIPTKKS
BnA.Hsp70-9c GVNPDEAVAMGAAIQGGILRG-----DVKELLLLDVTPLSLGIE-------T---LGG-----IFTRLINRNTTIPTKKS
BnA.Hsp70-9d GVNPDEAVAMGAAIQGGILRG-----DVKELLLLDVTPLSLGIE-------T---LGG-----IFTRLINRNTTIPTKKS
BrHsp70-10a GVNPDEAVAMGAALQGGILRG-----DVKELLLLDVTPLSLGIE-------T---LGG-----VFTRLIGRNTTIPTKKS
BrHsp70-10b GVNPDEAVAMGAALQGGILRG-----DVKELLLLDVTPLSLGIE-------T---LGG-----VFTRLITRNTTIPTKKS
BoHsp70-10 GVNPDEAVAMGAALQGGILRG-----DVKELLLLDVTPLSLGIE-------T---LGG-----VFTRLITRNTTIPTKKS
BnA.Hsp70-10a GVNPDEAVAMGAALQGGILRG-----DVKELLLLDVTPLSLGIE-------T---LGG-----VFTRLIGRNTTIPTKKS
BnA.Hsp70-10b GVNPDEAVAMGAALQGGILRG-----DVKELLLLDVTPLSLGIE-------T---LGG-----VFTRLITRNTTIPTKKS
BnC.Hsp70-10c GVNPDEAVAMGAALQGGILRG-----DVKELLLLDVTPLSLGIE-------T---LGG-----VFTRLITRNTTIPTKKS
BnC.Hsp70-10d GVNPDEAVAMGAALQGGILRG-----DVKELLLLDVTPLSLGIE-------T---LGG-----VFTRLIGRNTTIPTKKS

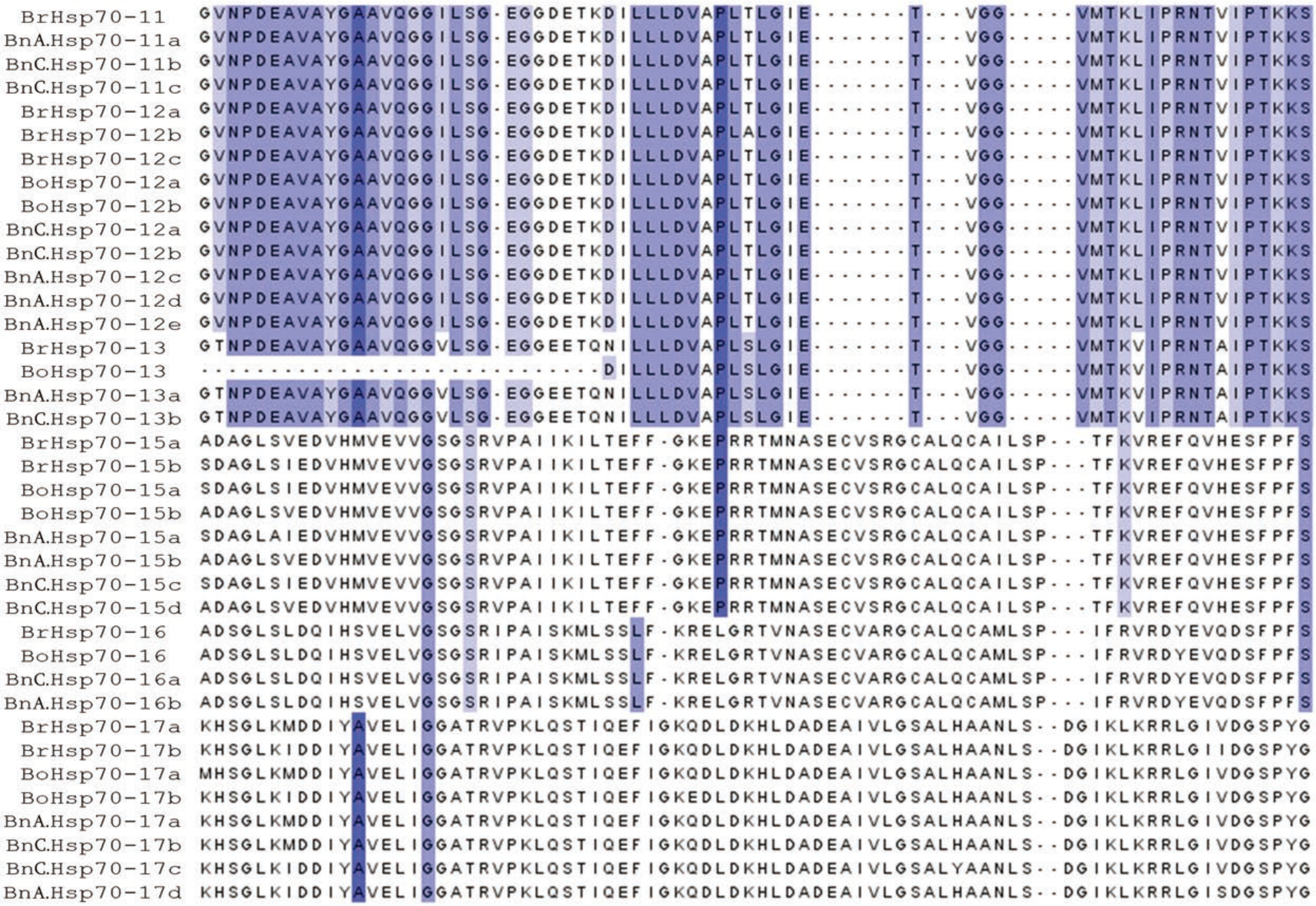
BrHsp70-11 GVNPDEAVAYGAAVQGGILSG-EGGDETKDILLLDVAPLTLGIE-------T---VGG-----VMTKLIPRNTVIPTKKS
BnA.Hsp70-11a GVNPDEAVAYGAAVQGGILSG-EGGDETKDILLLDVAPLTLGIE-------T---VGG-----VMTKLIPRNTVIPTKKS
BnC.Hsp70-11b GVNPDEAVAYGAAVQGGILSG-EGGDETKDILLLDVAPLTLGIE-------T---VGG-----VMTKLIPRNTVIPTKKS
BnC.Hsp70-11c GVNPDEAVAYGAAVQGGILSG-EGGDETKDILLLDVAPLTLGIE-------T---VGG-----VMTKLIPRNTVIPTKKS
BrHsp70-12a GVNPDEAVAYGAAVQGGILSG-EGGDETKDILLLDVAPLTLGIE-------T---VGG-----VMTKLIPRNTVIPTKKS
BrHsp70-12b GVNPDEAVAYGAAVQGGILSG-EGGDETKDILLLDVAPLALGIE-------T---VGG-----VMTKLIPRNTVIPTKKS
BrHsp70-12c GVNPDEAVAYGAAVQGGILSG-EGGDETKDILLLDVAPLTLGIE-------T---VGG-----VMTKLIPRNTVIPTKKS
BoHsp70-12a GVNPDEAVAYGAAVQGGILSG-EGGDETKDILLLDVAPLTLGIE-------T---VGG-----VMTKLIPRNTVIPTKKS
BoHsp70-12b GVNPDEAVAYGAAVQGGILSG-EGGDETKDILLLDVAPLTLGIE-------T---VGG-----VMTKLIPRNTVIPTKKS
BnC.Hsp70-12a GVNPDEAVAYGAAVQGGILSG-EGGDETKDILLLDVAPLTLGIE-------T---VGG-----VMTKLIPRNTVIPTKKS
BnC.Hsp70-12b GVNPDEAVAYGAAVQGGILSG-EGGDETKDILLLDVAPLTLGIE-------T---VGG-----VMTKLIPRNTVIPTKKS
BnA.Hsp70-12c GVNPDEAVAYGAAVQGGILSG-EGGDETKDILLLDVAPLTLGIE-------T---VGG-----VMTKLIPRNTVIPTKKS
BnA.Hsp70-12d GVNPDEAVAYGAAVQGGILSG-EGGDETKDILLLDVAPLTLGIE-------T---VGG-----VMTKLIPRNTVIPTKKS
BnA.Hsp70-12e GVNPDEAVAYGAAVQGGILSG-EGGDETKDILLLDVAPLTLGIE-------T---VGG-----VMTKLIPRNTVIPTKKS
BrHsp70-13 GTNPDEAVAYGAAVQGGVLSG-EGGEETQNILLLDVAPLSLGIE-------T---VGG-----VMTKVIPRNTAIPTKKS
BoHsp70-13 -----------------------------DILLLDVAPLSLGIE-------T---VGG-----VMTKVIPRNTAIPTKKS
BnA.Hsp70-13a GTNPDEAVAYGAAVQGGVLSG-EGGEETQNILLLDVAPLSLGIE-------T---VGG-----VMTKVIPRNTAIPTKKS
BnC.Hsp70-13b GTNPDEAVAYGAAVQGGVLSG-EGGEETQNILLLDVAPLSLGIE-------T---VGG-----VMTKVIPRNTAIPTKKS
BrHsp70-15a ADAGLSVEDVHMVEVVGSGSRVPAIIKILTEFF-GKEPRRTMNASECVSRGCALQCAILSP---TFKVREFQVHESFPFS
BrHsp70-15b SDAGLSIEDVHMVEVVGSGSRVPAIIKILTEFF-GKEPRRTMNASECVSRGCALQCAILSP---TFKVREFQVHESFPFS
BoHsp70-15a SDAGLSIEDVHMVEVVGSGSRVPAIIKILTEFF-GKEPRRTMNASECVSRGCALQCAILSP---TFKVREFQVHESFPFS
BoHsp70-15b ADAGLSVEDVHMVEVVGSGSRVPAIIKILTEFF-GKEPRRTMNASECVSRGCALQCAILSP---TFKVREFQVHESFPFS
BnA.Hsp70-15a SDAGLAIEDVHMVEVVGSGSRVPAIIKILTEFF-GKEPRRTMNASECVSRGCALQCAILSP---TFKVREFQVHESFPFS
BnA.Hsp70-15b ADAGLSVEDVHMVEVVGSGSRVPAIIKILTEFF-GKEPRRTMNASECVSRGCALQCAILSP---TFKVREFQVHESFPFS
BnC.Hsp70-15c SDAGLSIEDVHMVEVVGSGSRVPAIIKILTEFF-GKEPRRTMNASECVSRGCALQCAILSP---TFKVREFQVHESFPFS
BnC.Hsp70-15d ADAGLSVEDVHMVEVVGSGSRVPAIIKILTEFF-GKEPRRTMNASECVSRGCALQCAILSP---TFKVREFQVHESFPFS
BrHsp70-16 ADSGLSLDQIHSVELVGSGSRIPAISKMLSSLF-KRELGRTVNASECVARGCALQCAMLSP---IFRVRDYEVQDSFPFS
BoHsp70-16 ADSGLSLDQIHSVELVGSGSRIPAISKMLSSLF-KRELGRTVNASECVARGCALQCAMLSP---IFRVRDYEVQDSFPFS
BnC.Hsp70-16a ADSGLSLDQIHSVELVGSGSRIPAISKMLSSLF-KRELGRTVNASECVARGCALQCAMLSP---IFRVRDYEVQDSFPFS
BnA.Hsp70-16b ADSGLSLDQIHSVELVGSGSRIPAISKMLSSLF-KRELGRTVNASECVARGCALQCAMLSP---IFRVRDYEVQDSFPFS
BrHsp70-17a KHSGLKMDDIYAVELIGGATRVPKLQSTIQEFIGKQDLDKHLDADEAIVLGSALHAANLS--DGIKLKRRLGIVDGSPYG
BrHsp70-17b KHSGLKIDDIYAVELIGGATRVPKLQSTIQEFIGKQDLDKHLDADEAIVLGSALHAANLS--DGIKLKRRLGIIDGSPYG
BoHsp70-17a MHSGLKMDDIYAVELIGGATRVPKLQSTIQEFIGKQDLDKHLDADEAIVLGSALHAANLS--DGIKLKRRLGIVDGSPYG
BoHsp70-17b KHSGLKIDDIYAVELIGGATRVPKLQSTIQEFIGKEDLDKHLDADEAIVLGSALHAANLS--DGIKLKRRLGIVDGSPYG
BnA.Hsp70-17a KHSGLKMDDIYAVELIGGATRVPKLQSTIQEFIGKQDLDKHLDADEAIVLGSALHAANLS--DGIKLKRRLGIVDGSPYG
BnC.Hsp70-17b KHSGLKMDDIYAVELIGGATRVPKLQSTIQEFIGKQDLDKHLDADEAIVLGSALHAANLS--DGIKLKRRLGIVDGSPYG
BnC.Hsp70-17c KHSGLKIDDIYAVELIGGATRVPKLQSTIQEFIGKQDLDKHLDADEAIVLGSALYAANLS--DGIKLKRRLGIVDGSPYG
BnA.Hsp70-17d KHSGLKIDDIYAVELIGGATRVPKLQSTIQEFIGKQDLDKHLDADEAIVLGSALHAANLS--DGIKLKRRLGISDGSPYG

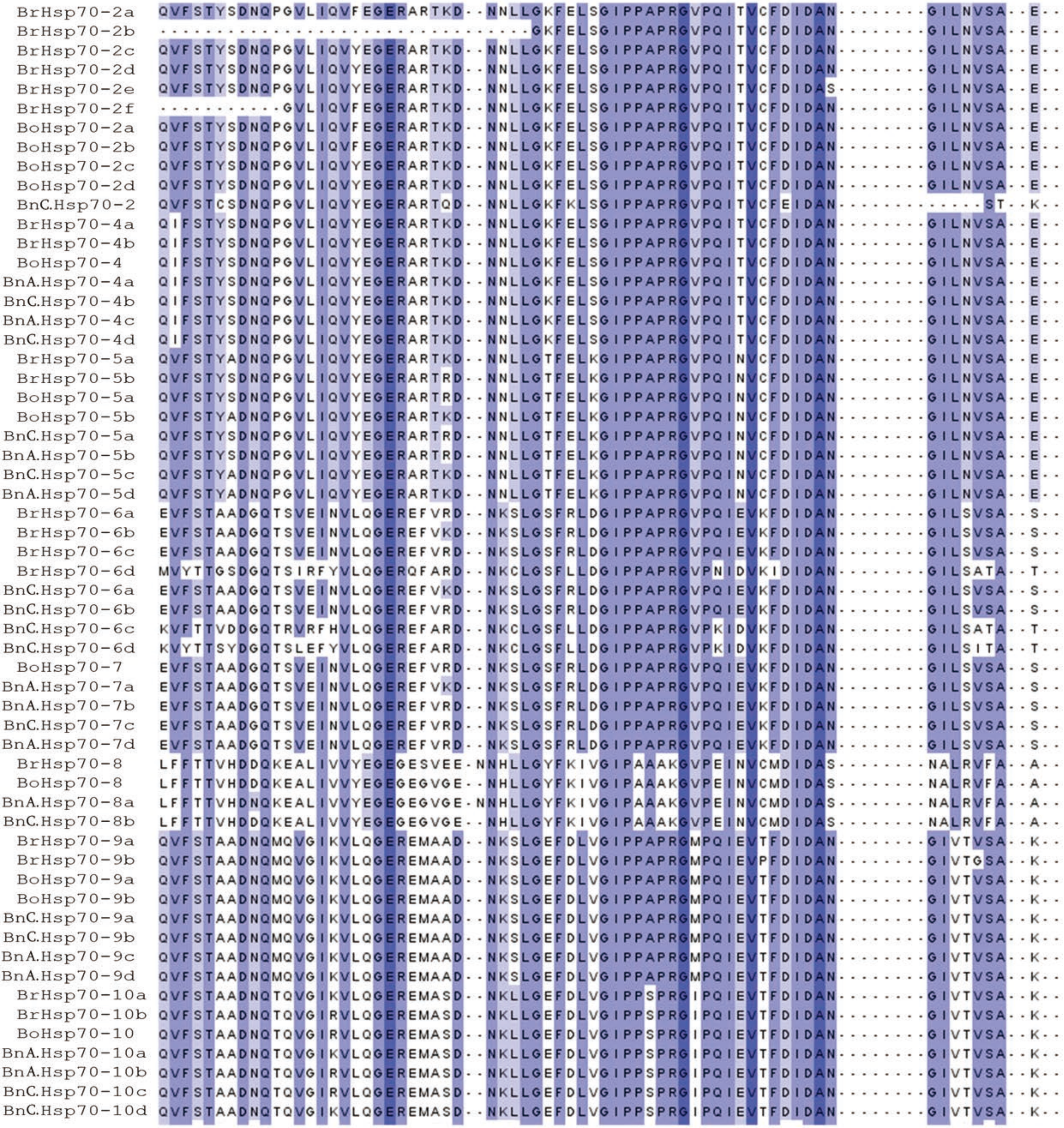
BrHsp70-2a QVFSTYSDNQPGVLIQVFEGERARTKD--NNLLGKFELSGIPPAPRGVPQITVCFDIDAN--------GILNVSA--E--
BrHsp70-2b ---------------------------------GKFELSGIPPAPRGVPQITVCFDIDAN--------GILNVSA--E--
BrHsp70-2c QVFSTYSDNQPGVLIQVYEGERARTKD--NNLLGKFELSGIPPAPRGVPQITVCFDIDAN--------GILNVSA--E--
BrHsp70-2d QVFSTYSDNQPGVLIQVYEGERARTKD--NNLLGKFELSGIPPAPRGVPQITVCFDIDAN--------GILNVSA--E--
BrHsp70-2e QVFSTYSDNQPGVLIQVYEGERARTKD--NNLLGKFELSGIPPAPRGVPQITVCFDIDAS--------GILNVSA--E--
BrHsp70-2f -----------GVLIQVFEGERARTKD--NNLLGKFELSGIPPAPRGVPQITVCFDIDAN--------GILNVSA--E--
BoHsp70-2a QVFSTYSDNQPGVLIQVFEGERARTKD--NNLLGKFELSGIPPAPRGVPQITVCFDIDAN--------GILNVSA--E--
BoHsp70-2b QVFSTYSDNQPGVLIQVFEGERARTKD--NNLLGKFELSGIPPAPRGVPQITVCFDIDAN--------GILNVSA--E--
BoHsp70-2c QVFSTYSDNQPGVLIQVYEGERARTKD--NNLLGKFELSGIPPAPRGVPQITVCFDIDAN--------GILNVSA--E--
BoHsp70-2d QVFSTYSDNQPGVLIQVYEGERARTKD--NNLLGKFELSGIPPAPRGVPQITVCFDIDAN--------GILNVSA--E--
BnC.Hsp70-2 QVFSTCSDNQPGVLIQVYEGERARTQD--NNLLGKFKLSGIPPAPRGVPQITVCFEIDAN-------------ST--K--
BrHsp70-4a QIFSTYSDNQPGVLIQVYEGERARTKD--NNLLGKFELSGIPPAPRGVPQITVCFDIDAN--------GILNVSA--E--
BrHsp70-4b QIFSTYSDNQPGVLIQVYEGERARTKD--NNLLGKFELSGIPPAPRGVPQITVCFDIDAN--------GILNVSA--E--
BoHsp70-4 QIFSTYSDNQPGVLIQVYEGERARTKD--NNLLGKFELSGIPPAPRGVPQITVCFDIDAN--------GILNVSA--E--
BnA.Hsp70-4a QIFSTYSDNQPGVLIQVYEGERARTKD--NNLLGKFELSGIPPAPRGVPQITVCFDIDAN--------GILNVSA--E--
BnC.Hsp70-4b QIFSTYSDNQPGVLIQVYEGERARTKD--NNLLGKFELSGIPPAPRGVPQITVCFDIDAN--------GILNVSA--E--
BnA.Hsp70-4c QIFSTYSDNQPGVLIQVYEGERARTKD--NNLLGKFELSGIPPAPRGVPQITVCFDIDAN--------GILNVSA--E--
BnC.Hsp70-4d QIFSTYSDNQPGVLIQVYEGERARTKD--NNLLGKFELSGIPPAPRGVPQITVCFDIDAN--------GILNVSA--E--
BrHsp70-5a QVFSTYADNQPGVLIQVYEGERARTKD--NNLLGTFELKGIPPAPRGVPQINVCFDIDAN--------GILNVSA--E--
BrHsp70-5b QVFSTYSDNQPGVLIQVYEGERARTRD--NNLLGTFELKGIPPAPRGVPQINVCFDIDAN--------GILNVSA--E--
BoHsp70-5a QVFSTYSDNQPGVLIQVYEGERARTRD--NNLLGTFELKGIPPAPRGVPQINVCFDIDAN--------GILNVSA--E--
BoHsp70-5b QVFSTYADNQPGVLIQVYEGERARTKD--NNLLGTFELKGIPPAPRGVPQINVCFDIDAN--------GILNVSA--E--
BnC.Hsp70-5a QVFSTYSDNQPGVLIQVYEGERARTRD--NNLLGTFELKGIPPAPRGVPQINVCFDIDAN--------GILNVSA--E--
BnA.Hsp70-5b QVFSTYSDNQPGVLIQVYEGERARTRD--NNLLGTFELKGIPPAPRGVPQINVCFDIDAN--------GILNVSA--E--
BnC.Hsp70-5c QVFSTYADNQPGVLIQVYEGERARTKD--NNLLGTFELKGIPPAPRGVPQINVCFDIDAN--------GILNVSA--E--
BnA.Hsp70-5d QVFSTYADNQPGVLIQVYEGERARTKD--NNLLGTFELKGIPPAPRGVPQINVCFDIDAN--------GILNVSA--E--
BrHsp70-6a EVFSTAADGQTSVEINVLQGEREFVRD--NKSLGSFRLDGIPPAPRGVPQIEVKFDIDAN--------GILSVSA--S--
BrHsp70-6b EVFSTAADGQTSVEINVLQGEREFVKD--NKSLGSFRLDGIPPAPRGVPQIEVKFDIDAN--------GILSVSA--S--
BrHsp70-6c EVFSTAADGQTSVEINVLQGEREFVRD--NKSLGSFRLDGIPPAPRGVPQIEVKFDIDAN--------GILSVSA--S--
BrHsp70-6d MVYTTGSDGQTSIRFYVLQGERQFARD--NKCLGSFLLDGIPPAPRGVPNIDVKIDIDAN--------GILSATA--T--
BnC.Hsp70-6a EVFSTAADGQTSVEINVLQGEREFVKD--NKSLGSFRLDGIPPAPRGVPQIEVKFDIDAN--------GILSVSA--S--
BnC.Hsp70-6b EVFSTAADGQTSVEINVLQGEREFVRD--NKSLGSFRLDGIPPAPRGVPQIEVKFDIDAN--------GILSVSA--S--
BnC.Hsp70-6c KVFTTVDDGQTRVRFHVLQGEREFARD--NKCLGSFLLDGIPPAPRGVPKIDVKFDIDAN--------GILSATA--T--
BnC.Hsp70-6d KVYTTSYDGQTSLEFYVLQGEREFARD--NKCLGSFLLDGIPPAPRGVPKIDVKFDIDAN--------GILSITA--T--
BoHsp70-7 EVFSTAADGQTSVEINVLQGEREFVRD--NKSLGSFRLDGIPPAPRGVPQIEVKFDIDAN--------GILSVSA--S--
BnA.Hsp70-7a EVFSTAADGQTSVEINVLQGEREFVKD--NKSLGSFRLDGIPPAPRGVPQIEVKFDIDAN--------GILSVSA--S--
BnA.Hsp70-7b EVFSTAADGQTSVEINVLQGEREFVRD--NKSLGSFRLDGIPPAPRGVPQIEVKFDIDAN--------GILSVSA--S--
BnC.Hsp70-7c EVFSTAADGQTSVEINVLQGEREFVRD--NKSLGSFRLDGIPPAPRGVPQIEVKFDIDAN--------GILSVSA--S--
BnA.Hsp70-7d EVFSTAADGQTSVEINVLQGEREFVRD--NKSLGSFRLDGIPPAPRGVPQIEVKFDIDAN--------GILSVSA--S--
BrHsp70-8 LFFTTVHDDQKEALIVVYEGEGESVEE-NNHLLGYFKIVGIPAAAKGVPEINVCMDIDAS--------NALRVFA--A--
BoHsp70-8 LFFTTVHDDQKEALIVVYEGEGEGVGE--NHLLGYFKIVGIPAAAKGVPEINVCMDIDAS--------NALRVFA--A--
BnA.Hsp70-8a LFFTTVHDNQKEALIVVYEGEGEGVGE-NNHLLGYFKIVGIPAAAKGVPEINVCMDIDAS--------NALRVFA--A--
BnC.Hsp70-8b LFFTTVHDDQKEALIVVYEGEGEGVGE--NHLLGYFKIVGIPAAAKGVPEINVCMDIDAS--------NALRVFA--A--
BrHsp70-9a QVFSTAADNQMQVGIKVLQGEREMAAD--NKSLGEFDLVGIPPAPRGMPQIEVTFDIDAN--------GIVTVSA--K--
BrHsp70-9b QVFSTAADNQMQVGIKVLQGEREMAAD--NKSLGEFDLVGIPPAPRGMPQIEVPFDIDAN--------GIVTGSA--K--
BoHsp70-9a QVFSTAADNQMQVGIKVLQGEREMAAD--NKSLGEFDLVGIPPAPRGMPQIEVTFDIDAN--------GIVTVSA--K--
BoHsp70-9b QVFSTAADNQMQVGIKVLQGEREMAAD--NKSLGEFDLVGIPPAPRGMPQIEVTFDIDAN--------GIVTVSA--K--
BnC.Hsp70-9a QVFSTAADNQMQVGIKVLQGEREMAAD--NKSLGEFDLVGIPPAPRGMPQIEVTFDIDAN--------GIVTVSA--K--
BnC.Hsp70-9b QVFSTAADNQMQVGIKVLQGEREMAAD--NKSLGEFDLVGIPPAPRGMPQIEVTFDIDAN--------GIVTVSA--K--
BnA.Hsp70-9c QVFSTAADNQMQVGIKVLQGEREMAAD--NKSLGEFDLVGIPPAPRGMPQIEVTFDIDAN--------GIVTVSA--K--
BnA.Hsp70-9d QVFSTAADNQMQVGIKVLQGEREMAAD--NKSLGEFDLVGIPPAPRGMPQIEVTFDIDAN--------GIVTVSA--K--
BrHsp70-10a QVFSTAADNQTQVGIKVLQGEREMASD--NKLLGEFDLVGIPPSPRGIPQIEVTFDIDAN--------GIVTVSA--K--
BrHsp70-10b QVFSTAADNQTQVGIRVLQGEREMASD--NKLLGEFDLVGIPPSPRGIPQIEVTFDIDAN--------GIVTVSA--K--
BoHsp70-10 QVFSTAADNQTQVGIRVLQGEREMASD--NKLLGEFDLVGIPPSPRGIPQIEVTFDIDAN--------GIVTVSA--K--
BnA.Hsp70-10a QVFSTAADNQTQVGIKVLQGEREMASD--NKLLGEFDLVGIPPSPRGIPQIEVTFDIDAN--------GIVTVSA--K--
BnA.Hsp70-10b QVFSTAADNQTQVGIRVLQGEREMASD--NKLLGEFDLVGIPPSPRGIPQIEVTFDIDAN--------GIVTVSA--K--
BnC.Hsp70-10c QVFSTAADNQTQVGIRVLQGEREMASD--NKLLGEFDLVGIPPSPRGIPQIEVTFDIDAN--------GIVTVSA--K--
BnC.Hsp70-10d QVFSTAADNQTQVGIKVLQGEREMASD--NKLLGEFDLVGIPPSPRGIPQIEVTFDIDAN--------GIVTVSA--K--

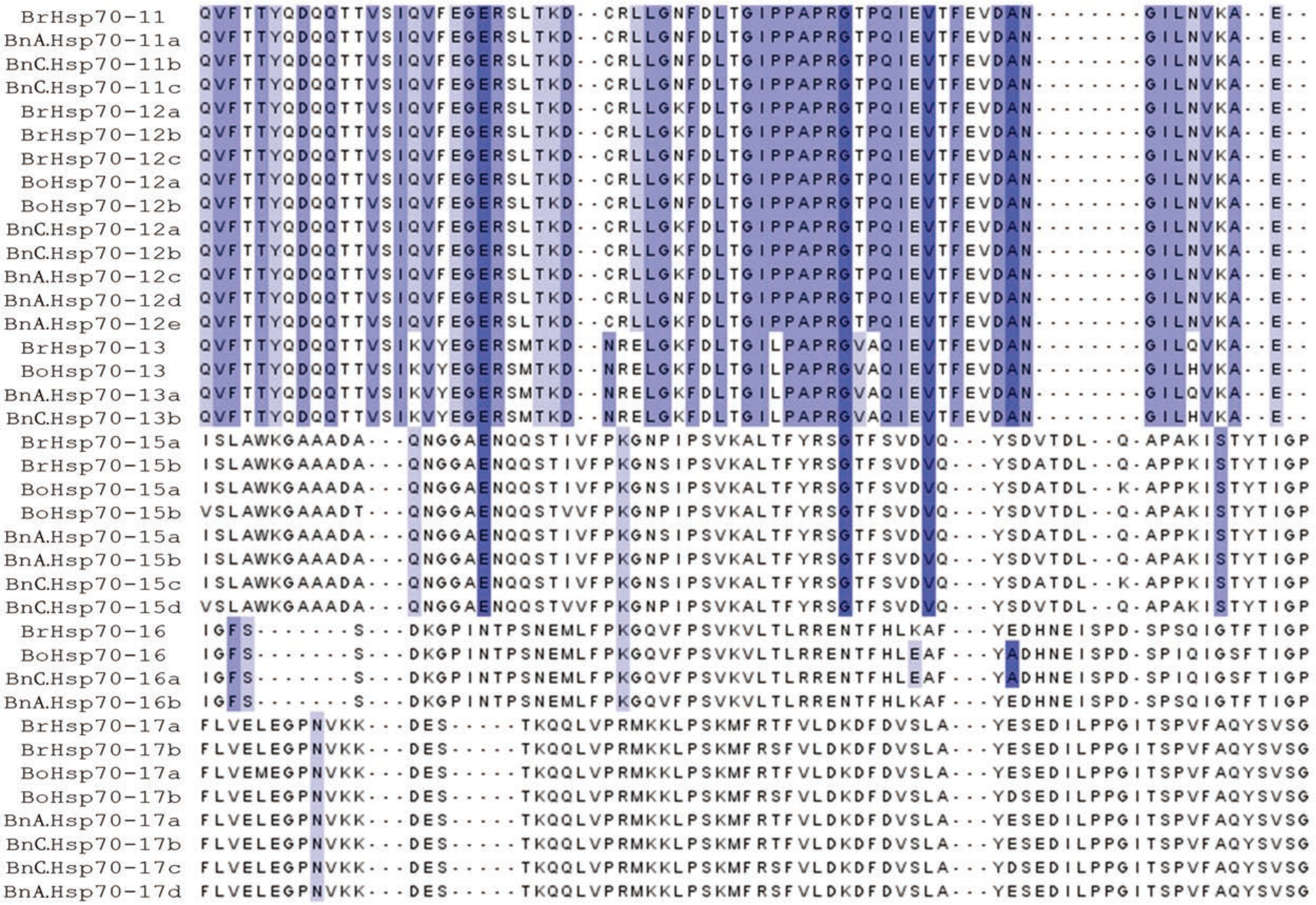
BrHsp70-11 QVFTTYQDQQTTVSIQVFEGERSLTKD--CRLLGNFDLTGIPPAPRGTPQIEVTFEVDAN--------GILNVKA--E--
BnA.Hsp70-11a QVFTTYQDQQTTVSIQVFEGERSLTKD--CRLLGNFDLTGIPPAPRGTPQIEVTFEVDAN--------GILNVKA--E--
BnC.Hsp70-11b QVFTTYQDQQTTVSIQVFEGERSLTKD--CRLLGNFDLTGIPPAPRGTPQIEVTFEVDAN--------GILNVKA--E--
BnC.Hsp70-11c QVFTTYQDQQTTVSIQVFEGERSLTKD--CRLLGNFDLTGIPPAPRGTPQIEVTFEVDAN--------GILNVKA--E--
BrHsp70-12a QVFTTYQDQQTTVSIQVFEGERSLTKD--CRLLGKFDLTGIPPAPRGTPQIEVTFEVDAN--------GILNVKA--E--
BrHsp70-12b QVFTTYQDQQTTVSIQVFEGERSLTKD--CRLLGKFDLTGIPPAPRGTPQIEVTFEVDAN--------GILNVKA--E--
BrHsp70-12c QVFTTYQDQQTTVSIQVFEGERSLTKD--CRLLGNFDLTGIPPAPRGTPQIEVTFEVDAN--------GILNVKA--E--
BoHsp70-12a QVFTTYQDQQTTVSIQVFEGERSLTKD--CRLLGKFDLTGIPPAPRGTPQIEVTFEVDAN--------GILNVKA--E--
BoHsp70-12b QVFTTYQDQQTTVSIQVFEGERSLTKD--CRLLGNFDLTGIPPAPRGTPQIEVTFEVDAN--------GILNVKA--E--
BnC.Hsp70-12a QVFTTYQDQQTTVSIQVFEGERSLTKD--CRLLGKFDLTGIPPAPRGTPQIEVTFEVDAN--------GILNVKA--E--
BnC.Hsp70-12b QVFTTYQDQQTTVSIQVFEGERSLTKD--CRLLGKFDLTGIPPAPRGTPQIEVTFEVDAN--------GILNVKA--E--
BnA.Hsp70-12c QVFTTYQDQQTTVSIQVFEGERSLTKD--CRLLGKFDLTGIPPAPRGTPQIEVTFEVDAN--------GILNVKA--E--
BnA.Hsp70-12d QVFTTYQDQQTTVSIQVFEGERSLTKD--CRLLGNFDLTGIPPAPRGTPQIEVTFEVDAN--------GILNVKA--E--
BnA.Hsp70-12e QVFTTYQDQQTTVSIQVFEGERSLTKD--CRLLGKFDLTGIPPAPRGTPQIEVTFEVDAN--------GILNVKA--E--
BrHsp70-13 QVFTTYQDQQTTVSIKVYEGERSMTKD--NRELGKFDLTGILPAPRGVAQIEVTFEVDAN--------GILQVKA--E--
BoHsp70-13 QVFTTYQDQQTTVSIKVYEGERSMTKD--NRELGKFDLTGILPAPRGVAQIEVTFEVDAN--------GILHVKA--E--
BnA.Hsp70-13a QVFTTYQDQQTTVSIKVYEGERSMTKD--NRELGKFDLTGILPAPRGVAQIEVTFEVDAN--------GILQVKA--E--
BnC.Hsp70-13b QVFTTYQDQQTTVSIKVYEGERSMTKD--NRELGKFDLTGILPAPRGVAQIEVTFEVDAN--------GILHVKA--E--
BrHsp70-15a ISLAWKGAAADA---QNGGAENQQSTIVFPKGNPIPSVKALTFYRSGTFSVDVQ---YSDVTDL--Q-APAKISTYTIGP
BrHsp70-15b ISLAWKGAAADA---QNGGAENQQSTIVFPKGNSIPSVKALTFYRSGTFSVDVQ---YSDATDL--Q-APPKISTYTIGP
BoHsp70-15a ISLAWKGAAADA---QNGGAENQQSTIVFPKGNSIPSVKALTFYRSGTFSVDVQ---YSDATDL--K-APPKISTYTIGP
BoHsp70-15b VSLAWKGAAADT---QNGGAENQQSTVVFPKGNPIPSVKALTFYRSGTFSVDVQ---YSDVTDL--Q-APAKISTYTIGP
BnA.Hsp70-15a ISLAWKGAAADA---QNGGAENQQSTIVFPKGNSIPSVKALTFYRSGTFSVDVQ---YSDATDL--Q-APPKISTYTIGP
BnA.Hsp70-15b ISLAWKGAAADA---QNGGAENQQSTIVFPKGNPIPSVKALTFYRSGTFSVDVQ---YSDVTDL--Q-APAKISTYTIGP
BnC.Hsp70-15c ISLAWKGAAADA---QNGGAENQQSTIVFPKGNSIPSVKALTFYRSGTFSVDVQ---YSDATDL--K-APPKISTYTIGP
BnC.Hsp70-15d VSLAWKGAAADA---QNGGAENQQSTVVFPKGNPIPSVKALTFYRSGTFSVDVQ---YSDVTDL--Q-APAKISTYTIGP
BrHsp70-16 IGFS-------S---DKGPINTPSNEMLFPKGQVFPSVKVLTLRRENTFHLKAF---YEDHNEISPD-SPSQIGTFTIGP
BoHsp70-16 IGFS-------S---DKGPINTPSNEMLFPKGQVFPSVKVLTLRRENTFHLEAF---YADHNEISPD-SPIQIGSFTIGP
BnC.Hsp70-16a IGFS-------S---DKGPINTPSNEMLFPKGQVFPSVKVLTLRRENTFHLEAF---YADHNEISPD-SPIQIGSFTIGP
BnA.Hsp70-16b IGFS-------S---DKGPINTPSNEMLFPKGQVFPSVKVLTLRRENTFHLKAF---YEDHNEISPD-SPSQIGTFTIGP
BrHsp70-17a FLVELEGPNVKK---DES-----TKQQLVPRMKKLPSKMFRTFVLDKDFDVSLA---YESEDILPPGITSPVFAQYSVSG
BrHsp70-17b FLVELEGPNVKK---DES-----TKQQLVPRMKKLPSKMFRSFVLDKDFDVSLA---YESEDILPPGITSPVFAQYSVSG
BoHsp70-17a FLVEMEGPNVKK---DES-----TKQQLVPRMKKLPSKMFRTFVLDKDFDVSLA---YESEDILPPGITSPVFAQYSVSG
BoHsp70-17b FLVELEGPNVKK---DES-----TKQQLVPRMKKLPSKMFRSFVLDKDFDVSLA---YDSEDILPPGITSPVFAQYSVSG
BnA.Hsp70-17a FLVELEGPNVKK---DES-----TKQQLVPRMKKLPSKMFRTFVLDKDFDVSLA---YESEDILPPGITSPVFAQYSVSG
BnC.Hsp70-17b FLVELEGPNVKK---DES-----TKQQLVPRMKKLPSKMFRTFVLDKDFDVSLA---YESEDILPPGITSPVFAQYSVSG
BnC.Hsp70-17c FLVELEGPNVKK---DES-----TKQQLVPRMKKLPSKMFRSFVLDKDFDVSLA---YDSEDILPPGITSPVFAQYSVSG
BnA.Hsp70-17d FLVELEGPNVKK---DES-----TKQQLVPRMKKLPSKMFRSFVLDKDFDVSLA---YESEDILPPGITSPVFAQYSVSG

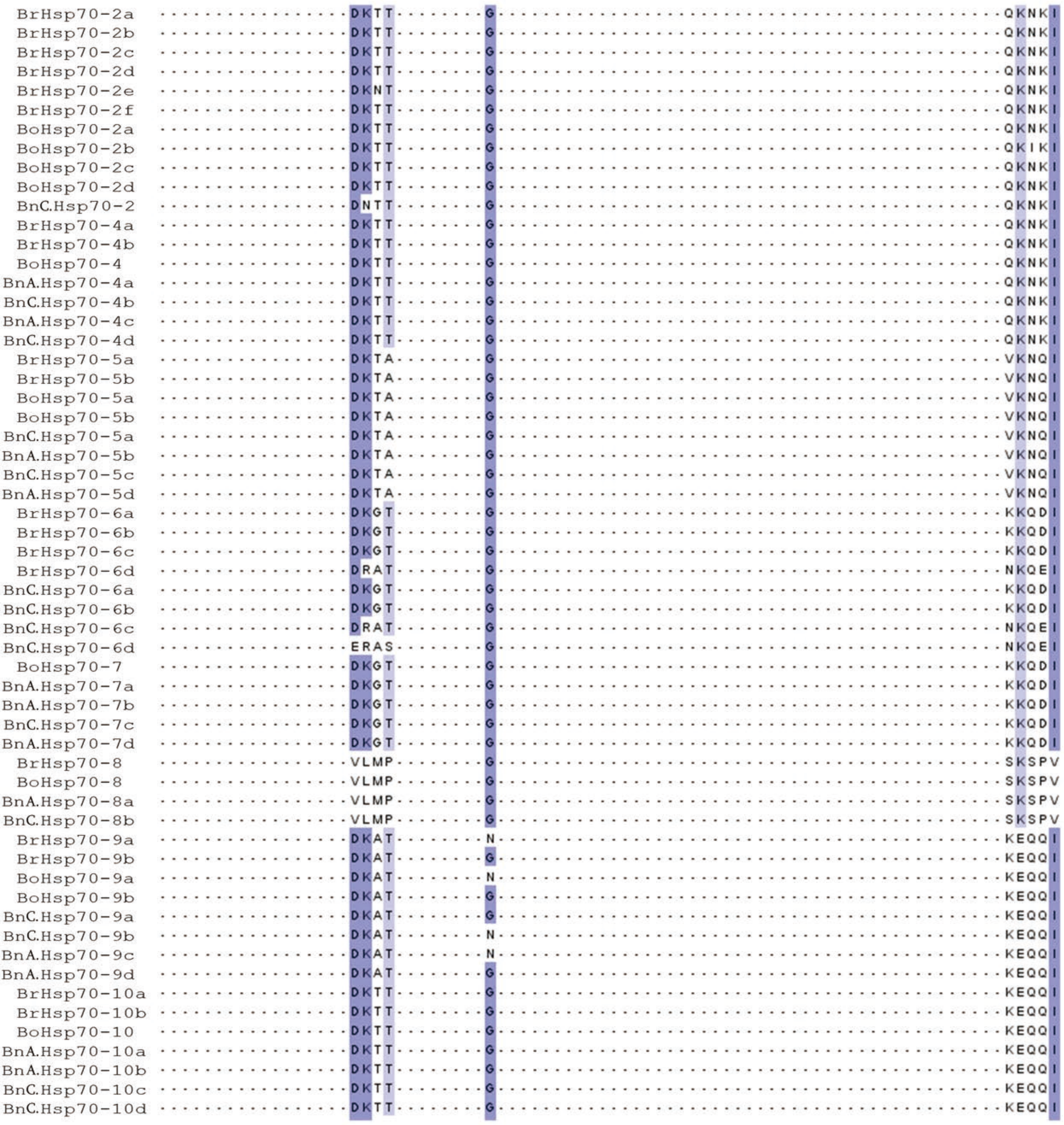
BrHsp70-2a DKTT G QKNKI
BrHsp70-2b DKTT G QKNKI
BrHsp70-2c DKTT G QKNKI
BrHsp70-2d DKTT G QKNKI
BrHsp70-2e DKNT G QKNKI
BrHsp70-2f DKTT G QKNKI
BoHsp70-2a DKTT G QKNKI
BoHsp70-2b DKTT G QKIKI
BoHsp70-2c DKTT G QKNKI
BoHsp70-2d DKTT G QKNKI
BnC.Hsp70-2 DNTT G QKNKI
BrHsp70-4a DKTT G QKNKI
BrHsp70-4b DKTT G QKNKI
BoHsp70-4 DKTT G QKNKI
BnA.Hsp70-4a DKTT G QKNKI
BnC.Hsp70-4b DKTT G QKNKI
BnA.Hsp70-4c DKTT G QKNKI
BnC.Hsp70-4d DKTT G QKNKI
BrHsp70-5a DKTA G VKNQI
BrHsp70-5b DKTA G VKNQI
BoHsp70-5a DKTA G VKNQI
BoHsp70-5b DKTA G VKNQI
BnC.Hsp70-5a DKTA G VKNQI
BnA.Hsp70-5b DKTA G VKNQI
BnC.Hsp70-5c DKTA G VKNQI
BnA.Hsp70-5d DKTA G VKNQI
BrHsp70-6a DKGT G KKQDI
BrHsp70-6b DKGT G KKQDI
BrHsp70-6c DKGT G KKQDI
BrHsp70-6d DRAT G NKQEI
BnC.Hsp70-6a DKGT G KKQDI
BnC.Hsp70-6b DKGT G KKQDI
BnC.Hsp70-6c DRAT G NKQEI
BnC.Hsp70-6d ERAS G NKQEI
BoHsp70-7 DKGT G KKQDI
BnA.Hsp70-7a DKGT G KKQDI
BnA.Hsp70-7b DKGT G KKQDI
BnC.Hsp70-7c DKGT G KKQDI
BnA.Hsp70-7d DKGT G KKQDI
BrHsp70-8 VLMP G SKSPV
BoHsp70-8 VLMP G SKSPV
BnA.Hsp70-8a VLMP G SKSPV
BnC.Hsp70-8b VLMP G SKSPV
BrHsp70-9a DKAT N KEQQI
BrHsp70-9b DKAT G KEQQI
BoHsp70-9a DKAT N KEQQI
BoHsp70-9b DKAT G KEQQI
BnC.Hsp70-9a DKAT G KEQQI
BnC.Hsp70-9b DKAT N KEQQI
BnA.Hsp70-9c DKAT N KEQQI
BnA.Hsp70-9d DKAT G KEQQI
BrHsp70-10a DKTT G KEQQI
BrHsp70-10b DKTT G KEQQI
BoHsp70-10 DKTT G KEQQI
BnA.Hsp70-10a DKTT G KEQQI
BnA.Hsp70-10b DKTT G KEQQI
BnC.Hsp70-10c DKTT G KEQQI
BnC.Hsp70-10d DKTT G KEQQI

```
BrHsp70-11      -----------------DKAS--------G---------------------------------------------KSEKI
BnA.Hsp70-11a   -----------------DKAS-----------------------------------------------------------
BnC.Hsp70-11b   -----------------DKAS-----------------------------------------------------------
BnC.Hsp70-11c   -----------------DKAS-----------------------------------------------------------
BrHsp70-12a     -----------------DKAS--------G---------------------------------------------KSEKI
BrHsp70-12b     -----------------DKAS--------G---------------------------------------------KSEKI
BrHsp70-12c     -----------------DRAS-----------------------------------------------------------
BoHsp70-12a     -----------------DKAS--------G---------------------------------------------KSEKI
BoHsp70-12b     -----------------DKAS--------G---------------------------------------------KSEKI
BnC.Hsp70-12a   -----------------DKAS--------G---------------------------------------------KSEKI
BnC.Hsp70-12b   -----------------DKAS-----------------------------------------------------------
BnA.Hsp70-12c   -----------------DKAS-----------------------------------------------------------
BnA.Hsp70-12d   -----------------DKAS-----------------------------------------------------------
BnA.Hsp70-12e   -----------------DKAS-----------------------------------------------------------
BrHsp70-13      -----------------DKVA--------K---------------------------------------------TSQSI
BoHsp70-13      -----------------DKVA--------K---------------------------------------------TSQTI
BnA.Hsp70-13a   -----------------DKVA--------K---------------------------------------------TSQSI
BnC.Hsp70-13b   -----------------DKVA--------K---------------------------------------------TSQTI
BrHsp70-15a     FQSSKGERAKVKVKVRLTLH--------GIVSVES--ATLLEEEEVEVPVTTEQMDTDKAS--------GETDVNMQDAK
BrHsp70-15b     FQSSKGERAKLKVKVRLTLH--------GIVSVES--ATLLEEEEVEVKVTAEQMDTDKAS--------GESDVNMQDAK
BoHsp70-15a     FQSSKGERAKLKVKVRLTLH--------GIVSVES--ATLLEEEEVEVKVTAEQMDTDKAS--------GESDVNMQDAK
BoHsp70-15b     FQSSKGERAKVKVKVRLTLH--------GIVSVES--ATLLEEEEVDVPVTTEQMDTDKAS--------GETDVNMQDAK
BnA.Hsp70-15a   FQSSKGERAKLKVKVRLTLH--------GIVSVES--ATLLEEEEVEVKVTTEQMDTDKAS--------GESDVNMQDAK
BnA.Hsp70-15b   FQSSKGERAKVKVKVRLTLH--------GIVSVES--ATLLEEEEVEVPVTTEQMDTDKAS--------GETDVNMQDAK
BnC.Hsp70-15c   FQSSKGERAKLKVKVRLTLH--------GIVSVES--ATLLEEEEVEVKVTAEQMDTDKAS--------GESDVNMQDAK
BnC.Hsp70-15d   FQSSKGERAKVKVKVRLTLH--------GIVSVES--ATLLEEEEVEVPVTTEQMDTDKAS--------GETDVNMQDAK
BrHsp70-16      FQSSHGEAARVKVRVQLNLH--------GIVTIDS--ASLIEDPK------------ENTT--------SEETV----SE
BoHsp70-16      FQSSHGQAARVKVRVQLNLH--------GIVTIDS--ASLIEDPK------------ENTT--------SEETV----SE
BnC.Hsp70-16a   FQSSHGQAARVKVRVQLNLH--------GIVTIDS--ASLIEDPK------------ENTT--------SEETV----SE
BnA.Hsp70-16b   FQSSHGEAARVKVRVQLNLH--------GIVTIDS--ASLIEDPK------------ENTT--------SEETV----SE
BrHsp70-17a     LTDATEKYSSRNLSAPIKANLHFSLSRSGVLSLDRGDAVIEITEWVEVPKKNVTIESNTTSTTGNASTGAASDENSQENK
BrHsp70-17b     LADATEKYSSRNLSAPIKANLHFSLSRSGILSLDRGDAVIEITEWVEVPKKNVTVDNNTTSTTGNASTGAPSDENLQENK
BoHsp70-17a     LTDATEKYSSRNLSAPIKANLHFSLSRSGILSLDRGDAVIEITEWVEVPKKNVTIESNTTSTTGNASTGAASDENSQENK
BoHsp70-17b     FAVATEKYSSRNLSAPIKANLHFSLSRSGILSLDRGDAVIEITEWVEVPKKNVTVDNNTTSTTGNASTGAPSDENLQENK
BnA.Hsp70-17a   LTDATEKYSSRNLSAPIKANLHFSLSRSGILSLDRGDAVIEITEWVEVPKKNVTIESNTTSTTGNASTGAASDENSQENK
BnC.Hsp70-17b   LTDATEKYSSRNLSAPIKANLHFSLSRSGILSLDRGDAVIEITEWVEVPKKNVTIESNTTSTTGNASTGAASDENSQENK
BnC.Hsp70-17c   FAVATEKYSSRNLSAPIKANLHFSLSRSGILSLDRGDAVIEITEWVEVPKKNVTVDNNTTSTTGNASTGAPSDENLQENK
BnA.Hsp70-17d   LADATEKYSSRNLSAPIKANLHFSLSRSGILSLDRGDAVIEITEWVEVPKKNVTVDNNTTSTTGNASTGAPSDENLQENK
```

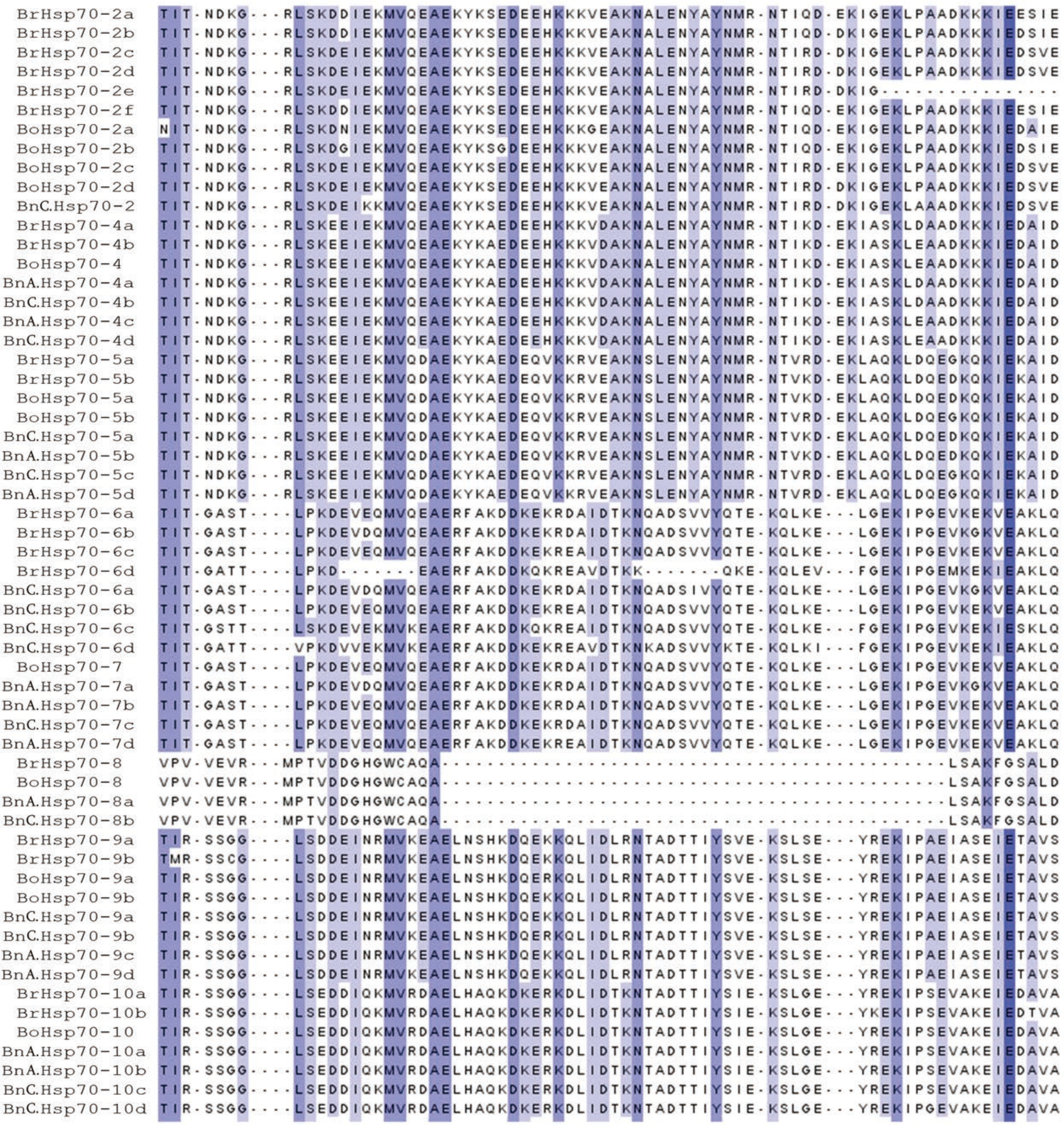
BrHsp70-2a TIT-NDKG---RLSKDDIEKMVQEAEKYKSEDEEHKKKVEAKNALENYAYNMR-NTIQD-EKIGEKLPAADKKKIEESIE
BrHsp70-2b TIT-NDKG---RLSKDDIEKMVQEAEKYKSEDEEHKKKVEAKNALENYAYNMR-NTIQD-DKIGEKLPAADKKKIEDSIE
BrHsp70-2c TIT-NDKG---RLSKDEIEKMVQEAEKYKSEDEEHKKKVEAKNALENYAYNMR-NTIRD-DKIGEKLPAADKKKIEDSVE
BrHsp70-2d TIT-NDKG---RLSKDEIEKMVQEAEKYKSEDEEHKKKVEAKNALENYAYNMR-NTIRD-DKIGEKLPAADKKKIEDSVE
BrHsp70-2e TIT-NDKG---RLSKDEIEKMVQEAEKYKSEDEEHKKKVEAKNALENYAYNMR-NTIRD-DKIG---------------
BrHsp70-2f TIT-NDKG---RLSKDDIEKMVQEAEKYKSEDEEHKKKVEAKNALENYAYNMR-NTIQD-EKIGEKLPAADKKKIEESIE
BoHsp70-2a NIT-NDKG---RLSKDNIEKMVQEAEKYKSEDEEHKKKGEAKNALENYAYNMR-NTIQD-EKIGEKLPAADKKKIEDAIE
BoHsp70-2b TIT-NDKG---RLSKDGIEKMVQEAEKYKSGDEEHKKKVEAKNALENYAYNMR-NTIQD-EKIGEKLPAADKKKIEDSIE
BoHsp70-2c TIT-NDKG---RLSKDEIEKMVQEAEKYKSEDEEHKKKVEAKNALENYAYNMR-NTIRD-EKIGEKLPAADKKKIEDSVE
BoHsp70-2d TIT-NDKG---RLSKDEIEKMVQEAEKYKSEDEEHKKKVEAKNALENYAYNMR-NTIRD-DKIGEKLPAADKKKIEDSVE
BnC.Hsp70-2 TIT-NDKG---RLSKDEIKKMVQEAEKYKSEDEEHKKKVEAKNALENYAYNMR-NTIRD-DKIGEKLAAADKKKIEDSVE
BrHsp70-4a TIT-NDKG---RLSKEEIEKMVQEAEKYKAEDEEHKKKVDAKNALENYAYNMR-NTIKD-EKIASKLDAADKKKIEDAID
BrHsp70-4b TIT-NDKG---RLSKEEIEKMVQEAEKYKAEDEEHKKKVDAKNALENYAYNMR-NTIKD-EKIASKLEAADKKKIEDAID
BoHsp70-4 TIT-NDKG---RLSKEEIEKMVQEAEKYKAEDEEHKKKVDAKNALENYAYNMR-NTIKD-EKIASKLEAADKKKIEDAID
BnA.Hsp70-4a TIT-NDKG---RLSKEEIEKMVQEAEKYKAEDEEHKKKVDAKNALENYAYNMR-NTIKD-EKIASKLDAADKKKIEDAID
BnC.Hsp70-4b TIT-NDKG---RLSKEEIEKMVQEAEKYKAEDEEHKKKVDAKNALENYAYNMR-NTIKD-EKIASKLDAADKKKIEDAID
BnA.Hsp70-4c TIT-NDKG---RLSKEEIEKMVQEAEKYKAEDEEHKKKVDAKNALENYAYNMR-NTIKD-EKIASKLEAADKKKIEDAID
BnC.Hsp70-4d TIT-NDKG---RLSKEEIEKMVQEAEKYKAEDEEHKKKVDAKNALENYAYNMR-NTIKD-EKIASKLEAADKKKIEDAID
BrHsp70-5a TIT-NDKG---RLSKEEIEKMVQDAEKYKAEDEQVKKRVEAKNSLENYAYNMR-NTVRD-EKLAQKLDQEGKQKIEKAID
BrHsp70-5b TIT-NDKG---RLSKEEIEKMVQDAEKYKAEDEQVKKRVEAKNSLENYAYNMR-NTVKD-EKLAQKLDQEDKQKIEKAID
BoHsp70-5a TIT-NDKG---RLSKEEIEKMVQDAEKYKAEDEQVKKRVEAKNSLENYAYNMR-NTVKD-EKLAQKLDQEDKQKIEKAID
BoHsp70-5b TIT-NDKG---RLSKEEIEKMVQDAEKYKAEDEQVKKRVEAKNSLENYAYNMR-NTVRD-EKLAQKLDQEGKQKIEKAID
BnC.Hsp70-5a TIT-NDKG---RLSKEEIEKMVQDAEKYKAEDEQVKKRVEAKNSLENYAYNMR-NTVKD-EKLAQKLDQEDKQKIEKAID
BnA.Hsp70-5b TIT-NDKG---RLSKEEIEKMVQDAEKYKAEDEQVKKRVEAKNSLENYAYNMR-NTVKD-EKLAQKLDQEDKQKIEKAID
BnC.Hsp70-5c TIT-NDKG---RLSKEEIEKMVQDAEKYKAEDEQVKKRVEAKNSLENYAYNMR-NTVRD-EKLAQKLDQEGKQKIEKAID
BnA.Hsp70-5d TIT-NDKG---RLSKEEIEKMVQDAEKYKAEDEQVKKRVEAKNSLENYAYNMR-NTVRD-EKLAQKLDQEGKQKIEKAID
BrHsp70-6a TIT-GAST----LPKDEVEQMVQEAERFAKDDKEKRDAIDTKNQADSVVYQTE-KQLKE---LGEKIPGEVKEKVEAKLQ
BrHsp70-6b TIT-GAST----LPKDEVDQMVQEAERFAKDDKEKRDAIDTKNQADSVVYQTE-KQLKE---LGEKIPGEVKGKVEAKLQ
BrHsp70-6c TIT-GAST----LPKDEVEQMVQEAERFAKDDKEKREAIDTKNQADSVVYQTE-KQLKE---LGEKIPGEVKEKVEAKLQ
BrHsp70-6d TIT-GATT----LPKD-------EAERFAKDDKQKREAVDTKK-------QKE-KQLEV---FGEKIPGEMKEKIEAKLQ
BnC.Hsp70-6a TIT-GAST----LPKDEVDQMVQEAERFAKDDKEKRDAIDTKNQADSIVYQTE-KQLKE---LGEKIPGEVKGKVEAKLQ
BnC.Hsp70-6b TIT-GAST----LPKDEVEQMVQEAERFAKDDKEKREAIDTKNQADSVVYQTE-KQLKE---LGEKIPGEVKEKVEAKLQ
BnC.Hsp70-6c TIT-GSTT----LSKDEVEKMVKEAERFAKDDKQKREAIDTKNQADSVVYQTE-KQLKE---FGEKIPGEVKEKIESKLQ
BnC.Hsp70-6d TIT-GATT---VPKDVVEKMVKEAERFAKDDKEKREAVDTKNKADSVVYKTE-KQLKI---FGEKIPGEVKEKIEAKLQ
BoHsp70-7 TIT-GAST----LPKDEVEQMVQEAERFAKDDKEKRDAIDTKNQADSVVYQTE-KQLKE---LGEKIPGEVKEKVEAKLQ
BnA.Hsp70-7a TIT-GAST----LPKDEVDQMVQEAERFAKDDKEKRDAIDTKNQADSVVYQTE-KQLKE---LGEKIPGEVKGKVEAKLQ
BnA.Hsp70-7b TIT-GAST----LPKDEVEQMVQEAERFAKDDKEKRDAIDTKNQADSVVYQTE-KQLKE---LGEKIPGEVKEKVEAKLQ
BnC.Hsp70-7c TIT-GAST----LPKDEVEQMVQEAERFAKDDKEKRDAIDTKNQADSVVYQTE-KQLKE---LGEKIPGEVKEKVEAKLQ
BnA.Hsp70-7d TIT-GAST----LPKDEVEQMVQEAERFAKDDKEKREAIDTKNQADSVVYQTE-KQLKE---LGEKIPGEVKEKVEAKLQ
BrHsp70-8 VPV-VEVR---MPTVDDGHGWCAQA-----------------------------------LSAKFGSALD
BoHsp70-8 VPV-VEVR---MPTVDDGHGWCAQA-----------------------------------LSAKFGSALD
BnA.Hsp70-8a VPV-VEVR---MPTVDDGHGWCAQA-----------------------------------LSAKFGSALD
BnC.Hsp70-8b VPV-VEVR---MPTVDDGHGWCAQA-----------------------------------LSAKFGSALD
BrHsp70-9a TIR-SSGG----LSDDEINRMVKEAELNSHKDQEKKQLIDLRNTADTTIYSVE-KSLSE---YREKIPAEIASEIETAVS
BrHsp70-9b TMR-SSCG----LSDDEINRMVKEAELNSHKDQEKKQLIDLRNTADTTIYSVE-KSLSE---YREKIPAEIASEIETAVS
BoHsp70-9a TIR-SSGG----LSDDEINRMVKEAELNSHKDQERKQLIDLRNTADTTIYSVE-KSLSE---YREKIPAEIASEIETAVS
BoHsp70-9b TIR-SSGG----LSDDEINRMVKEAELNSHKDQEKKQLIDLRNTADTTIYSVE-KSLSE---YREKIPAEIASEIETAVS
BnC.Hsp70-9a TIR-SSGG----LSDDEINRMVKEAELNSHKDQEKKQLIDLRNTADTTIYSVE-KSLSE---YREKIPAEIASEIETAVS
BnC.Hsp70-9b TIR-SSGG----LSDDEINRMVKEAELNSHKDQERKQLIDLRNTADTTIYSVE-KSLSE---YREKIPAEIASEIETAVS
BnA.Hsp70-9c TIR-SSGG----LSDDEINRMVKEAELNSHKDQEKKQLIDLRNTADTTIYSVE-KSLSE---YREKIPAEIASEIETAVS
BnA.Hsp70-9d TIR-SSGG----LSDDEINRMVKEAELNSHKDQEKKQLIDLRNTADTTIYSVE-KSLSE---YREKIPAEIASEIETAVS
BrHsp70-10a TIR-SSGG----LSEDDIQKMVRDAELHAQKDKERKDLIDTKNTADTTIYSIE-KSLGE---YREKIPSEVAKEIEDAVA
BrHsp70-10b TIR-SSGG----LSEDDIQKMVRDAELHAQKDKERKDLIDTKNTADTTIYSIE-KSLGE---YKEKIPSEVAKEIEDTVA
BoHsp70-10 TIR-SSGG----LSEDDIQKMVRDAELHAQKDKERKDLIDTKNTADTTIYSIE-KSLGE---YREKIPSEVAKEIEDAVA
BnA.Hsp70-10a TIR-SSGG----LSEDDIQKMVRDAELHAQKDKERKDLIDTKNTADTTIYSIE-KSLGE---YREKIPSEVAKEIEDAVA
BnA.Hsp70-10b TIR-SSGG----LSEDDIQKMVRDAELHAQKDKERKDLIDTKNTADTTIYSIE-KSLGE---YREKIPSEVAKEIEDAVA
BnC.Hsp70-10c TIR-SSGG----LSEDDIQKMVRDAELHAQKDKERKDLIDTKNTADTTIYSIE-KSLGE---YREKIPSEVAKEIEDAVA
BnC.Hsp70-10d TIR-SSGG----LSEDDIQKMVRDAELHAQKDKERKDLIDTKNTADTTIYSIE-KSLGE---YREKIPGEVAKEIEDAVA

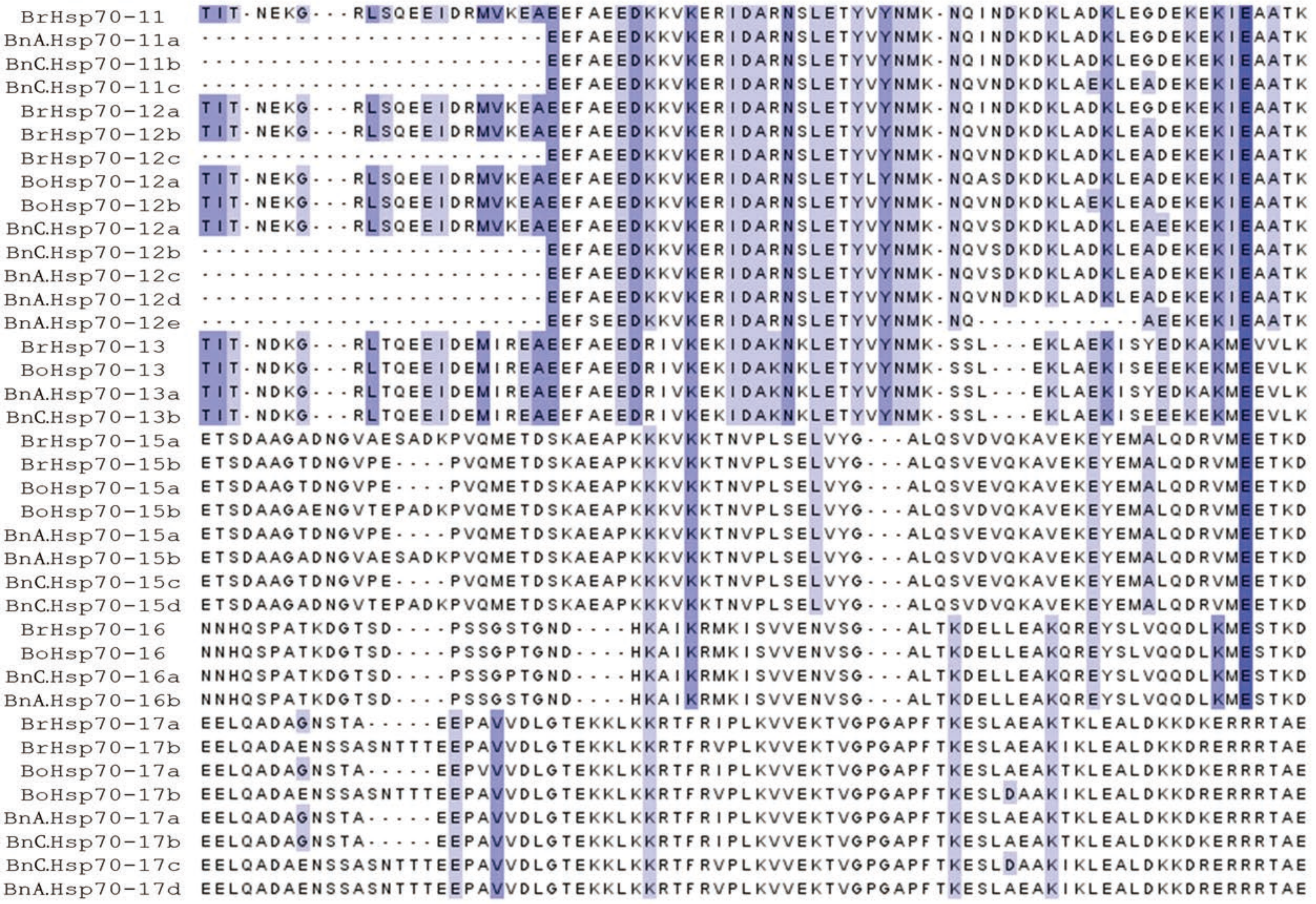
BrHsp70-11
BnA.Hsp70-11a
BnC.Hsp70-11b
BnC.Hsp70-11c
BrHsp70-12a
BrHsp70-12b
BrHsp70-12c
BoHsp70-12a
BoHsp70-12b
BnC.Hsp70-12a
BnC.Hsp70-12b
BnA.Hsp70-12c
BnA.Hsp70-12d
BnA.Hsp70-12e
BrHsp70-13
BoHsp70-13
BnA.Hsp70-13a
BnC.Hsp70-13b
BrHsp70-15a
BrHsp70-15b
BoHsp70-15a
BoHsp70-15b
BnA.Hsp70-15a
BnA.Hsp70-15b
BnC.Hsp70-15c
BnC.Hsp70-15d
BrHsp70-16
BoHsp70-16
BnC.Hsp70-16a
BnA.Hsp70-16b
BrHsp70-17a
BrHsp70-17b
BoHsp70-17a
BoHsp70-17b
BnA.Hsp70-17a
BnC.Hsp70-17b
BnC.Hsp70-17c
BnA.Hsp70-17d

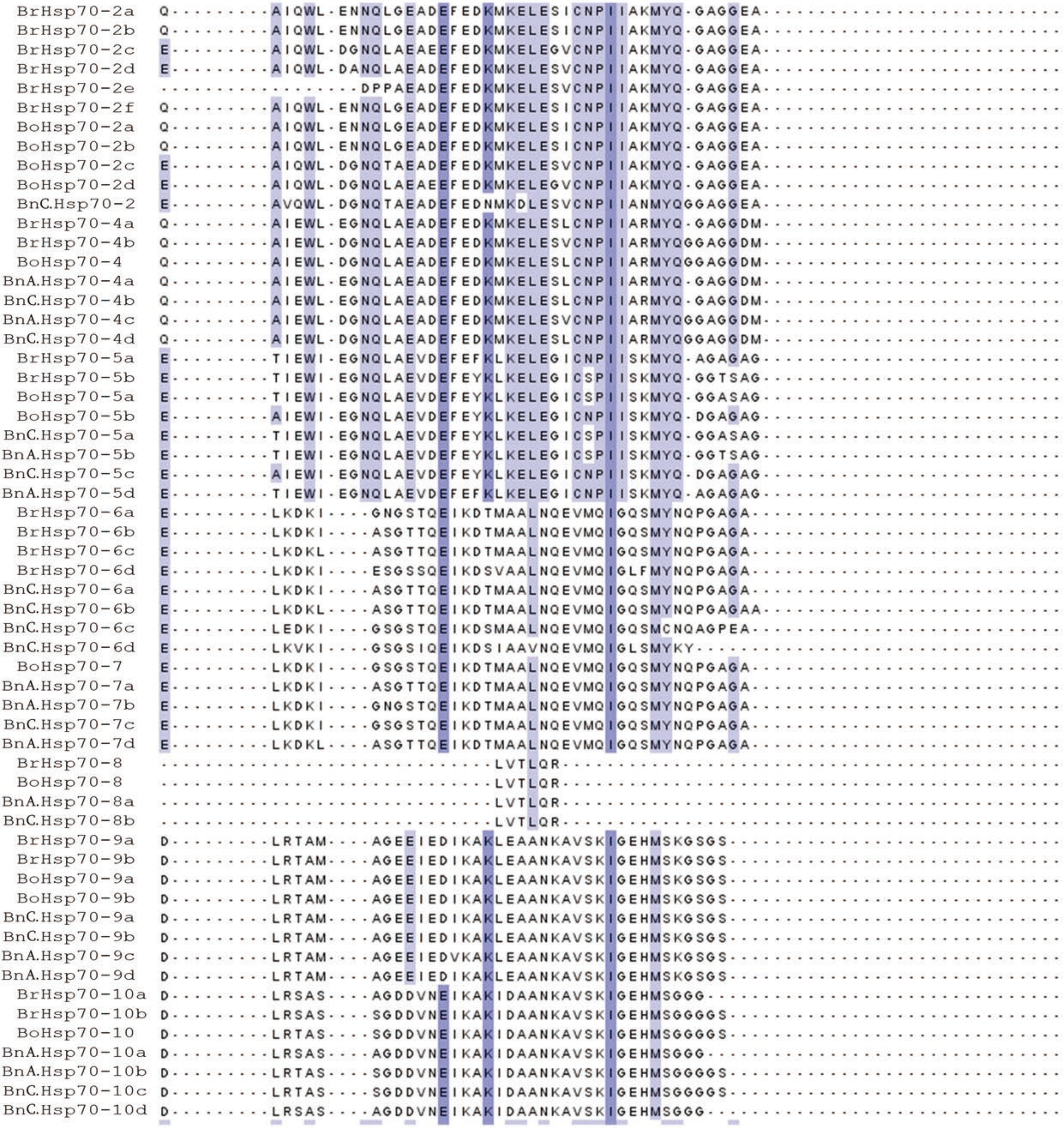
BrHsp70-2a Q---------AIQWL-ENNQLGEADEFEDKMKELESICNPIIAKMYQ-GAGGEA---------------------------
BrHsp70-2b Q---------AIQWL-ENNQLGEADEFEDKMKELESICNPIIAKMYQ-GAGGEA---------------------------
BrHsp70-2c E---------AIQWL-DGNQLAEAEEFEDKMKELEGVCNPIIAKMYQ-GAGGEA---------------------------
BrHsp70-2d E---------AIQWL-DANQLAEADEFEDKMKELESVCNPIIAKMYQ-GAGGEA---------------------------
BrHsp70-2e ------------------DPPAEADEFEDKMKELESVCNPIIAKMYQ-GAGGEA---------------------------
BrHsp70-2f Q---------AIQWL-ENNQLGEADEFEDKMKELESICNPIIAKMYQ-GAGGEA---------------------------
BoHsp70-2a Q---------AIQWL-ENNQLGEADEFEDKMKELESICNPIIAKMYQ-GAGGEA---------------------------
BoHsp70-2b Q---------AIQWL-ENNQLGEADEFEDKMKELESICNPIIAKMYQ-GAGGEA---------------------------
BoHsp70-2c E---------AIQWL-DGNQTAEADEFEDKMKELESVCNPIIAKMYQ-GAGGEA---------------------------
BoHsp70-2d E---------AIQWL-DGNQLAEAEEFEDKMKELEGVCNPIIAKMYQ-GAGGEA---------------------------
BnC.Hsp70-2 E---------AVQWL-DGNQTAEADEFEDNMKDLESVCNPIIANMYQGGAGGEA---------------------------
BrHsp70-4a Q---------AIEWL-EGNQLAEADEFEDKMKELESLCNPIIARMYQ-GAGGDM---------------------------
BrHsp70-4b Q---------AIEWL-DGNQLAEADEFEDKMKELESVCNPIIARMYQGGAGGDM---------------------------
BoHsp70-4 Q---------AIEWL-DGNQLAEADEFEDKMKELESLCNPIIARMYQGGAGGDM---------------------------
BnA.Hsp70-4a Q---------AIEWL-EGNQLAEADEFEDKMKELESLCNPIIARMYQ-GAGGDM---------------------------
BnC.Hsp70-4b Q---------AIEWL-EGNQLAEADEFEDKMKELESLCNPIIARMYQ-GAGGDM---------------------------
BnA.Hsp70-4c Q---------AIEWL-DGNQLAEADEFEDKMKELESVCNPIIARMYQGGAGGDM---------------------------
BnC.Hsp70-4d Q---------AIEWL-DGNQLAEADEFEDKMKELESLCNPIIARMYQGGAGGDM---------------------------
BrHsp70-5a E---------TIEWI-EGNQLAEVDEFEFKLKELEGICNPIISKMYQ-AGAGAG---------------------------
BrHsp70-5b E---------TIEWI-EGNQLAEVDEFEYKLKELEGICSPIISKMYQ-GGTSAG---------------------------
BoHsp70-5a E---------TIEWI-EGNQLAEVDEFEYKLKELEGICSPIISKMYQ-GGASAG---------------------------
BoHsp70-5b E---------AIEWI-EGNQLAEVDEFEYKLKELEGICNPIISKMYQ-DGAGAG---------------------------
BnC.Hsp70-5a E---------TIEWI-EGNQLAEVDEFEYKLKELEGICSPIISKMYQ-GGASAG---------------------------
BnA.Hsp70-5b E---------TIEWI-EGNQLAEVDEFEYKLKELEGICSPIISKMYQ-GGTSAG---------------------------
BnC.Hsp70-5c E---------AIEWI-EGNQLAEVDEFEYKLKELEGICNPIISKMYQ-DGAGAG---------------------------
BnA.Hsp70-5d E---------TIEWI-EGNQLAEVDEFEFKLKELEGICNPIISKMYQ-AGAGAG---------------------------
BrHsp70-6a E---------LKDKI----GNGSTQEIKDTMAALNQEVMQIGQSMYNQPGAGA----------------------------
BrHsp70-6b E---------LKDKI----ASGTTQEIKDTMAALNQEVMQIGQSMYNQPGAGA----------------------------
BrHsp70-6c E---------LKDKL----ASGTTQEIKDTMAALNQEVMQIGQSMYNQPGAGA----------------------------
BrHsp70-6d E---------LKDKI----ESGSSQEIKDSVAALNQEVMQIGLFMYNQPGAGA----------------------------
BnC.Hsp70-6a E---------LKDKI----ASGTTQEIKDTMAALNQEVMQIGQSMYNQPGAGA----------------------------
BnC.Hsp70-6b E---------LKDKL----ASGTTQEIKDTMAALNQEVMQIGQSMYNQPGAGAA---------------------------
BnC.Hsp70-6c E---------LEDKI----GSGSTQEIKDSMAALNQEVMQIGQSMCNQAGPEA----------------------------
BnC.Hsp70-6d E---------LKVKI----GSGSIQEIKDSIAAVNQEVMQIGLSMYKY---------------------------------
BoHsp70-7 E---------LKDKI----GSGSTQEIKDTMAALNQEVMQIGQSMYNQPGAGA----------------------------
BnA.Hsp70-7a E---------LKDKI----ASGTTQEIKDTMAALNQEVMQIGQSMYNQPGAGA----------------------------
BnA.Hsp70-7b E---------LKDKI----GNGSTQEIKDTMAALNQEVMQIGQSMYNQPGAGA----------------------------
BnC.Hsp70-7c E---------LKDKI----GSGSTQEIKDTMAALNQEVMQIGQSMYNQPGAGA----------------------------
BnA.Hsp70-7d E---------LKDKL----ASGTTQEIKDTMAALNQEVMQIGQSMYNQPGAGA----------------------------
BrHsp70-8 ------------------------------LVTLQR---
BoHsp70-8 ------------------------------LVTLQR---
BnA.Hsp70-8a ------------------------------LVTLQR---
BnC.Hsp70-8b ------------------------------LVTLQR---
BrHsp70-9a D---------LRTAM----AGEEIEDIKAKLEAANKAVSKIGEHMSKGSGS------------------------------
BrHsp70-9b D---------LRTAM----AGEEIEDIKAKLEAANKAVSKIGEHMSKGSGS------------------------------
BoHsp70-9a D---------LRTAM----AGEEIEDIKAKLEAANKAVSKIGEHMSKGSGS------------------------------
BoHsp70-9b D---------LRTAM----AGEEIEDIKAKLEAANKAVSKIGEHMSKGSGS------------------------------
BnC.Hsp70-9a D---------LRTAM----AGEEIEDIKAKLEAANKAVSKIGEHMSKGSGS------------------------------
BnC.Hsp70-9b D---------LRTAM----AGEEIEDIKAKLEAANKAVSKIGEHMSKGSGS------------------------------
BnA.Hsp70-9c D---------LRTAM----AGEEIEDVKAKLEAANKAVSKIGEHMSKGSGS------------------------------
BnA.Hsp70-9d D---------LRTAM----AGEEIEDIKAKLEAANKAVSKIGEHMSKGSGS------------------------------
BrHsp70-10a D---------LRSAS----AGDDVNEIKAKIDAANKAVSKIGEHMSGGG--------------------------------
BrHsp70-10b D---------LRSAS----SGDDVNEIKAKIDAANKAVSKIGEHMSGGGGS------------------------------
BoHsp70-10 D---------LRTAS----SGDDVNEIKAKIDAANKAVSKIGEHMSGGGGS------------------------------
BnA.Hsp70-10a D---------LRSAS----AGDDVNEIKAKIDAANKAVSKIGEHMSGGG--------------------------------
BnA.Hsp70-10b D---------LRTAS----SGDDVNEIKAKIDAANKAVSKIGEHMSGGGGS------------------------------
BnC.Hsp70-10c D---------LRTAS----SGDDVNEIKAKIDAANKAVSKIGEHMSGGGGS------------------------------
BnC.Hsp70-10d D---------LRSAS----AGDDVNEIKAKIDAANKAVSKIGEHMSGGG--------------------------------

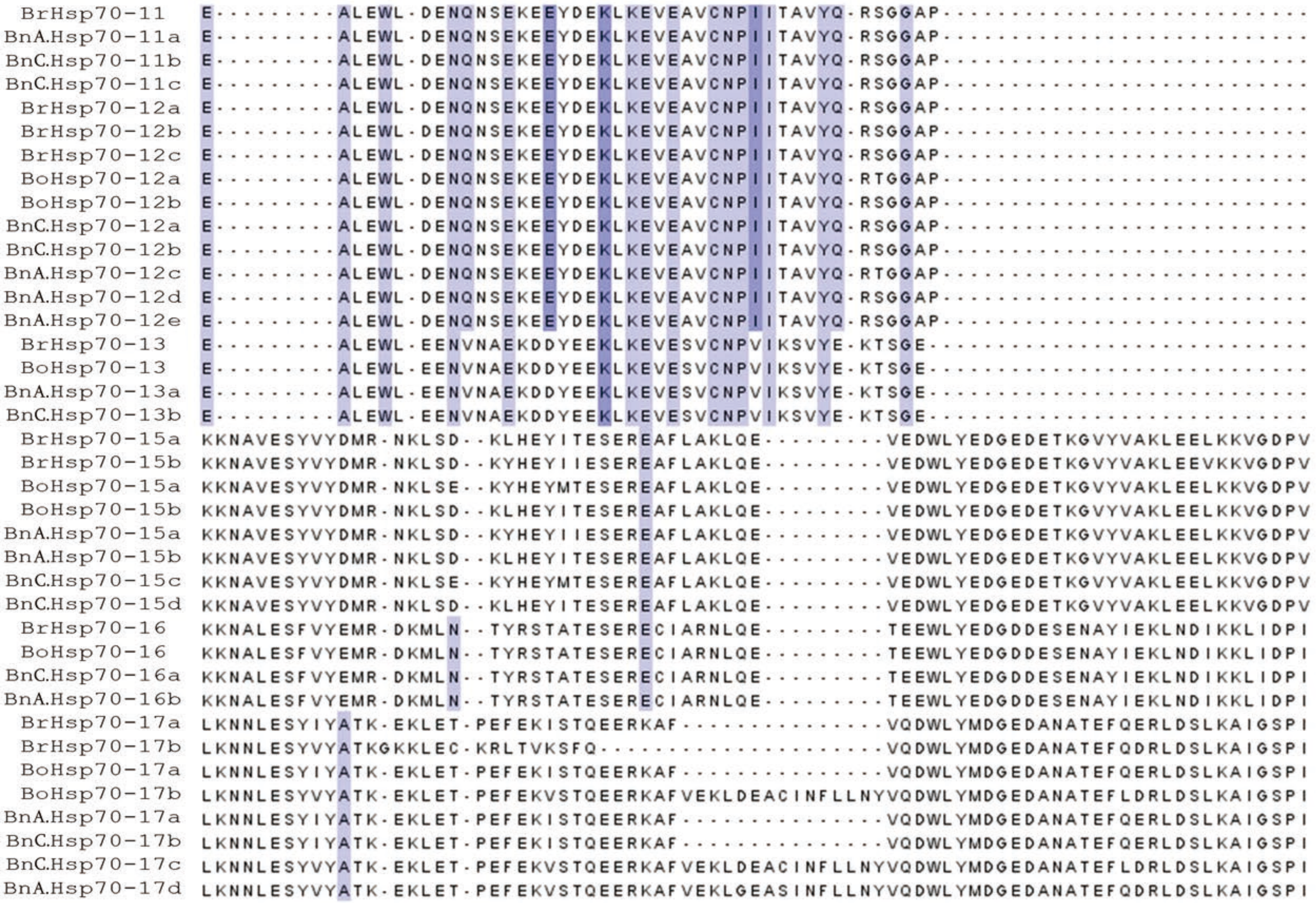
BrHsp70-11 E---------ALEWL-DENQNSEKEEYDEKLKEVEAVCNPIITAVYQ-RSGGAP---------------------------
BnA.Hsp70-11a E---------ALEWL-DENQNSEKEEYDEKLKEVEAVCNPIITAVYQ-RSGGAP---------------------------
BnC.Hsp70-11b E---------ALEWL-DENQNSEKEEYDEKLKEVEAVCNPIITAVYQ-RSGGAP---------------------------
BnC.Hsp70-11c E---------ALEWL-DENQNSEKEEYDEKLKEVEAVCNPIITAVYQ-RSGGAP---------------------------
BrHsp70-12a E---------ALEWL-DENQNSEKEEYDEKLKEVEAVCNPIITAVYQ-RSGGAP---------------------------
BrHsp70-12b E---------ALEWL-DENQNSEKEEYDEKLKEVEAVCNPIITAVYQ-RSGGAP---------------------------
BrHsp70-12c E---------ALEWL-DENQNSEKEEYDEKLKEVEAVCNPIITAVYQ-RSGGAP---------------------------
BoHsp70-12a E---------ALEWL-DENQNSEKEEYDEKLKEVEAVCNPIITAVYQ-RTGGAP---------------------------
BoHsp70-12b E---------ALEWL-DENQNSEKEEYDEKLKEVEAVCNPIITAVYQ-RSGGAP---------------------------
BnC.Hsp70-12a E---------ALEWL-DENQNSEKEEYDEKLKEVEAVCNPIITAVYQ-RSGGAP---------------------------
BnC.Hsp70-12b E---------ALEWL-DENQNSEKEEYDEKLKEVEAVCNPIITAVYQ-RSGGAP---------------------------
BnA.Hsp70-12c E---------ALEWL-DENQNSEKEEYDEKLKEVEAVCNPIITAVYQ-RTGGAP---------------------------
BnA.Hsp70-12d E---------ALEWL-DENQNSEKEEYDEKLKEVEAVCNPIITAVYQ-RSGGAP---------------------------
BnA.Hsp70-12e E---------ALEWL-DENQNSEKEEYDEKLKEVEAVCNPIITAVYQ-RSGGAP---------------------------
BrHsp70-13 E---------ALEWL-EENVNAEKDDYEEKLKEVESVCNPVIKSVYE-KTSGE----------------------------
BoHsp70-13 E---------ALEWL-EENVNAEKDDYEEKLKEVESVCNPVIKSVYE-KTSGE----------------------------
BnA.Hsp70-13a E---------ALEWL-EENVNAEKDDYEEKLKEVESVCNPVIKSVYE-KTSGE----------------------------
BnC.Hsp70-13b E---------ALEWL-EENVNAEKDDYEEKLKEVESVCNPVIKSVYE-KTSGE----------------------------
BrHsp70-15a KKNAVESYVYDMR-NKLSD--KLHEYITESEREAFLAKLQE---------VEDWLYEDGEDETKGVYVAKLEELKKVGDPV
BrHsp70-15b KKNAVESYVYDMR-NKLSD--KYHEYIIESEREAFLAKLQE---------VEDWLYEDGEDETKGVYVAKLEEVKKVGDPV
BoHsp70-15a KKNAVESYVYDMR-NKLSE--KYHEYMTESEREAFLAKLQE---------VEDWLYEDGEDETKGVYVAKLEELKKVGDPV
BoHsp70-15b KKNAVESYVYDMR-NKLSD--KLHEYITESEREAFLAKLQE---------VEDWLYEDGEDETKGVYVAKLEELKKVGDPV
BnA.Hsp70-15a KKNAVESYVYDMR-NKLSD--KYHEYIIESEREAFLAKLQE---------VEDWLYEDGEDETKGVYVAKLEELKKVGDPV
BnA.Hsp70-15b KKNAVESYVYDMR-NKLSD--KLHEYITESEREAFLAKLQE---------VEDWLYEDGEDETKGVYVAKLEELKKVGDPV
BnC.Hsp70-15c KKNAVESYVYDMR-NKLSE--KYHEYMTESEREAFLAKLQE---------VEDWLYEDGEDETKGVYVAKLEELKKVGDPV
BnC.Hsp70-15d KKNAVESYVYDMR-NKLSD--KLHEYITESEREAFLAKLQE---------VEDWLYEDGEDETKGVYVAKLEELKKVGDPV
BrHsp70-16 KKNALESFVYEMR-DKMLN--TYRSTATESERECIARNLQE---------TEEWLYEDGDDESENAYIEKLNDIKKLIDPI
BoHsp70-16 KKNALESFVYEMR-DKMLN--TYRSTATESERECIARNLQE---------TEEWLYEDGDDESENAYIEKLNDIKKLIDPI
BnC.Hsp70-16a KKNALESFVYEMR-DKMLN--TYRSTATESERECIARNLQE---------TEEWLYEDGDDESENAYIEKLNDIKKLIDPI
BnA.Hsp70-16b KKNALESFVYEMR-DKMLN--TYRSTATESERECIARNLQE---------TEEWLYEDGDDESENAYIEKLNDIKKLIDPI
BrHsp70-17a LKNNLESYIYATK-EKLET-PEFEKISTQEERKAF---------------VQDWLYMDGEDANATEFQERLDSLKAIGSPI
BrHsp70-17b LKNNLESYVYATKGKKLEC-KRLTVKSFQ---------------------VQDWLYMDGEDANATEFQDRLDSLKAIGSPI
BoHsp70-17a LKNNLESYIYATK-EKLET-PEFEKISTQEERKAF---------------VQDWLYMDGEDANATEFQERLDSLKAIGSPI
BoHsp70-17b LKNNLESYVYATK-EKLET-PEFEKVSTQEERKAFVEKLDEACINFLLNYVQDWLYMDGEDANATEFLDRLDSLKAIGSPI
BnA.Hsp70-17a LKNNLESYIYATK-EKLET-PEFEKISTQEERKAF---------------VQDWLYMDGEDANATEFQERLDSLKAIGSPI
BnC.Hsp70-17b LKNNLESYIYATK-EKLET-PEFEKISTQEERKAF---------------VQDWLYMDGEDANATEFQERLDSLKAIGSPI
BnC.Hsp70-17c LKNNLESYVYATK-EKLET-PEFEKVSTQEERKAFVEKLDEACINFLLNYVQDWLYMDGEDANATEFLDRLDSLKAIGSPI
BnA.Hsp70-17d LKNNLESYVYATK-EKLET-PEFEKVSTQEERKAFVEKLGEASINFLLNYVQDWLYMDGEDANATEFQDRLDSLKAIGSPI

```
BrHsp70-2a    ............................................................
BrHsp70-2b    ............................................................
BrHsp70-2c    ............................................................
BrHsp70-2d    ............................................................
BrHsp70-2e    ............................................................
BrHsp70-2f    ............................................................
BoHsp70-2a    ............................................................
BoHsp70-2b    ............................................................
BoHsp70-2c    ............................................................
BoHsp70-2d    ............................................................
BnC.Hsp70-2   ............................................................
BrHsp70-4a    ............................................................
BrHsp70-4b    ............................................................
BoHsp70-4     ............................................................
BnA.Hsp70-4a  ............................................................
BnC.Hsp70-4b  ............................................................
BnA.Hsp70-4c  ............................................................
BnC.Hsp70-4d  ............................................................
BrHsp70-5a    ............................................................
BrHsp70-5b    ............................................................
BoHsp70-5a    ............................................................
BoHsp70-5b    ............................................................
BnC.Hsp70-5a  ............................................................
BnA.Hsp70-5b  ............................................................
BnC.Hsp70-5c  ............................................................
BnA.Hsp70-5d  ............................................................
BrHsp70-6a    ............................................................
BrHsp70-6b    ............................................................
BrHsp70-6c    ............................................................
BrHsp70-6d    ............................................................
BnC.Hsp70-6a  ............................................................
BnC.Hsp70-6b  ............................................................
BnC.Hsp70-6c  ............................................................
BnC.Hsp70-6d  ............................................................
BoHsp70-7     ............................................................
BnA.Hsp70-7a  ............................................................
BnA.Hsp70-7b  ............................................................
BnC.Hsp70-7c  ............................................................
BnA.Hsp70-7d  ............................................................
BrHsp70-8     ............................................................
BoHsp70-8     ............................................................
BnA.Hsp70-8a  ............................................................
BnC.Hsp70-8b  ............................................................
BrHsp70-9a    ............................................................
BrHsp70-9b    ............................................................
BoHsp70-9a    ............................................................
BoHsp70-9b    ............................................................
BnC.Hsp70-9a  ............................................................
BnC.Hsp70-9b  ............................................................
BnA.Hsp70-9c  ............................................................
BnA.Hsp70-9d  ............................................................
BrHsp70-10a   ............................................................
BrHsp70-10b   ............................................................
BoHsp70-10    ............................................................
BnA.Hsp70-10a ............................................................
BnA.Hsp70-10b ............................................................
BnC.Hsp70-10c ............................................................
BnC.Hsp70-10d ............................................................
```

```
BrHsp70-11       ................................................................................
BnA.Hsp70-11a    ................................................................................
BnC.Hsp70-11b    ................................................................................
BnC.Hsp70-11c    ................................................................................
BrHsp70-12a      ................................................................................
BrHsp70-12b      ................................................................................
BrHsp70-12c      ................................................................................
BoHsp70-12a      ................................................................................
BoHsp70-12b      ................................................................................
BnC.Hsp70-12a    ................................................................................
BnC.Hsp70-12b    ................................................................................
BnA.Hsp70-12c    ................................................................................
BnA.Hsp70-12d    ................................................................................
BnA.Hsp70-12e    ................................................................................
BrHsp70-13       ................................................................................
BoHsp70-13       ................................................................................
BnA.Hsp70-13a    ................................................................................
BnC.Hsp70-13b    ................................................................................
BrHsp70-15a      EMRYKESQERGTVIGQLGHCVNSYRE----------------------AAVSNDSKFDHIELEEKQKVLNECVEAEAWMR
BrHsp70-15b      EMRYKESQERGTVIGQLGHCVNSYRE----------------------AAVSNDSKFDHIELEDKQKVLNECVEAEAWMR
BoHsp70-15a      EMRYKESQERGTVIGQLGHCVNSYRE----------------------AAVSNDSKFDHIELEEKQKVLNECVEAEAWMR
BoHsp70-15b      EMRYKESQERGTVIGQLGHCVNSYRE----------------------AAVSNESKFDHIELEEKQKVLNECVEAEAWMR
BnA.Hsp70-15a    EMRYKESQERGTVIGQLGHCVNSYRE----------------------AAVSNDSKFDHIELEDKQKVLNECVEAEAWMR
BnA.Hsp70-15b    EMRYKESQERGTVIGQLGHCVNSYRE----------------------AAVSNDSKFDHIELEEKQKVLNECVEAEAWMR
BnC.Hsp70-15c    EMRYKESQERGTVIGQLGHCVNSYRE----------------------AAVSNDSKFDHIELEEKQKVLNECVEAEAWMR
BnC.Hsp70-15d    EMRYKESQERGTVIGQLGHCVNSYRE----------------------AAVSNDS--NHIELEEKQKVLNECVEAEAWMR
BrHsp70-16       ENRFKDGEERVQASKDLAKTIADNRM----------------------AAES-------LPPPRKNAVLDECKKVERWLH
BoHsp70-16       ENRYKDAEERVQASKDLAKTIADNRM----------------------AAES-------LPPPRKNAVLDECKKVERWLH
BnC.Hsp70-16a    ENRYKDAEERVQASKDLAKTIADNRM----------------------AAES-------LPPPRKNAVLDECKKVERWLH
BnA.Hsp70-16b    ENRFKDGEERVQASKDLAKTIADNRM----------------------AAES-------LPPPRKNAVLDECKKVERWLH
BrHsp70-17a      SLRSDELTARPVAVEYAQKYLTEVKE----------------------IIKEWETNKTWLPKEKINEVSKEAEKVKSWLE
BrHsp70-17b      SLRSEELTARPVAVEYAQKYLTEVKEVHFAVSHDKEGINPAEFQPHLQIIKEWETNKTWLPKEKIDQ-------------
BoHsp70-17a      SLRSDELTARPVAVEYAQKYLTEVKE----------------------IIKEWETNKTWLPKEKINEVSKEAEKVKSWLE
BoHsp70-17b      SLRSEELTARPVAVEYAQKYLTEVKE----------------------IIKEWETNKTWLPKGKIDEVSKEAEKVKSWLE
BnA.Hsp70-17a    SLRSDELTARPVAVEYAQKYLTEVKE----------------------IIKEWETNKTWLPKEKINEVSKEAEKVKSWLE
BnC.Hsp70-17b    SLRSDELTARPVAVEYAQKYLTEVKE----------------------IIKEWETNKTWLPKEKINEVSKEAEKVKSWLE
BnC.Hsp70-17c    SLRSEELTARPVAVEYAQKYLTEVKE----------------------IIKEWETNKTWLPRGKIDEVSKEAEKVKSWLE
BnA.Hsp70-17d    SLRSEELTARPVAVEYAQKYLTEVKE----------------------IIKEWETNKTWLPKEKIDQVSKEAEKVKSWLE
```

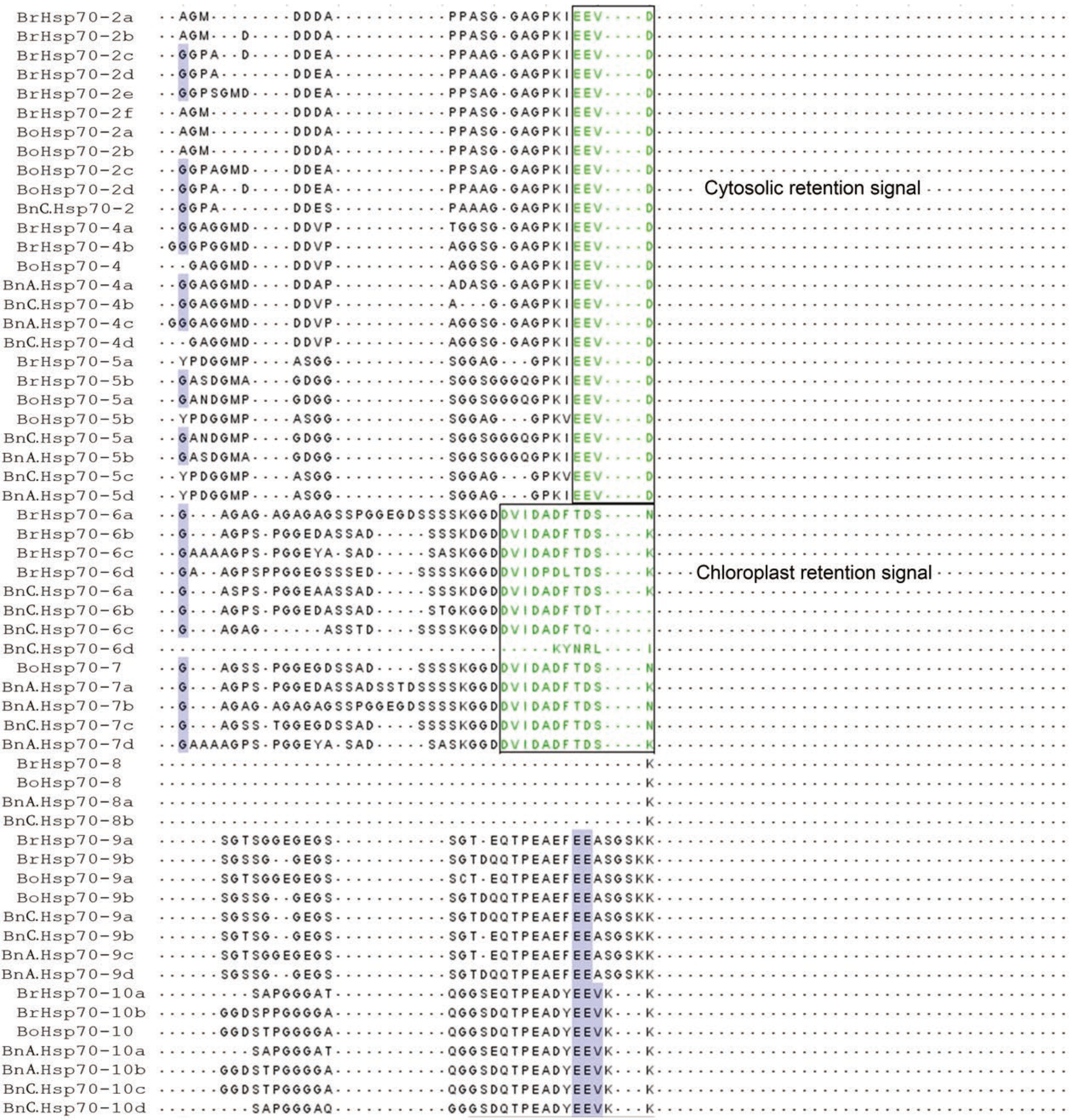
Cytosolic retention signal
Chloroplast retention signal

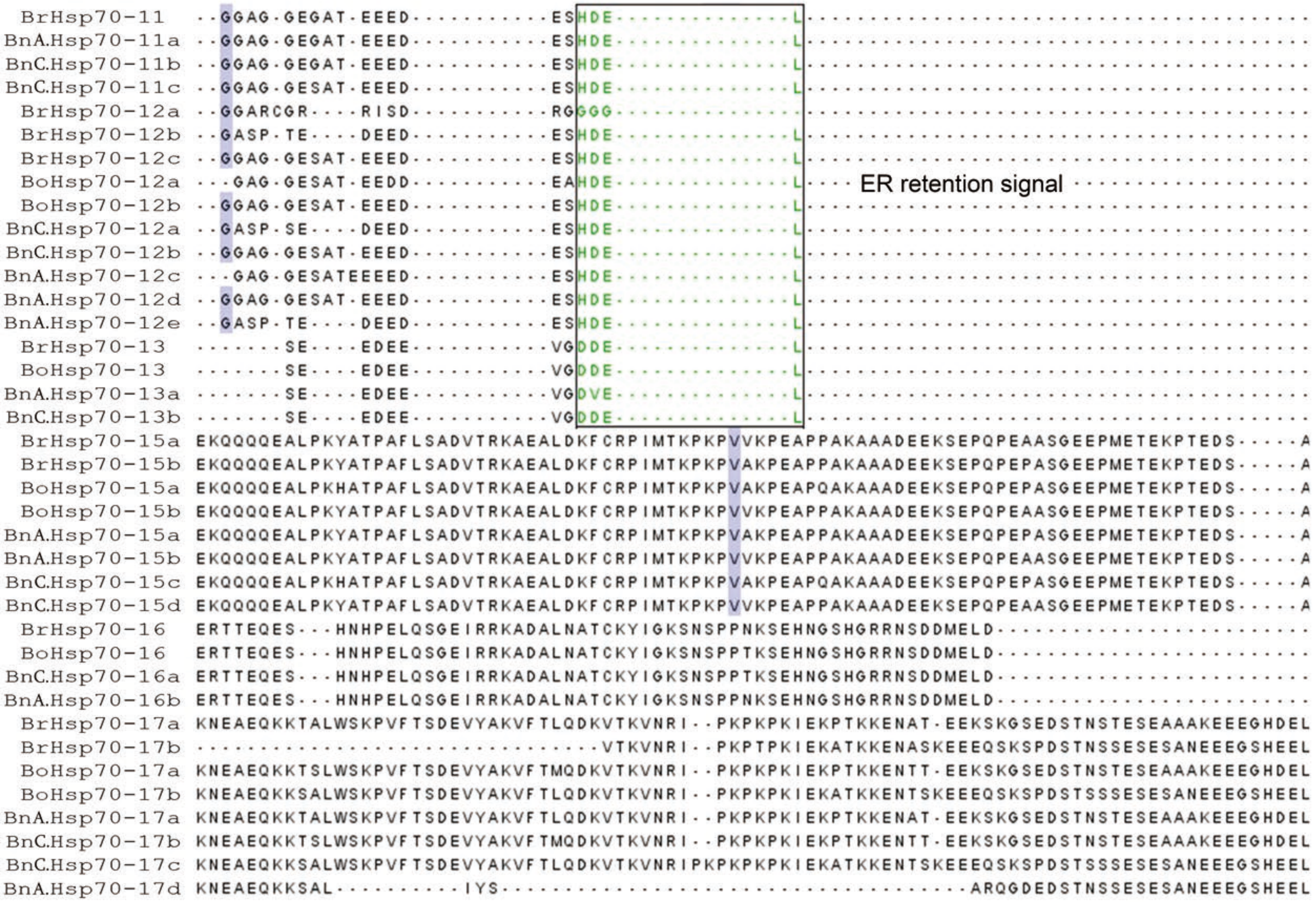
BrHsp70-11
BnA.Hsp70-11a
BnC.Hsp70-11b
BnC.Hsp70-11c
BrHsp70-12a
BrHsp70-12b
BrHsp70-12c
BoHsp70-12a
BoHsp70-12b
BnC.Hsp70-12a
BnC.Hsp70-12b
BnA.Hsp70-12c
BnA.Hsp70-12d
BnA.Hsp70-12e
BrHsp70-13
BoHsp70-13
BnA.Hsp70-13a
BnC.Hsp70-13b
BrHsp70-15a
BrHsp70-15b
BoHsp70-15a
BoHsp70-15b
BnA.Hsp70-15a
BnA.Hsp70-15b
BnC.Hsp70-15c
BnC.Hsp70-15d
BrHsp70-16
BoHsp70-16
BnC.Hsp70-16a
BnA.Hsp70-16b
BrHsp70-17a
BrHsp70-17b
BoHsp70-17a
BoHsp70-17b
BnA.Hsp70-17a
BnC.Hsp70-17b
BnC.Hsp70-17c
BnA.Hsp70-17d
ER retention signal
