# Supplementary figures and images for: Genome-wide identification and characterization of the Hsp70 gene family in allopolyploid rapeseed (Brassica napus L.) compared with its diploid progenitors
Source: PeerJ. 2019 Aug 20;7:e7511. doi: 10.7717/peerj.7511 (PMC6707343; doi:10.7717/peerj.7511)

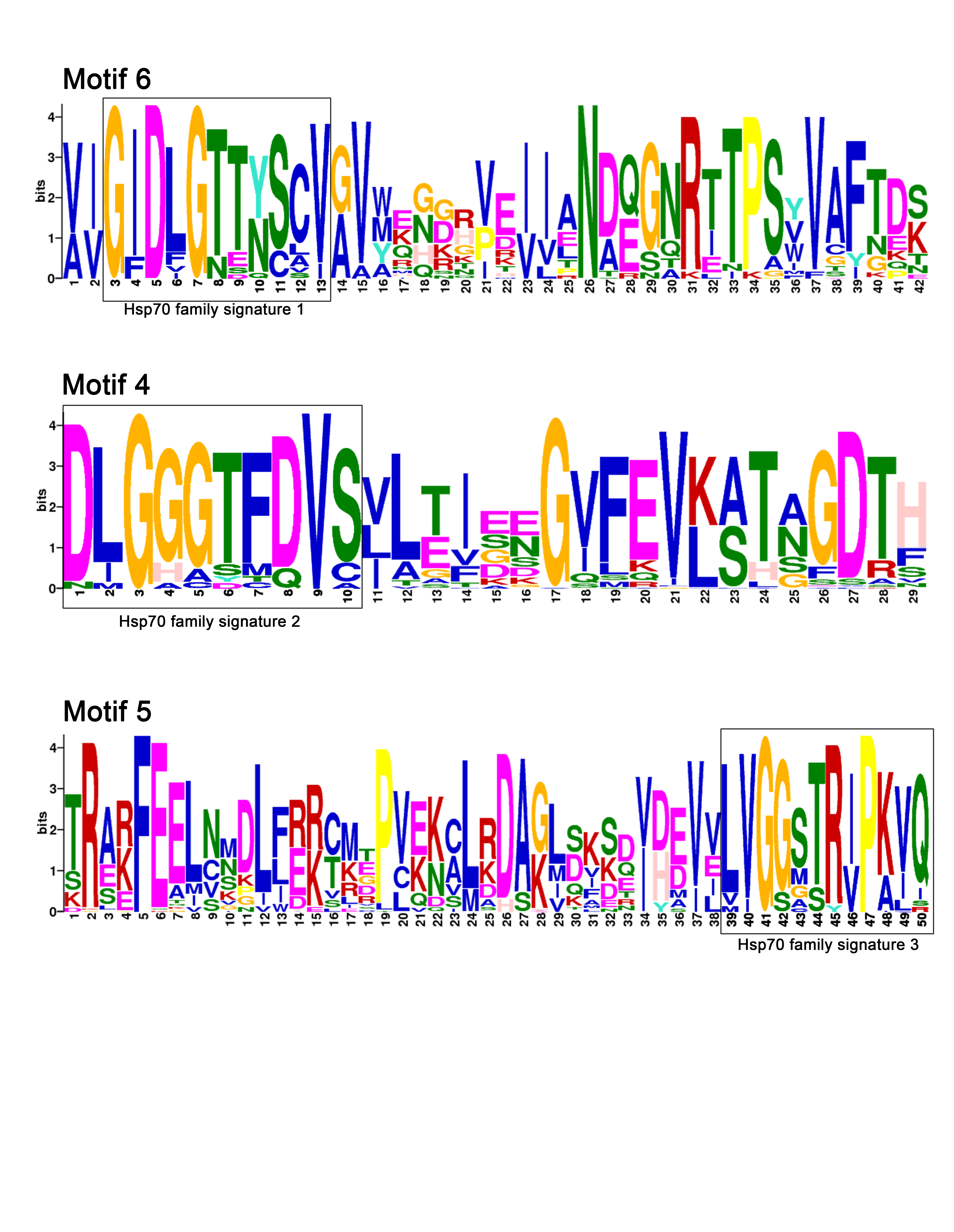

Supplement: Figure S2 — The bits indicate amino acid conservation in each positon. GIDLGTT (N/Y) SCV sequences in motif 6, DLGGGTFDVS sequences in motif 4 and LVGG (S) TR (I) PKVQ sequences in motif 5 are highlighted with black boxes. [file peerj-07-7511-s002.png]
